# 7-Acetoxyhorminone from *Salvia multicaulis* Vahl. as Promising Inhibitor of 3-Hydroxy-3-methylglutaryl Coenzyme A (HMG-CoA) Reductase

**DOI:** 10.3390/ph15020198

**Published:** 2022-02-04

**Authors:** Serkan Yigitkan, Abdulselam Ertas, Ramin Ekhteiari Salmas, Mehmet Firat, Ilkay Erdogan Orhan

**Affiliations:** 1 Department of Pharmaceutical Botany, Faculty of Pharmacy, Dicle University, 21200 Diyarbakir, Turkey; syigitkan@dicle.edu.tr; 2 Department of Pharmacognosy, Faculty of Pharmacy, Gazi University, 06330 Ankara, Turkey; 3 Department of Analytical Chemistry, Faculty of Pharmacy, Dicle University, 21200 Diyarbakir, Turkey; abdulselamertas@hotmail.com; 4 Cancer Research Center, Dicle University, 21200 Diyarbakir, Turkey; 5 Department of Chemistry, Britannia House, King’s College, London SE1 1DB, UK; ramin.ekhteiari@gmail.com; 6 Department of Biology, Faculty of Education, Van Yuzuncu Yil University, 65080 Van, Turkey; mehmetfirat@yyu.edu.tr

**Keywords:** hypercholesterolemia, HMG-CoA reductase, enzyme inhibition, *Salvia*, terpenoids

## Abstract

3-Hydroxy-3-methylglutaryl coenzyme A (HMG-CoA) reductase is a key enzyme involved in cholesterol biosynthesis and one of the most important targets for the treatment of hypercholesterolemia. A limited number of studies on the HMG-CoA reductase inhibitory potential of natural products are available. Thus, in the current study, we aimed to test the HMG-CoA reductase inhibitory capacity of extracts from the roots and aerial parts of *Salvia multicaulis* Vahl., through activity-guided isolation. Our findings revealed that the root extract prepared with dichloromethane–acetone (1:1) showed the highest inhibition (71.97 ± 0.37%) at 100 µg/mL. The extract was then initially fractionated by column chromatography and the obtained fractions were monitored by thin layer chromatography. Fractions which were similar to each other were combined and a total of 15 fractions were obtained. Further conventional chromatographic studies were carried out on the active fractions. Based on these fractions, 10 known compounds, comprising 9 terpenes and 1 steroid derivative in total, were isolated and their structures were verified by a combination of IT-TOF-MS, and 1D and 2D NMR techniques. According to the enzyme inhibition data of the identified compounds, 7-acetoxyhorminone exerted the highest inhibition (84.15 ± 0.10%, IC_50_ = 63.6 ± 1.21 µg/mL). The molecular docking experiments on 7-acetoxyhorminone and horminone indicated that both compounds strongly bind to the active site of the enzyme.

## 1. Introduction

Cholesterol is an important biomolecule found in the composition of the cell membranes of all eukaryotic life, which is necessary for growth and the continuation of vital activities in the organism [1]. Hypercholesterolemia, known as high cholesterol, is a common disease, in which excess fat and fatty acids accumulate in the blood [2]. Hypercholesterolemia is one of the major risk factors that cause atherosclerosis and coronary heart diseases [3]. Especially increased low-density lipoprotein (LDL) and triglyceride levels, which lay the basis for hypercholesterolemia, also lead to diseases, such as obesity, diabetes, and cancer [4]. HMG-CoA (3-hydroxy-3-methylglutaryl coenzyme A) reductase is an enzyme known to catalyze the reaction in which HMG-CoA is converted to mevalonate, through the mevalonate pathway during cholesterol biosynthesis. It is the rate-limiting enzyme that regulates cholesterol synthesis in the body [5]. Therefore, the inhibition of HMG-CoA reductase is an important therapeutic strategy for the treatment of hypercholesterolemia. Relevant to this point, statins, as the most highly reputed anti-hypercholesterolemic drug class, have been used against hypercholesterolemia for a long time. However, it is well-known that the long-term use of statins causes serious side effects, such as myopathy, rhabdomyolysis, liver enzyme dysfunction, sexual dysfunctions, and hepatotoxicity. Due to the mentioned side effects of statin derivatives, there is still a distinct need for the discovery of new, effective, and safer HMG-CoA reductase inhibitors [5,6,7].

Alternately, natural products and medicinal plants have always been a target for the discovery of novel drug candidates. Among them, *Salvia* L. is one of the most common genera of the Lamiaceae family, represented by approximately 900 species in the world [8], while it is represented by 101 species in Turkey, approximately 30% of which are endemic [9]. In the folk medicines of various countries, *Salvia* species are widely used against colds, stomach complaints, sore throat, inflammatory skin disorders, and wounds, as well as to stop bleeding [10,11,12,13,14]. A huge number of phytochemical and biological studies on *Salvia* species growing in Turkey have been carried out since 1968. In those studies, many newly discovered and known compounds from different chemical classes, such as flavonoids, terpenoids, phenolic acids, phenolic glycosides, and various others, were isolated [15]. In addition, a wide spectrum of bioactivities, e.g., antioxidant, antimicrobial, wound healing, cytotoxic, antiangiogenic, etc., of the isolated compounds and extracts from *Salvia* taxa have been reported [16]. It should also be noted that various *Salvia* species (*S. virgata* Jacq., *S. verticillata* subsp. *amasiaca* (Freyn and Bornm.) Bornm., *S. miltihorrhiza* Bunge, *S. multicaulis* Vahl., and *S. digitaloides* Diels) are traditionally used in Anatolian folk medicine, and in some other countries against heart diseases [17,18,19,20].

Natural products have led the way in the discovery of new HMG-CoA reductase inhibitors, as well as in the discovery of other drugs. Compactin (identical to mevastatin/6-demethylmevinolin), isolated from blue–green mold, i.e., *Penicillium brevicompatum*, in 1973, was the first example of a natural product with evident HMG-CoA reductase inhibition. This was followed by monacolin K, isolated from *Monascus ruber* in 1978, which was identical to lovastatin (mevinolin) separated from another microfungus, i.e., *Aspergillus terreus*, as well as *M. ruber* [21]. Lovastatin was later discovered from *A. terreus* [22]. Taking the folkloric use of the aforementioned *Salvia* species against heart diseases, and the discovery of the first-line statin derivatives from natural sources into consideration, *S. multicaulis* was examined in terms of its possible HMG-CoA reductase inhibitory activity in the current study. It should be noted that this is the first time HMG-CoA reductase inhibitory activity-guided isolation studies on a *Salvia* species have been performed. For this purpose, extracts from various polarities of the root and aerial parts of *S. multicaulis* were prepared and activity-guided fractionation was performed on the active extract. Consequently, 10 compounds in total, one of which was a mixture, were isolated from the enzyme-inhibiting fractions, and the HMG-CoA reductase inhibitory activities of the isolated compounds were determined. The active inhibitory molecules were progressed to in silico experiments to discover the interactions with the enzyme.

## 2. Results

### 2.1. Isolation of the Compounds

6,7-Dehydroyleanone (6 mg, orange crystal) (**1**), 12-demethylmulticaulin (8 mg, light orange crystal) (**2**), ferruginol (12 mg, white crystal) (**3**), and 12-hydroxy abieta-1, 3, 5 (**10**), 8, 11, 13-hexaene (5 mg, light orange crystal) (**4**), were obtained using preparative TLC from fraction M-3 (petroleum ether–dichloromethane: 1/1, 1/1, 1/2, and 1/2 solvent systems, respectively). β-Sitosterol, as a white powder (8 mg) (**5**), and a mixture of horminone and 7-acetoxyhorminone (1:1) (15 mg, orange crystal) (**6**), were isolated from the M-6 fraction (petroleum ether–dichloromethane: 1/2 solvent system) with the help of preparative thin layer chromatography (TLC). 7-Acetoxyhorminone (**7**) was isolated from the M-7 fraction as an orange crystal (10 mg) using a Sephadex column, followed by preparative TLC (petroleum ether–dichloromethane: 1/3). Pisiferal (**8**) was isolated from the M-8 fraction as a yellowish orange crystal, using a Sephadex column, followed by preparative TLC (dichloromethane–acetone: 1/1). Ursolic acid (12 mg) (**9**) and oleanolic acid (15 mg) (**10**) were isolated in a white powder form from fraction M-10 using preparative TLC (dichloromethane–acetone: 1/1) [6].

### 2.2. Structure Elucidation

**Figure 1:**
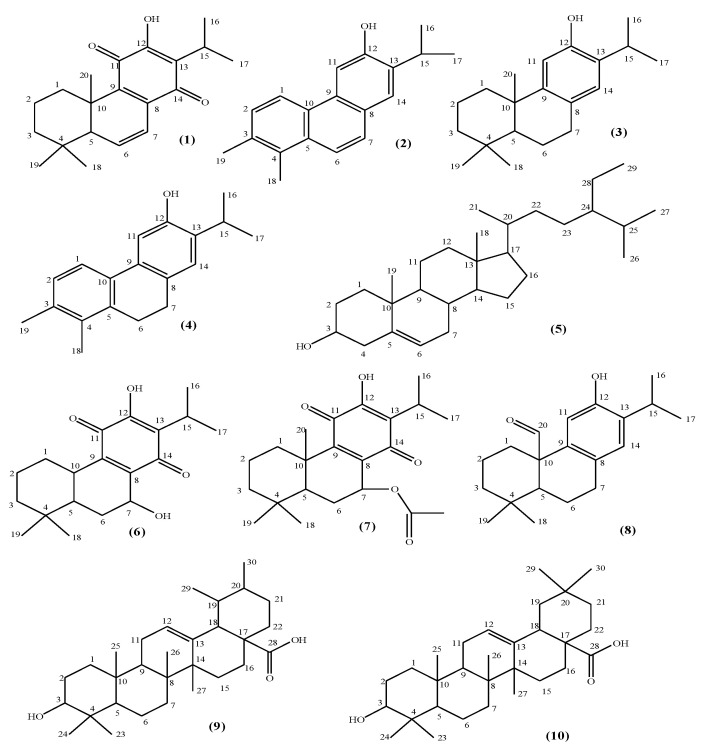
Chemical formulae of the isolated compounds: 6,7-dehydroyleanone (**1**), 12-demethylmulticaulin (**2**), ferruginol (**3**), 12-hydroxy abieta-1, 3, 5(**10**), 8, 11, 13-hexaene (**4**), β-sitosterol (**e5**), horminone (**6**), 7-acetoxyhorminone (**7**), pisiferal (**8**), ursolic acid (**9**), and oleanolic acid (**10**).

Our sequential chromatographic experiments on the active fractions led to the isolation of 10 compounds, one of which was a mixture (Figure 1). Our structure elucidation studies indicated that five of them were abietane-type diterpenes (6,7-dehydroyleanone (**1**) [23], ferruginol (**3**) [24], horminone–7-acetoxyhorminone mixture (1:1) (**6**) (which were previously isolated from several *Salvia* species), 7-acetoxyhorminone (**7**) [25,26,27], and pisiferal (**8**) [28]); two nor abietane-type diterpenes (12-demethylmulticaulin (**2**) [29,30], and 12-hydroxy abieta-1, 3, 5 (**10**) 8, 11, 13-hexaene (**4**) [30,31]); one steroid (β-sitosterol (**5**) [32]); and two triterpenes (ursolic acid (**9**) [32] and oleanolic acid (**10**) [32]). Their structures were revealed using a combination of spectroscopic methods, e.g., UV, IR, ^1^H- and ^13^C-NMR-APT, HMQC, HMBC, and mass spectrometry (MS). The spectroscopic data of the isolated compounds were also compared with those given in the literature, which led to a final definite structural confirmation of all the isolated compounds. Spectral data of the compounds is presented in supplementary material file (Figures S1–S53).

### 2.3. HMG-CoA Reductase Inhibitory Activity of the Isolated Compounds

In this study, the extracts of the root and aerial parts of the plant in different polarities were initially subjected to enzyme inhibition assays. Then, activity-guided fractionation was performed on the most active extract, i.e., dichloromethane–acetone (1:1). The obtained fractions were combined and again subjected to enzyme inhibition assays (Table 1).

Since five fractions, i.e., M-3, M-6, M-7, M-8, and M-10, exhibited inhibition over 50%, it was decided that studies should be continued on them. The HMG-CoA reductase inhibitory activity of the compounds isolated from the active fractions was also determined. Atorvastatin was used as the reference drug, while horminone–7-acetoxyhorminone mixture (1:1) (**6**) and 7-acetoxyhorminone (**7**) were found to possess the highest inhibition (Table 2).

### 2.4. Molecular Docking Data

**Figure 2:**
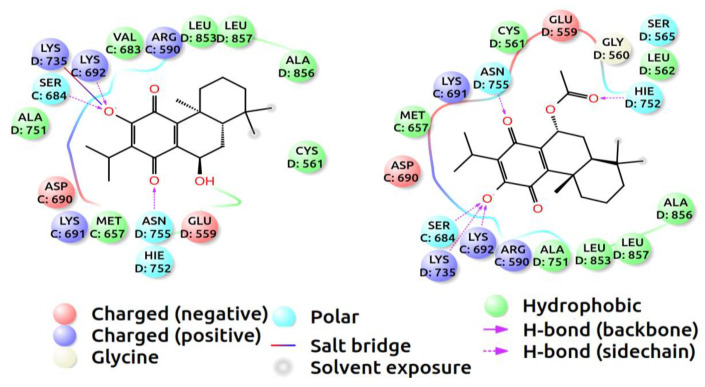
Two-dimensional ligand diagrams of horminone–7-acetoxyhorminone (**left**) and 7-acetoxyhorminone (**right**). The chemical properties of the amino acids are represented by different colors.

A molecular docking simulation has been found useful to obtain a better understanding of the inhibition mechanisms of small molecules—including how the ligands stick to the catalytic domains of receptors, which amino acids are more likely to contribute to stabilizing the ligands, and the chemical bonds that would be formed between the ligands and receptor. In this section, the binding positions and the free energies of the two compounds, horminone–7-acetoxyhorminone and 7-acetoxyhorminone, inside the active site of HMG-CoA reductase, were determined using the docking simulation method. The neighboring amino acids surrounding and stabilizing the ligands of horminone–7-acetoxyhorminone (**1**) and 7-acetoxyhorminone (**2**), by forming polar and non-polar interactions, were displayed with 2D diagrams in Figure 2. The amino acids, i.e., Ser684, Lys692, Lys735, Hie752, and Asn755, have been found to play a dominant role in bonding the ligands to the receptor. Both systems suffer from the effect of less aromatic interactions, since neither of the aromatic domains in the ligands is likely to form strong pi–pi stacking or hydrophobic bonds with the amino acids in the binding domain, which can result in an increase in free energy.

**Figure 3:**
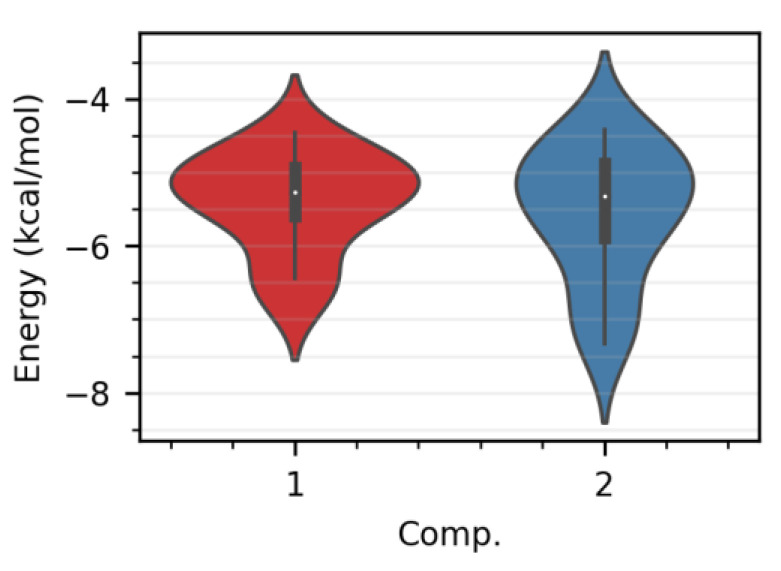
Distribution of the binding energies of horminone–7-acetoxyhorminone (**1**) and 7-acetoxyhorminone (**2**).

More than one binding energy has been calculated for every single ligand, following the fact that ligands inside the binding cavity can fall into diverse poses with different binding energies, as shown in Figure 3. The distribution of the binding energies in the boxplots were classified as the minimum, first quartile, median, third quartile, and maximum scores. A convergence of the median scores for both compounds, falling under −5 kcal/mol, has been observed, which demonstrates the ability of the ligands to stick to the binding site of the receptor. Compound 2 has been found to have a wider interquartile range and a lower minimum score, of under −7 kcal/mol, compared to compound 1. Compound 2 has been able to pose more diverse conformers with lower binding energies during sticking to the binding site, due to more polar groups in the structure, which, in turn, form more polar bonds with the amino acids.

## 3. Discussion

Cardiovascular disease, hypercholesterolemia in particular, is among the leading health problems in the world. It ranks first among the causes of death in the USA [33]. The most prescribed drug group in the current treatment of hypercholesterolemia is statins, which act through the HMG-CoA reductase inhibitory mechanism [34,35,36]. Although statins are considered the most effective drug class against hypercholesterolemia, they have various adverse effects, such as myopathy, rhabdomyolysis, and increased liver enzyme levels. In addition, findings have pointed to the fact that they increase the incidence of type-2 diabetes [37,38,39,40,41,42,43]. According to our detailed literature research, only a few studies on the inhibitory potential of *Salvia* L. taxa on HMG-CoA reductase have so far been reported, which inspired us to conduct the present research. In one of the previous studies, a traditional Chinese drug consisting of *S. miltiorrhiza* Bunge and *Carthamus tinctorius* L., was found to reduce HMG-CoA reductase mRNA expression in female ApoE-/- and LDLR-/- type mice [44]. In another study, it was reported that the protein fraction obtained from *S. hispanica* L., known as “chia”, inhibited HMG-CoA reductase [45]. As mentioned, a limited number of reports are available on the HMG-CoA reductase inhibitory capacity of natural molecules and medicinal herbs. Therefore, more research on natural sources to find lead compounds is needed. In this context, we can conclude that the abietane-type diterpenes, i.e., horminone and horminone–7-acetoxyhorminone (1:1), were the leading compounds responsible for the HMG-CoA reductase inhibitory activity of *S. multicaulis* root extract. The HMG-CoA reductase inhibitory effects of diterpenes against the mentioned enzyme have been reported in some studies, such as *Polyalthia longifolia* (Sonn.) Thwaites [46]. For instance, 16α-hydroxycleroda-3,13(14)*Z*-diene-15,16-olide, a clerodane-type diterpene isolated from pendula, has been described as a “*new class of HMG-CoA reductase inhibitory natural compounds*” due to its potent inhibition [6,46,47]. It has been determined that the diterpene-derivative compound called cafestol in coffee, inhibited HMG-CoA reductase by 40% at a concentration of 20 µg/mL [48].

**Figure 4:**
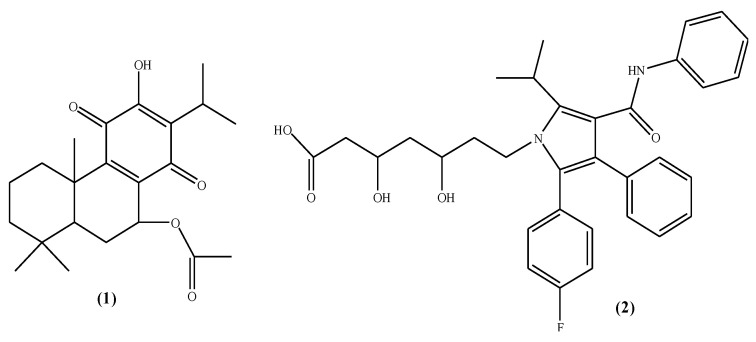
Chemical formulae of the isolated compounds, i.e., 7-acetoxyhorminone (**1**) and atorvastatin (**2**).

The early discovered statins, i.e., mevastatin and lovastatin, became model parent molecules in the discovery of new HMG-CoA reductase inhibitors. Guided by this structural similarity, simvastatin, pravastatin, cerivastatin, pitavastatin, etc., were synthesized as novel inhibitors. The hexahydronaphthalene ring systems in mevastatin and lovastatin, as well as monacolins, were reported to be similar to the HMG part of the enzyme that induces the activity through binding to the domain of the active gorge in HMG-CoA reductase [49]. A polyketide substitution is also common in natural statins [50]. In this study, 7-acetoxyhorminone (Figure 4) was predicted to exhibit strong bonding through its decalin ring structure, while the fluorophenyl group of the atorvastatin molecule, which was used as the reference, was thought to play an active role in the binding. However, it can be predicted that the methyl groups provide the Van der Waals interaction, while the hydroxyl functional groups that are common in both structures, interact with the active site of the enzyme through hydrogen bonding. It can be said that the efficacy difference between the reference molecule and the natural molecule is due to the pi–pi interactions arising from the phenyl groups in atorvastatin, and the additional bonding energies of the groups capable of hydrogen bonding, may contribute in part to the differences in pharmacological properties.

Considering the genus *Salvia*, it has been reported that nearly 500 diterpene-derivative compounds have been isolated. In general, the effects of diterpenes on the inhibition of the mentioned enzyme have been reported in some studies. However, no study has yet been found in the literature on the HMG-CoA reductase inhibitory activity of abietane-type diterpenes. In our study, it was concluded that the abietane-type diterpenes, horminone, and horminone–7-acetoxyhorminone (1:1), were the leading compounds responsible for the HMG-CoA reductase inhibitory activity of *S. multicaulis* root extract. The results of our study may also form a basis for the use of some *Salvia* species in folk medicine against cardiovascular diseases, while horminone and 7-acetoxyhorminone may be considered as model molecules for designing new HMG-CoA reductase inhibitors.

## 4. Materials and Methods

### 4.1. General Experimental Procedures

NMR spectra (1D and 2D) were recorded on Agilent Premium Compact 600 MHz instruments, using TMS as an internal standard for chemical shifts. The Shimadzu 8040 LCMS-IT-TOF (LC-20 AD, SIL-20AC, DGU-20A3, CTO-20AC), Agilent GC-MS 7890 A, Shimadzu Scales (ATX224), rotary evaporator (Büchi L-100), and microplate reader (Eon Biotek-960) were used as the equipment. All chemicals used were of analytical grade.

### 4.2. Plant Material

The sample of *S. multicaulis* Vahl. was collected from the vicinity of Van Province (Turkey) in May 2014, and identified by Dr. Mehmet Firat (Faculty of Science, Herbarium of Van Yuzuncu Yil University, Van, Turkey). A voucher specimen was deposited in the Herbarium of Yuzuncu Yil under the code of M. Firat 30656 (VANF).

### 4.3. Extraction and Fractionation

The aerial parts and roots of *S. multicaulis* were dried in the shade and then powdered in a grinder. Both plant parts were sequentially macerated with petroleum ether, dichloromethane–acetone (1:1), and ethanol (95%), by occasional shaking at room temperature. After each filtration, the solvent was evaporated to dryness under a vacuum. Then, the enzyme inhibition assay was performed on each extract (Table 1). The root dichloromethane–acetone (1:1) extract was fractionated on a silica gel glass column (5 × 150 and 2 × 100 cm, respectively) using petroleum ether (40–60 °C), followed by a gradient elution using dichloromethane, acetone, methanol, and water, up to 50%, where 52 fractions in total were obtained. After combining phytochemically similar fractions according to thin layer chromatography (TLC) monitoring, 15 subfractions were obtained, which were immediately subjected to HMG-CoA reductase inhibition assays (Table 1). Following UV light checking, TLC plates were visualized by spraying them with cerium (IV) sulfate dissolved in 10% sulfuric acid. Silica gel, Sephadex LH-20 columns, and preparative TLC techniques were used to isolate compounds from the active fractions, which led to isolation of compounds **1**–**10**. The spectral data of the compounds is presented in supplementary material file.

### 4.4. Microtiter Assay for HMG-CoA Reductase Inhibition

The enzyme inhibition method developed by Wang et al. (2015) was applied. In the experimental procedure, 10 µL of the sample to be tested was first added to the microplate wells. Then, 10 µL of potassium phosphate buffer, prepared to contain EDTA (pH 7), dithiothrethiol (10 mmol/L), and bovine serum albumin (0.1 g/L) solution were added. Then, 20 µL of enzyme solution with a final concentration of 4 U/mL, and 40 µL of HMG-CoA (200 µM) solution were added and incubated at 37 °C for 5 min. Finally, 20 µL of NADPH was added and a measurement was taken at 340 nm in an ELISA microplate reader (Eon Biotek, Winooski, VT, USA). Atorvastatin was used as the reference drug and dimethylsulfoxide (DMSO) as a control [51].

### 4.5. Molecular Docking Experiments

The crystal structure of HMG-CoA reductase, coded 2Q1L, was downloaded from the Protein Data Bank (PDB)—the chains A and B were excluded from the structure and the rest of the system was managed for use in the simulations. All the hydrogen and missing atoms of the amino acids were added in the process of protein preparation with the preparation wizard of Maestro [52,53]. The protonation state of the system was set in a biological pH and its total energy was minimized using a force field method to remove any clashes between the atoms.

The 2D structures of the ligands were sketched and then their total energies were minimized in order to search for the most stable conformers. The docking method used here allowed both the ligand and the receptor to be flexible and optimized during the simulation. The idea behind this algorithm is to apply an induced fit docking method for exploring docking poses with the lowest binding free energies inside the active site of the receptor [54,55,56].

## 5. Conclusions

As a result, the HMG-CoA reductase inhibitory activity of various extracts and the isolated compounds of *S. multicaulis*, were determined for the first time in this study. 6,7-Dehydroyleanone (**1**), 12-demethylmulticaulin (**2**), ferruginol (**3**), and 12-hydroxy abieta-1, 3, 5 (**10**), 8, 11, 13-hexaene (**4**), β-sitosterol (**5**), horminone–7-acetoxyhorminone mixture (1:1) (**6**), 7-acetoxyhorminone (**7**), pisiferal (**8**), ursolic acid (**9**), and oleanolic acid (**10**) were isolated and their structures were elucidated. In particular, the HMG-CoA reductase inhibitory activity of 7-acetoxyhorminone was found to be noteworthy. Therefore, it has been determined that *S. multicaulis* and 7-acetoxyhorminone are active against HMG-CoA reductase and may have the potential to be used in drug research. The present work is the first study revealing that the isolated abietane-type diterpenes, horminone–7-acetoxyhorminone mixture (1:1) (**6**) and 7-acetoxyhorminone (**7**), strongly inhibit HMG-CoA reductase.

## Figures and Tables

**Table 1:** HMG-CoA reductase inhibitory activity of *S. multicaulis* extracts and fractions.

Extracts and Fractions	HMG-CoA Reductase Inhibition (Inhibition % ± SD ^a^ at 100 µg/mL)
Aerial part-petroleum ether	NA ^b^
Root-petroleum ether	NA
Aerial part-ethanol	57.16 ± 0.24
Root-ethanol	60.26 ± 0.19
Aerial part-dichloromethane–acetone (1:1)	55.21 ± 0.48
Root-dichloromethane–acetone (1:1)	71.97 ± 0.36
M-1: Fr. 1–4	NA
M-2: Fr. 5–6	16.30 ± 0.33
M-3: Fr. 7–9	**50.17 ± 1.24 ^c^**
M-4: Fr. 10–13	3.50 ± 2.61
M-5: Fr. 14	15.22 ± 0.08
M-6: Fr. 15–17	**54.81 ± 0.80**
M-7: Fr. 18–20	**52.83 ± 0.31**
M-8: Fr. 21–22	**57.39 ± 0.06**
M-9: Fr. 23–24	32.12 ± 0.11
M-10: Fr. 25–26	**56.45 ± 0.14**
M-11: Fr. 27–32	29.66 ± 2.57
M-12: Fr. 33–35	28.20 ± 0.20
M-13: Fr. 36–40	NA
M-14: Fr. 41–48	NA
M-15: Fr. 49–52	NA
Atorvastatin	91.06 ± 0.46

^a^ Standard deviation: Values expressed are the means ± SD of three parallel measurements and the values were calculated according to a negative control, ^b^ NA: Not active, ^c^ Fractions with inhibition over 50% were bolded.

**Table 2:** HMG-CoA reductase inhibitory activity of the isolated compounds.

No	Compounds	HMG-CoA Reductase Inhibition (Inhibition % ± SD ^a^ at 100 µg/mL)
**1**	6,7-Dehydroyleanone	28.50 ± 0.13
**2**	12-Demethylmulticaulin	35.29 ± 0.05
**3**	Ferruginol	18.11 ± 0.10
**4**	12-Hydroxy abieta-1, 3, 5(10), 8, 11, 13-hexaene	2.26 ± 0.21
**5**	β-Sitosterol	12.52 ± 0.14
**6**	Horminone–7-acetoxyhorminone	**76.26 ± 0.14 ^c^** (IC_50_ = 52.3 ± 0.78 µg/mL)
**7**	7-Acetoxyhorminone	**84.15 ± 0.10** (IC_50_ = 63.6 ± 1.21 µg/mL)
**8**	Pisiferal	NA ^b^
**9**	Ursolic acid	43.32 ± 0.11
**10**	Oleanolic acid	25.00 ± 0.14
Atorvastatin		97.16 ± 0.01 (IC_50_ = 3.0 ± 0.36 µg/mL)

^a^ Standard deviation: Values expressed are the means ± SD of three parallel measurements and the values were calculated according to a negative control, ^b^ NA: Not active, ^c^ Compounds with inhibition over 50% were bolded.

## Data Availability

Data is contained within the article and supplementary material.

## Supplementary Materials

The following supporting information can be downloaded at: https://www.mdpi.com/article/10.3390/ph15020198/s1: Figures S1–S53. Figure S1. ^1^H NMR spectrum of 1 in CD_3_OD (600 MHz), Figure S2. ^13^C NMR (APT) spectrum of 1 in CD_3_OD (600 MHz), Figure S3. HMBC spectrum of 1 in CD_3_OD (600 MHz), Figure S4. HMQC spectrum of 1 in CD_3_OD (600 MHz), Figure S5. GC-MS spectrum of 1, Figure S6. LC-MS-IT-TOF spectrum of 1, Figure S7. ^1^H NMR spectrum of 2 in CD_3_OD (600 MHz), Figure S8. ^13^C NMR (APT) spectrum of 2 in CD_3_OD (600 MHz), Figure S9. GC-MS spectrum of 2, Figure S10. LC-MS-IT-TOF spectrum of 2, Figure S11. ^1^H NMR spectrum of 3 in CD_3_OD (600 MHz), Figure S12. ^13^C NMR (APT) spectrum of 3 in CD_3_OD (600 MHz), Figure S13. HMBC spectrum of 3 in CD_3_OD (600 MHz), Figure S14. HMQC spectrum of 3 in CD_3_OD (600 MHz), Figure S15. GC-MS spectrum of 3, Figure S16. LC-MS-IT-TOF spectrum of 3, Figure S17. ^1^H NMR spectrum of 4 in CD_3_OD (600 MHz), Figure S18. ^13^C NMR (APT) spectrum of 4 in CD_3_OD (600 MHz), Figure S19. HMBC spectrum of 4 in CD_3_OD (600 MHz), Figure S20. HMQC spectrum of 4 in CD_3_OD (600 MHz), Figure S21. GC-MS spectrum of 4, Figure S22. ^1^H NMR spectrum of 5 in CD_3_OD (600 MHz), Figure S23. ^13^C NMR (APT) spectrum of 5 in CD_3_OD (600 MHz), Figure S24. HMBC spectrum of 5 in CD_3_OD (600 MHz), Figure S25. HMQC spectrum of 5 in CD_3_OD (600 MHz), Figure S26. GC-MS spectrum of 5, Figure S27. LC-MS-IT-TOF spectrum of 5, Figure S28. ^1^H NMR spectrum of 6 in CD_3_OD (600 MHz), Figure S29. ^13^C NMR (APT) spectrum of 6 in CD_3_OD (600 MHz), Figure S30. HMBC spectrum of 6 in CD_3_OD (600 MHz), Figure S31. HMQC spectrum of 6 in CD_3_OD (600 MHz), Figure S32. GC-MS spectrum of 6, Figure S33. ^1^H NMR spectrum of 7 in CD_3_OD (600 MHz), Figure S34. ^13^C NMR (APT) spectrum of 7 in CD_3_OD (600 MHz), Figure S35. HMBC spectrum of 7 in CD_3_OD (600 MHz), Figure S36. HMQC spectrum of 7 in CD_3_OD (600 MHz), Figure S37. GC-MS spectrum of 7, Figure S38. ^1^H NMR spectrum of 8 in CD_3_OD (600 MHz), Figure S39. ^13^C NMR (APT) spectrum of 8 in CD_3_OD (600 MHz), Figure S40. HMBC spectrum of 8 in CD_3_OD (600 MHz), Figure S41. HMQC spectrum of 8 in CD_3_OD (600 MHz), Figure S42. GC-MS spectrum of 8, Figure S43. LC-MS-IT-TOF spectrum of 8, Figure S44. ^1^H NMR spectrum of 9 in CD_3_OD (600 MHz), Figure S45. ^13^C NMR spectrum of 9 in CD_3_OD (600 MHz), Figure S46. HMBC spectrum of 9 in CD_3_OD (600 MHz), Figure S47. HMQC spectrum of 9 in CD_3_OD (600 MHz), Figure S48. LC-MS-IT-TOF spectrum of 9, Figure S49. ^1^H NMR spectrum of 10 in CD_3_OD (600 MHz), Figure S50. ^13^C NMR (APT) spectrum of 10 in CD_3_OD (600 MHz), Figure S51. HMBC spectrum of 10 in CD_3_OD (600 MHz), Figure S52. HMQC spectrum of 10 in CD_3_OD (600 MHz), Figure S53. LC-MS-IT-TOF spectrum of 10.
