# Supplementary material for: 7-Acetoxyhorminone from Salvia multicaulis Vahl. as Promising Inhibitor of 3-Hydroxy-3-methylglutaryl Coenzyme A (HMG-CoA) Reductase
Source: Pharmaceuticals (Basel). 2022 Feb 4;15(2):198. doi: 10.3390/ph15020198 (PMC8880194; doi:10.3390/ph15020198)
Supplement: Supplementary file 1 [file pharmaceuticals-15-00198-s001.zip › pharmaceuticals-1551664-supplementary.pdf]

7-Acetoxyhorminone Isolated from *Salvia multicaulis* Vahl. as Promising Inhibitor of 3-Hydroxy-3-Methylglutaryl Coenzyme A (HMG-CoA) Reductase

Serkan Yiğitkan[1,6], Abdulselam Ertaş[2,3], Ramin Ekhteiari Salmas[4], Mehmet Fırat[5], Ilkay Erdogan Orhan[6]

[1]*Department of Pharmaceutical Botany, Faculty of Pharmacy, Dicle University, Turkey*

[2]*Department of Analytical Chemistry, Faculty of Pharmacy, Dicle University, Turkey*

[3]*Dicle University Cancer Research Center, Dicle University, Turkey*

[4]*Department of Chemistry, Britannia House, King's College London, SE1 1DB, UK*

[5]*Department of Biology, Faculty of Education, Van Yuzuncu Yil University, Turkey*

[6] *Department of Pharmacognosy, Faculty of Pharmacy, Gazi University, 06330 Ankara, Turkey*

Table of Contents **Page**

**Figure S1.** ^{1}H NMR spectrum of **1** in CD_3OD (600 MHz)	3
**Figure S2.** ^{13}C NMR (APT) spectrum of **1** in CD_3OD (600 MHz)	4
**Figure S3.** HMBC spectrum of **1** in CD_3OD (600 MHz)	5
**Figure S4.** HMQC spectrum of **1** in CD_3OD (600 MHz)	6
**Figure S5.** GC-MS spectrum of **1**	7
**Figure S6.** LC-MS-IT-TOF spectrum of **1**	8
**Figure S7.** ^{1}H NMR spectrum of **2** in CD_3OD (600 MHz)	9
**Figure S8.** ^{13}C NMR (APT) spectrum of **2** in CD_3OD (600 MHz)	10
**Figure S9.** GC-MS spectrum of **2**	11
**Figure S10.** LC-MS-IT-TOF spectrum of **2**	12
**Figure S11.** ^{1}H NMR spectrum of **3** in CD_3OD (600 MHz)	13
**Figure S12.** ^{13}C NMR (APT) spectrum of **3** in CD_3OD (600 MHz)	14
**Figure S13.** HMBC spectrum of **3** in CD_3OD (600 MHz)	15
**Figure S14.** HMQC spectrum of **3** in CD_3OD (600 MHz)	16
**Figure S15.** GC-MS spectrum of **3**	17
**Figure S16.** LC-MS-IT-TOF spectrum of **3**	18
**Figure S17.** ^{1}H NMR spectrum of **4** in CD3OD (600 MHz)	19
**Figure S18.** ^{13}C NMR (APT) spectrum of **4** in CD3OD (600 MHz)	20
**Figure S19.** HMBC spectrum of **4** in CD_3OD (600 MHz)	21
**Figure S20.** HMQC spectrum of **4** in CD_3OD (600 MHz)	22
**Figure S21.** GC-MS spectrum of **4**	23
**Figure S22.** ^{1}H NMR spectrum of **5** in CD_3OD (600 MHz)	24
**Figure S23.** ^{13}C NMR (APT) spectrum of **5** in CD_3OD (600 MHz)	25
**Figure S24.** HMBC spectrum of **5** in CD_3OD (600 MHz)	26

Figure S25. HMQC spectrum of **5** in CD_3OD (600 MHz) 27
Figure S26. GC-MS spectrum of **5** 28
Figure S27. LC-MS-IT-TOF spectrum of **5** 29
Figure S28. ^1H NMR spectrum of **6** in CD3OD (600 MHz) 30
Figure S29. ^{13}C NMR (APT) spectrum of **6** in CD3OD (600 MHz) 31
Figure S30. HMBC spectrum of **6** in CD3OD (600 MHz) 32
Figure S31. HMQC spectrum of **6** in CD_3OD (600 MHz) 33
Figure S32. GC-MS spectrum of **6** 34
Figure S33. ^1H NMR spectrum of **7** in CD3OD (600 MHz) 35
Figure S34. ^{13}C NMR (APT) spectrum of **7** in CD3OD (600 MHz) 36
Figure S35. HMBC spectrum of **7** in CD3OD (600 MHz) 37
Figure S36. HMQC spectrum of **7** in CD_3OD (600 MHz) 38
Figure S37. GC-MS spectrum of **7** 39
Figure S38. ^1H NMR spectrum of **8** in CD_3OD (600 MHz) 40
Figure S39. ^{13}C NMR (APT) spectrum of **8** in CD_3OD (600 MHz) 41
Figure S40. HMBC spectrum of **8** in CD_3OD (600 MHz) 42
Figure S41. HMQC spectrum of **8** in CD_3OD (600 MHz) 43
Figure S42. GC-MS spectrum of **8** 44
Figure S43. LC-MS-IT-TOF spectrum of **8** 45
Figure S44. ^1H NMR spectrum of **9** in CD_3OD (600 MHz) 46
Figure S45. ^{13}C NMR spectrum of **9** in CD_3OD (600 MHz) 47
Figure S46. HMBC spectrum of **9** in CD_3OD (600 MHz) 48
Figure S47. HMQC spectrum of **9** in CD_3OD (600 MHz) 49
Figure S48. LC-MS-IT-TOF spectrum of **9** 50
Figure S49. ^1H NMR spectrum of **10** in CD_3OD (600 MHz) 51
Figure S50. ^{13}C NMR (APT) spectrum of **10** in CD_3OD (600 MHz) 52
Figure S51. HMBC spectrum of **10** in CD_3OD (600 MHz) 53
Figure S52. HMQC spectrum of **10** in CD_3OD (600 MHz) 54
Figure S53. LC-MS-IT-TOF spectrum of **10** 55

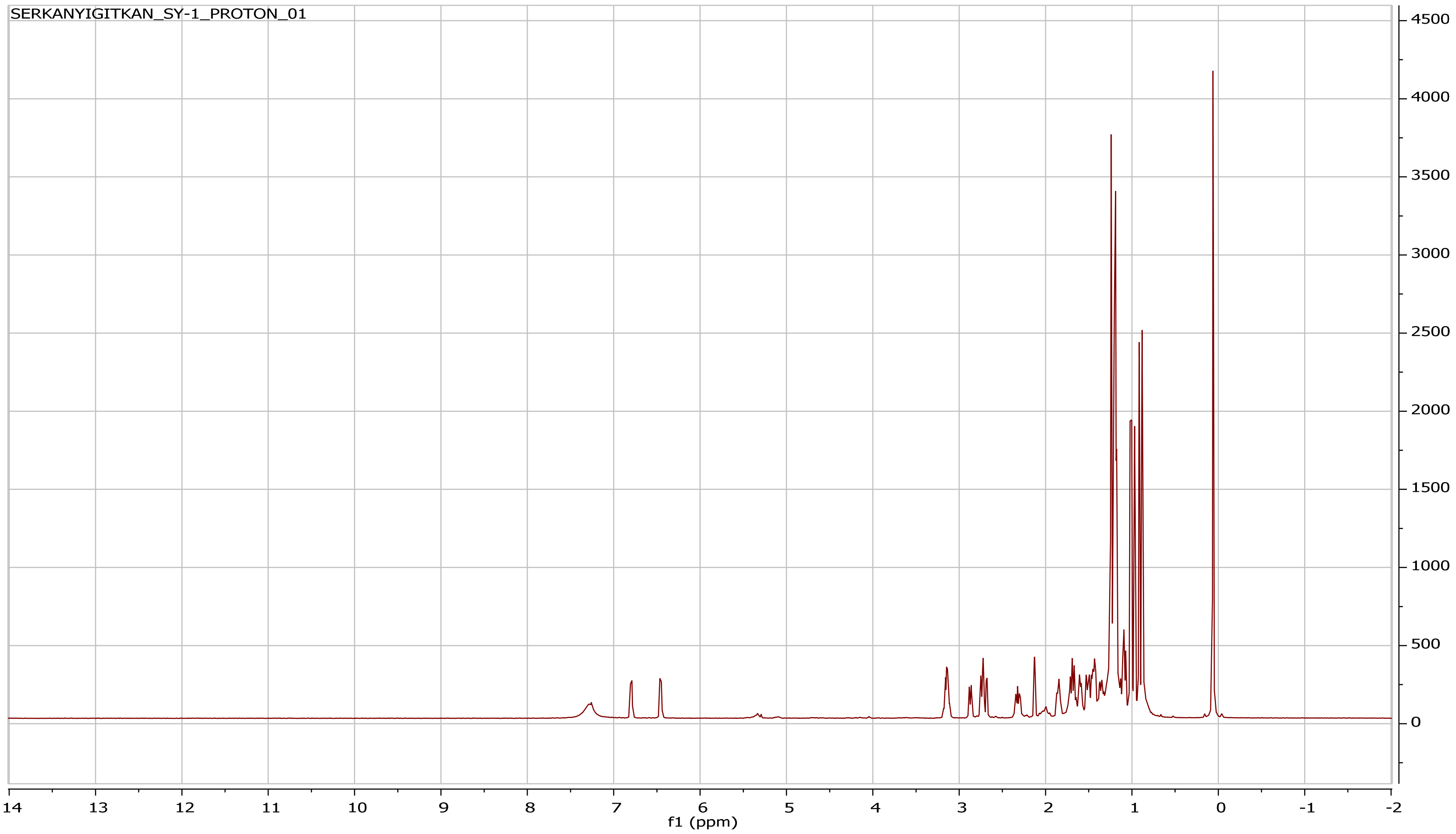

Figure S1. ^{1}H NMR spectrum of **1** in CD_3OD (600 MHz)

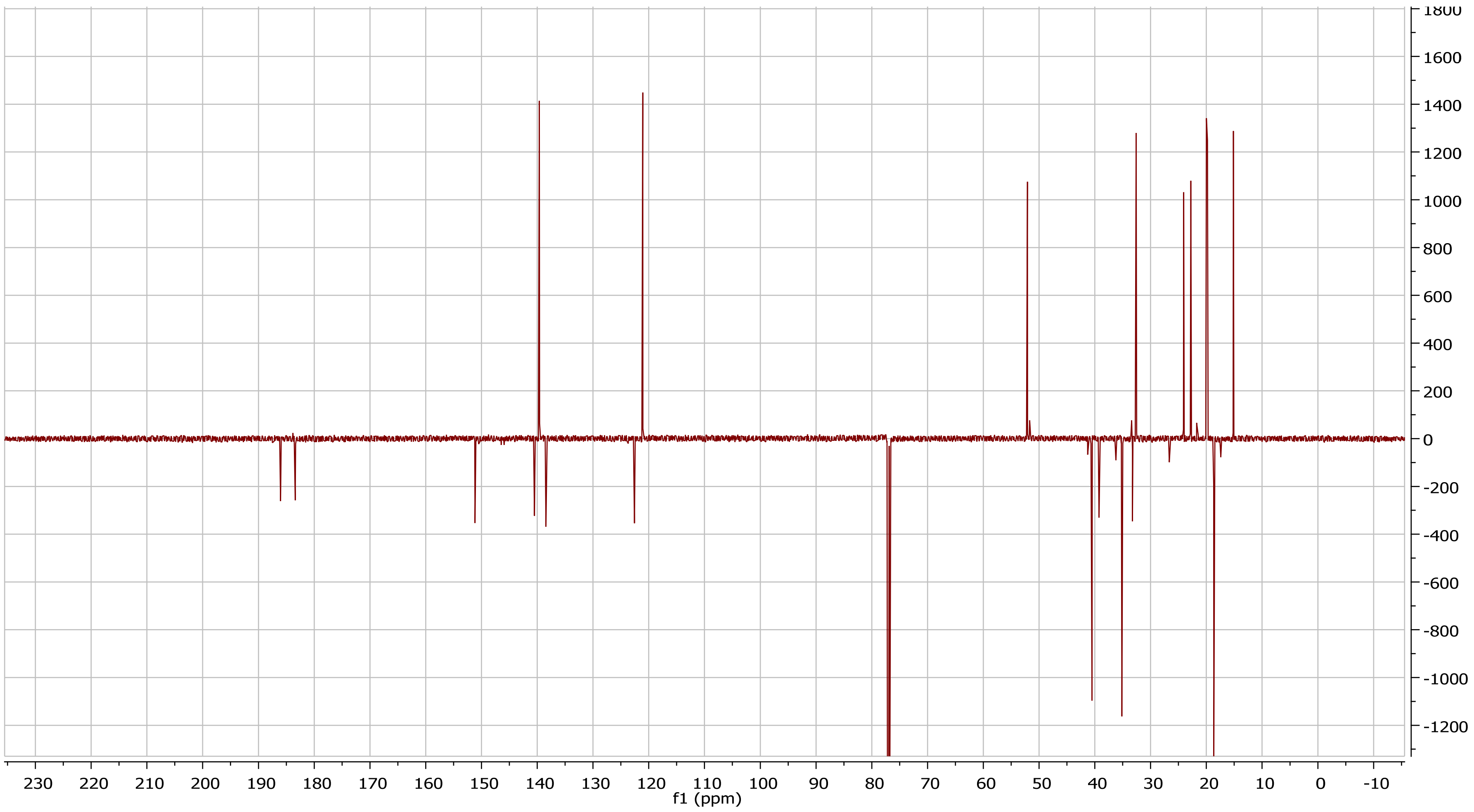

Figure S2. ^{13}C NMR (APT) spectrum of **1** in CD_3OD (600 MHz)

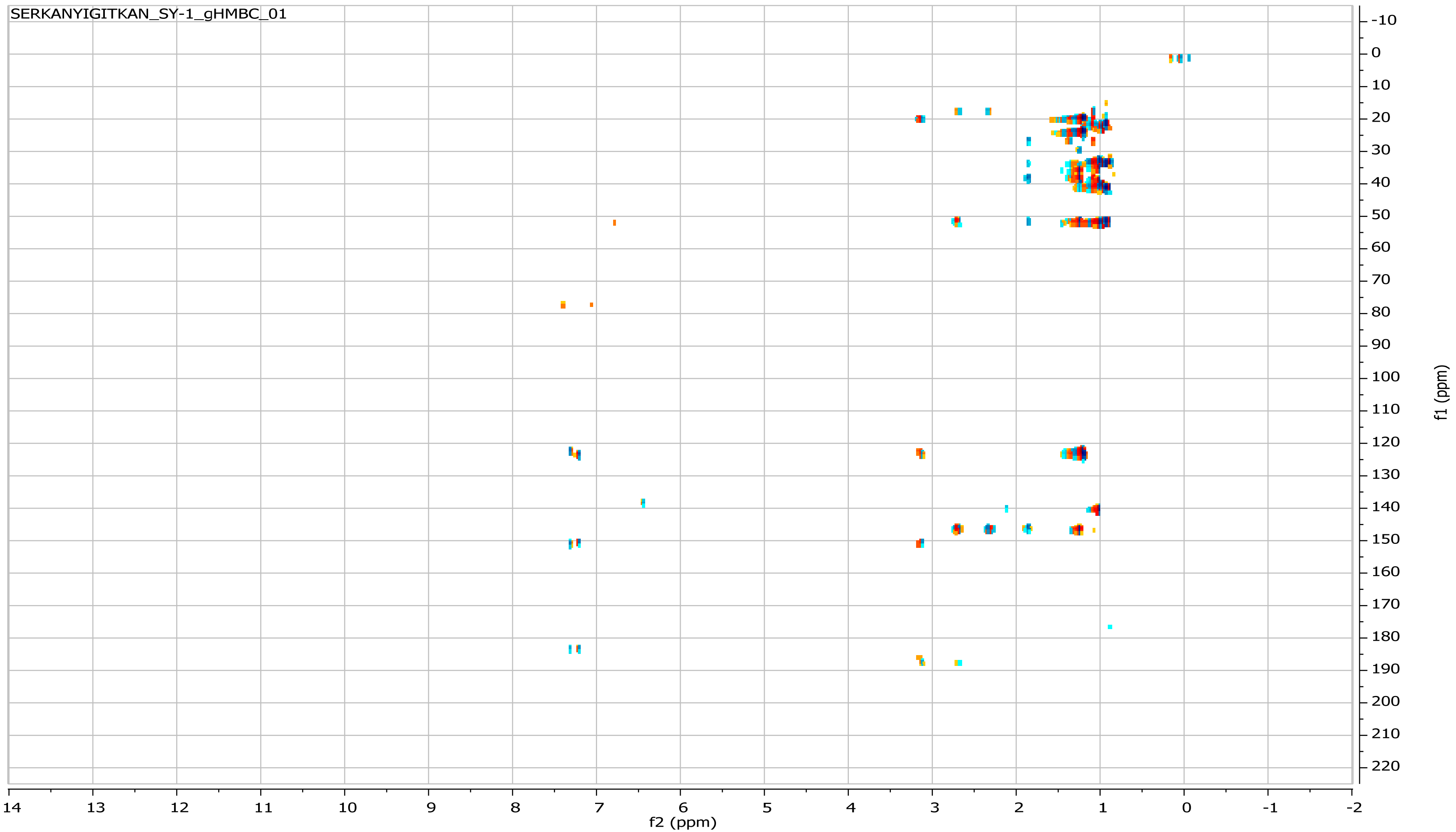

Figure S3. HMBC spectrum of **1** in CD_3OD (600 MHz)

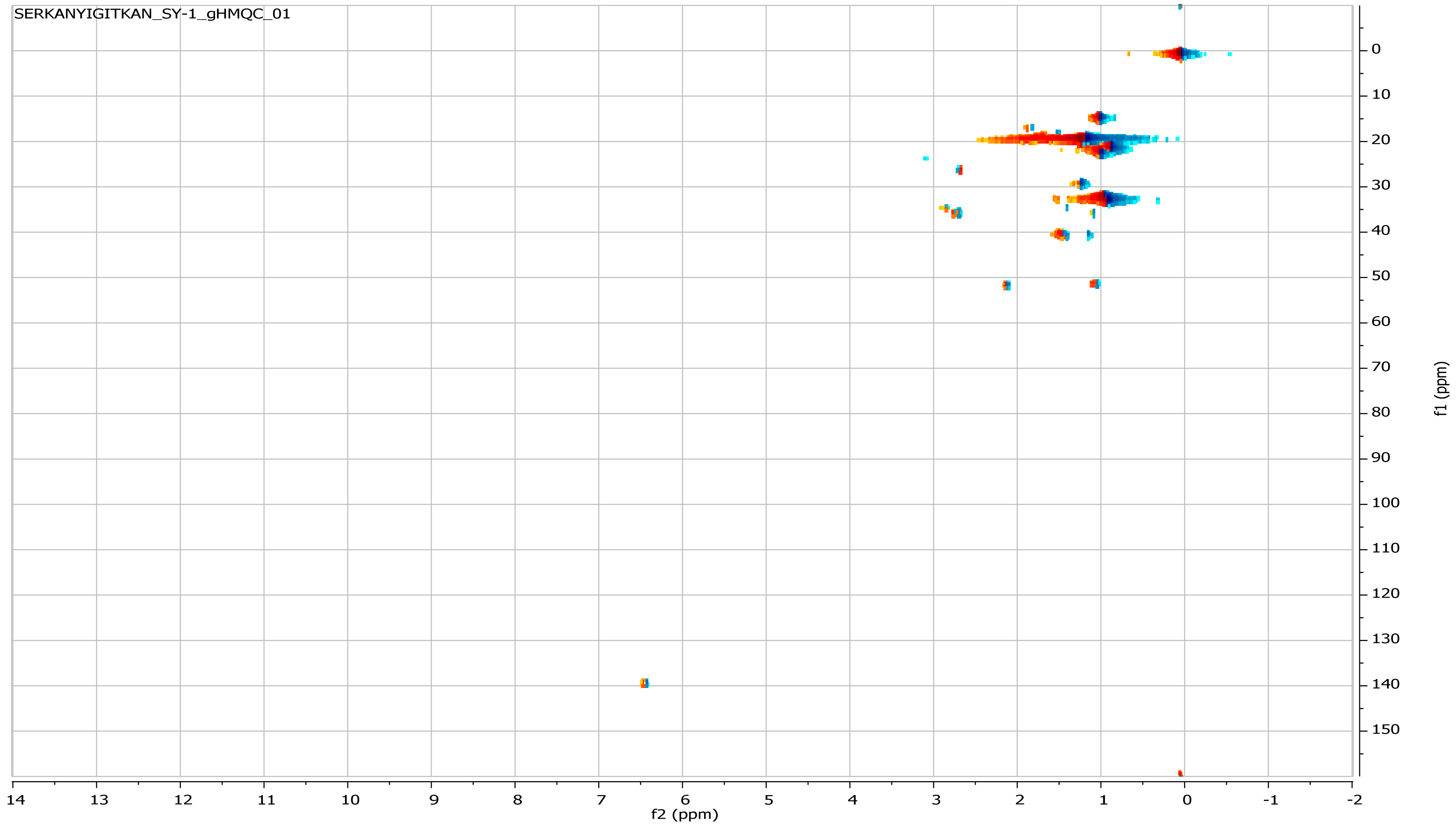

Figure S4. HMQC spectrum of **1** in CD_3OD (600 MHz)

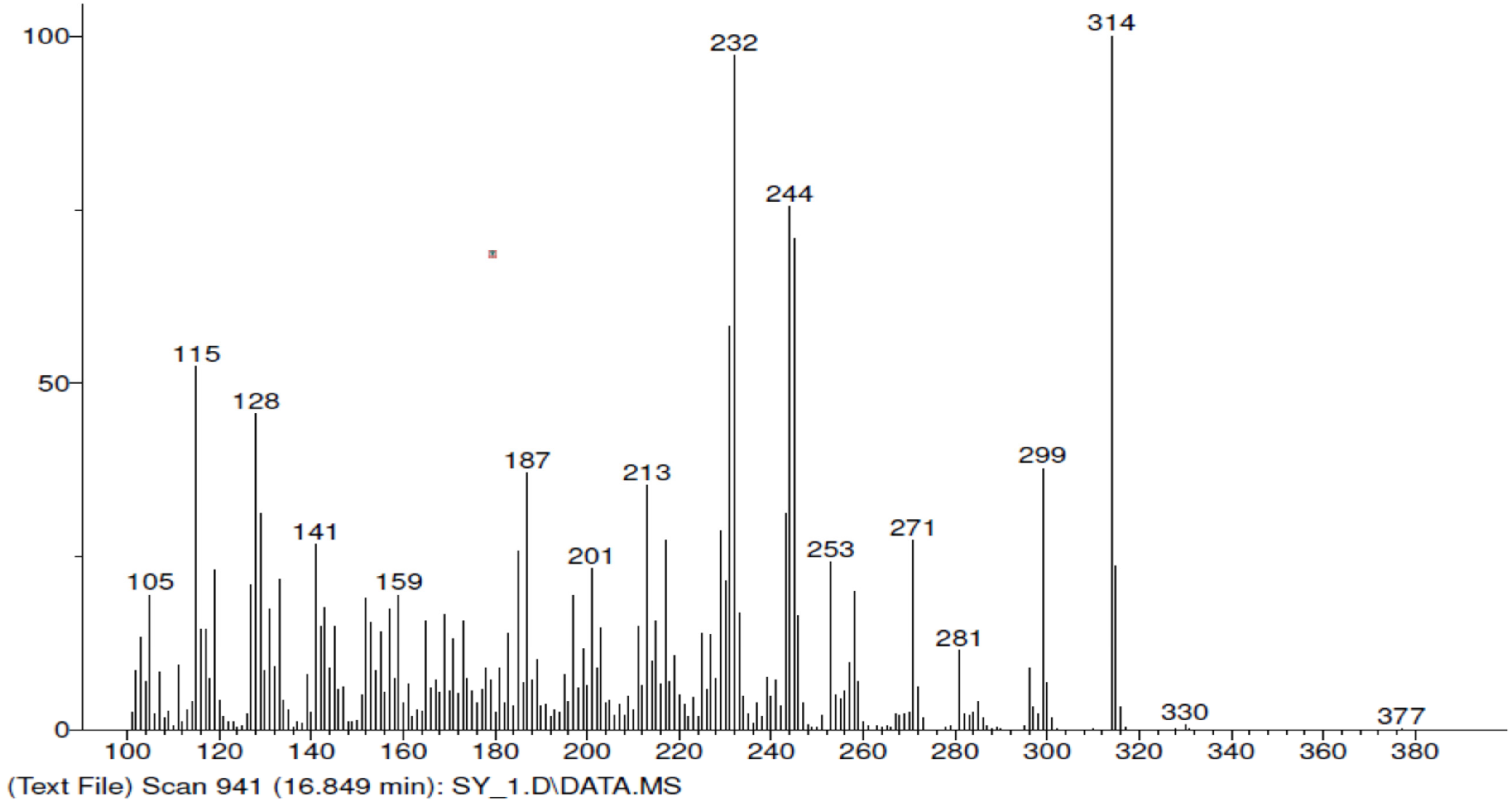

Figure S5. GC-MS spectrum of **1**

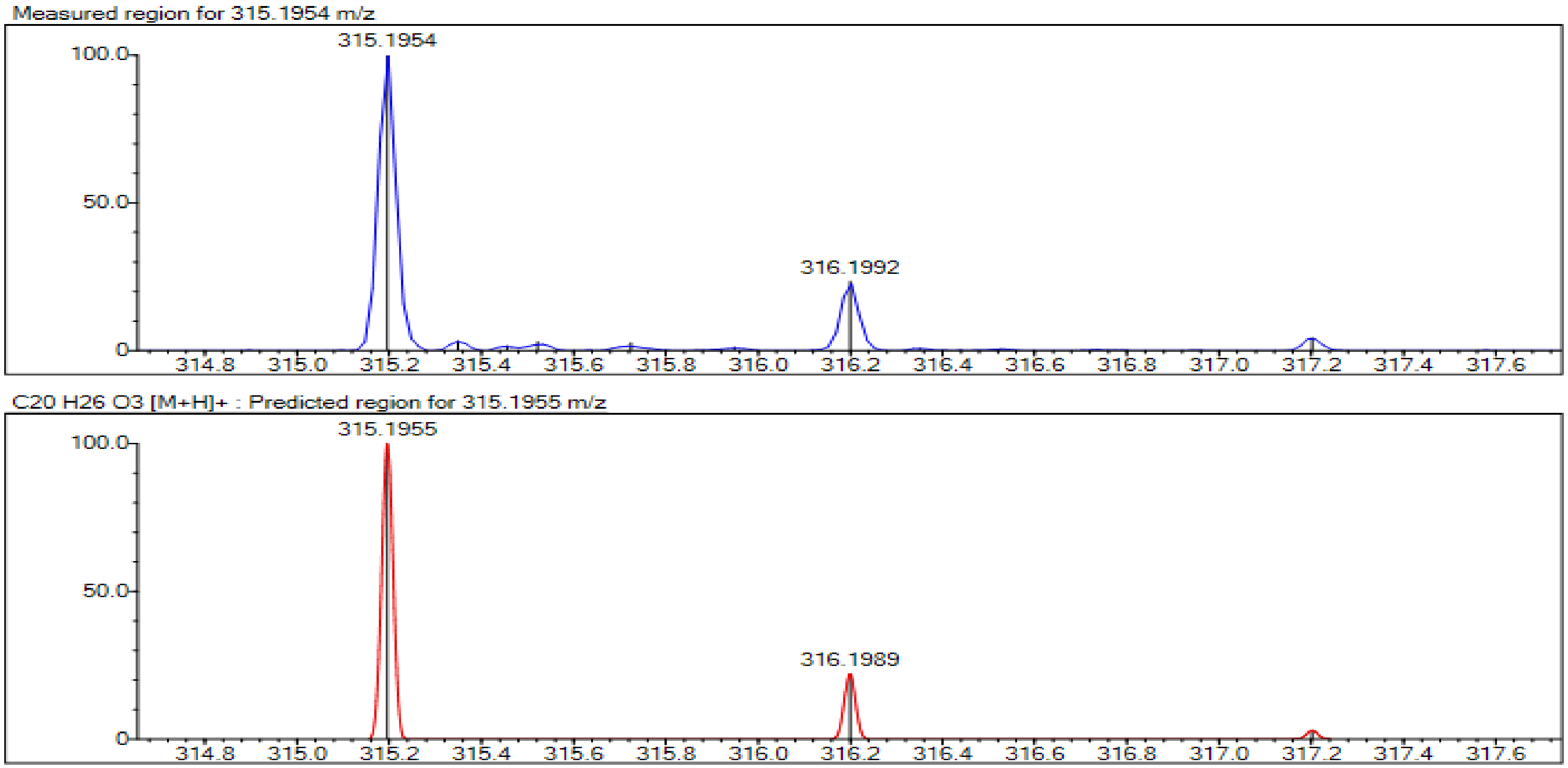

Rank	Score	Formula (M)	Ion	Meas. m/z	Pred. m/z	Df. (mDa)	Df. (ppm)	Iso	DBE
1	90.31	C20 H26 O3	[M+H]+	315.1954	315.1955	-0.1	-0.32	90.31	8.0

Figure S6. LC-MS-IT-TOF spectrum of **1**

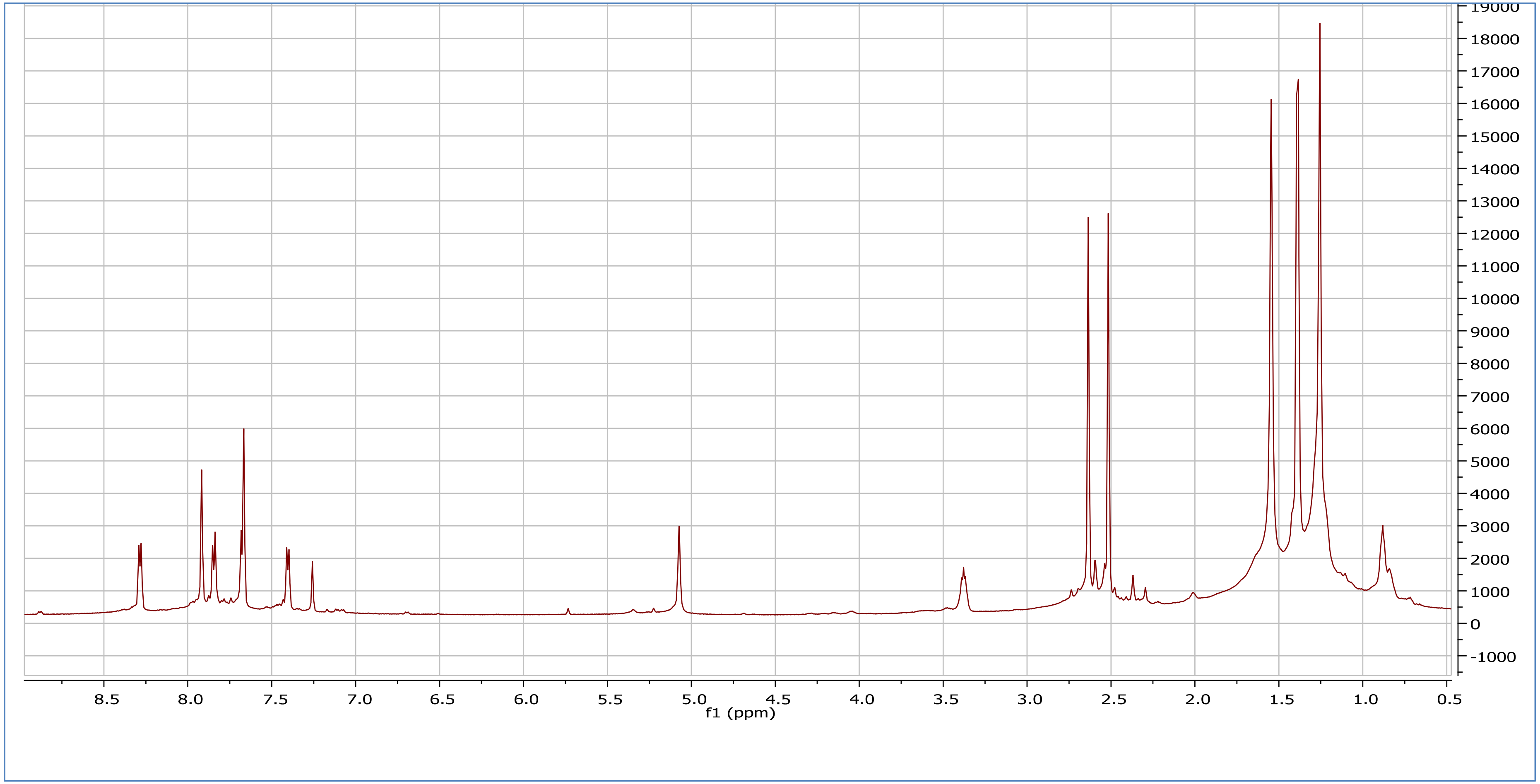

Figure S7. ^{1}H NMR spectrum of **2** in CD_3OD (600 MHz)

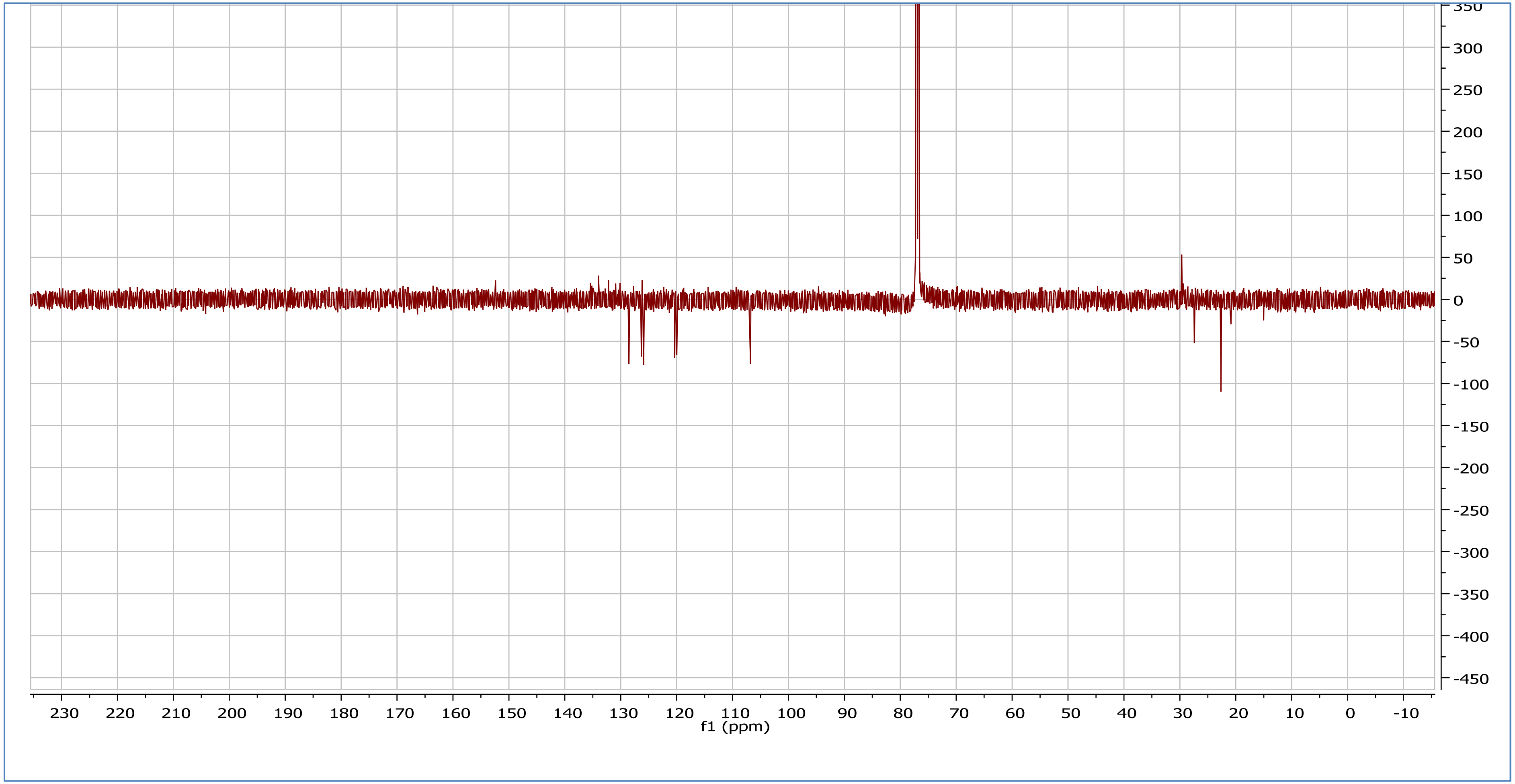

Figure S8. ^{13}C NMR (APT) spectrum of **2** in CD_3OD (600 MHz)

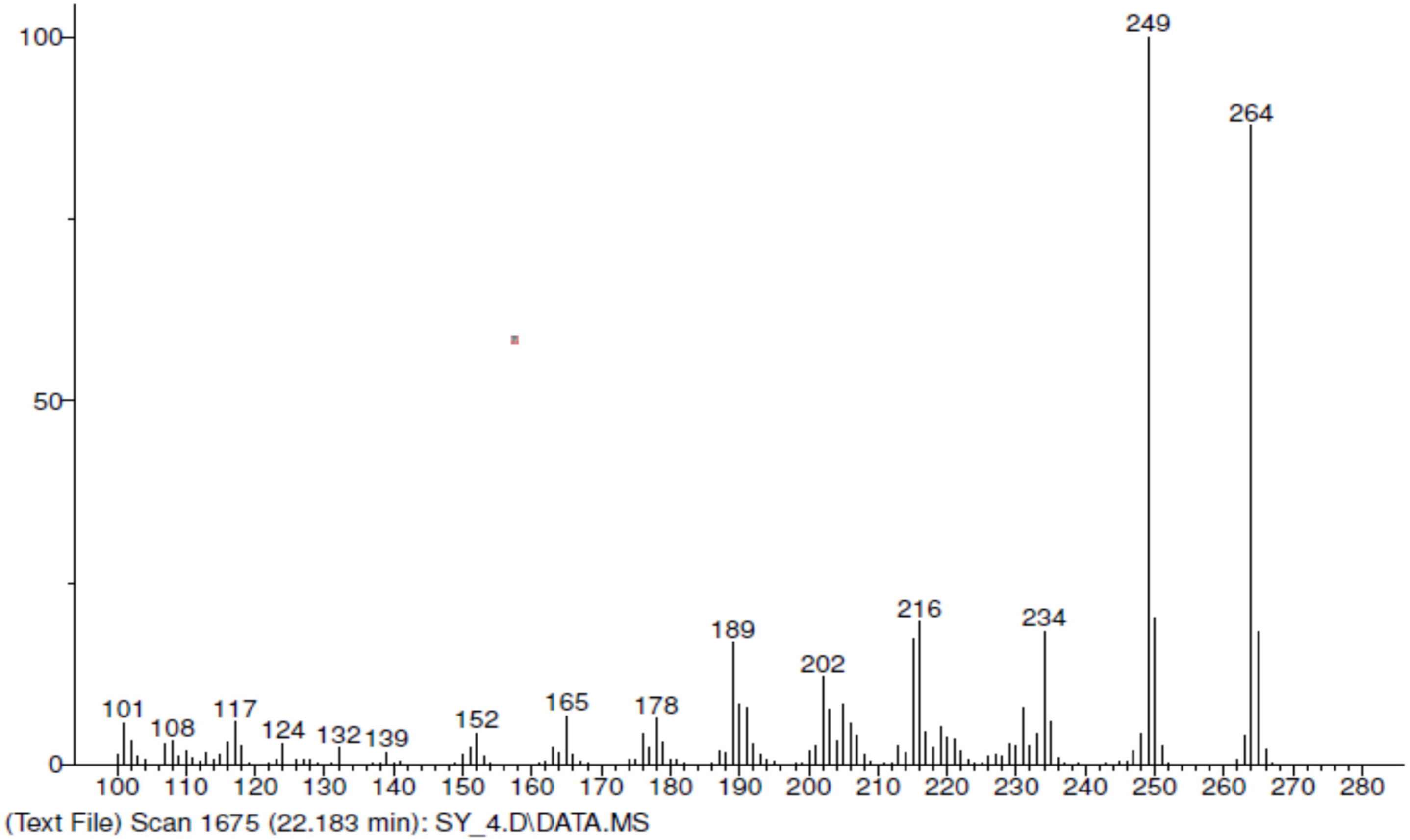

Figure S9. GC-MS spectrum of **2**

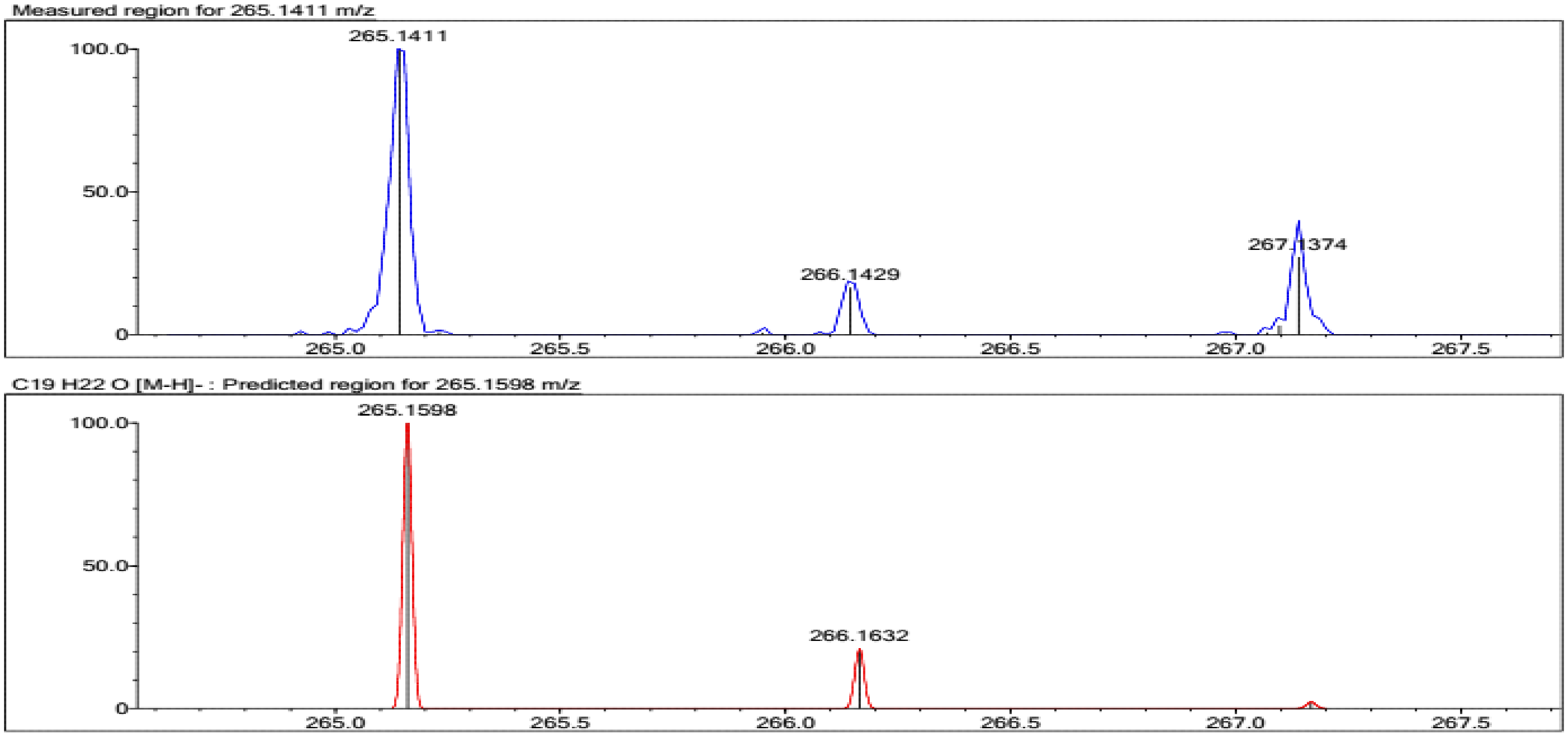

Rank	Score	Formula (M)	Ion	Meas. m/z	Pred. m/z	Df. (mDa)	Df. (ppm)	Iso	DBE
7	0.00	C19 H22 O	[M-H]-	265.1411	265.1598	-18.7	-70.53	7.41	9.0

Figure S10. LC-MS-IT-TOF spectrum of **2**

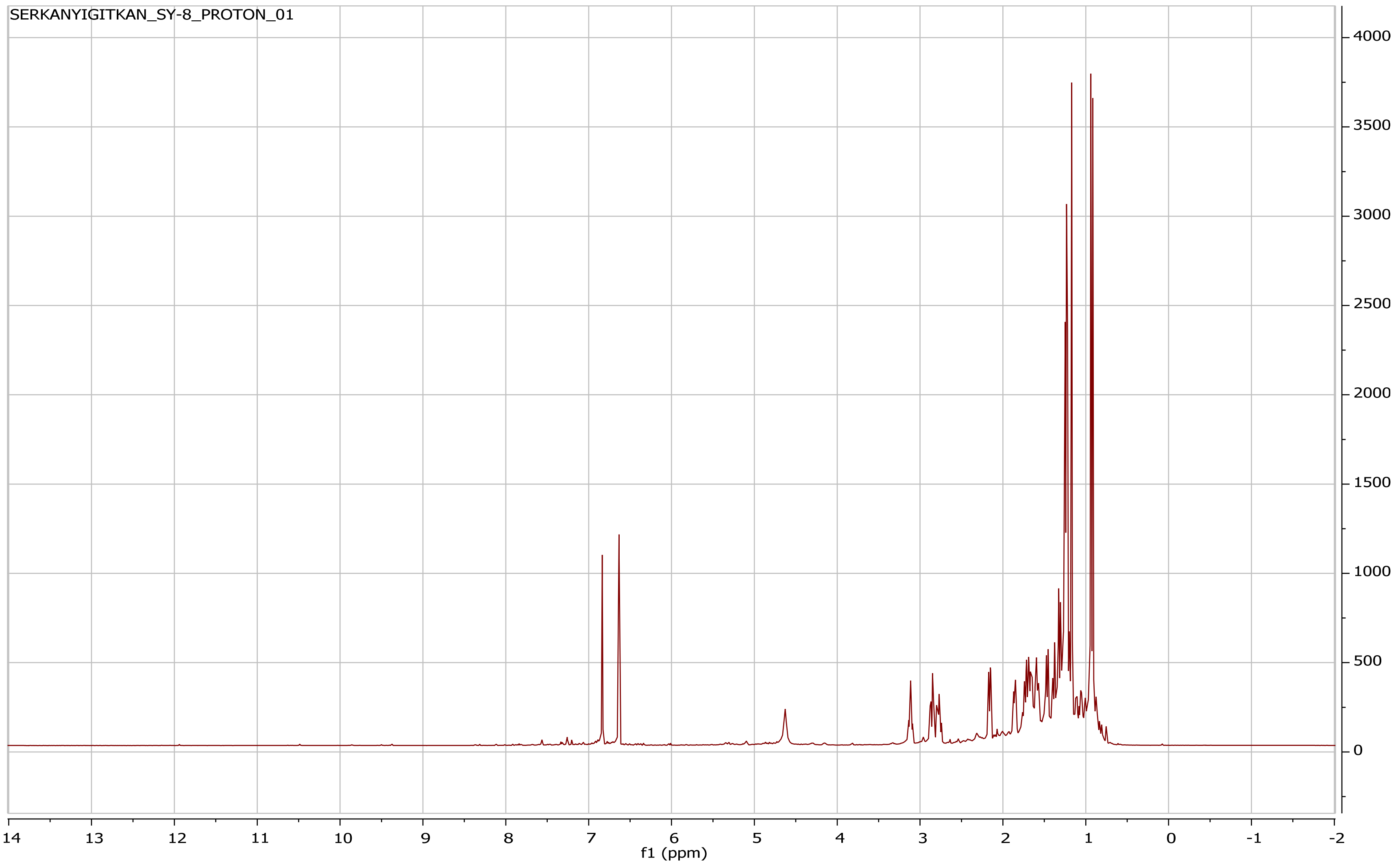

Figure S11. ^{1}H NMR spectrum of **3** in CD_3OD (600 MHz)

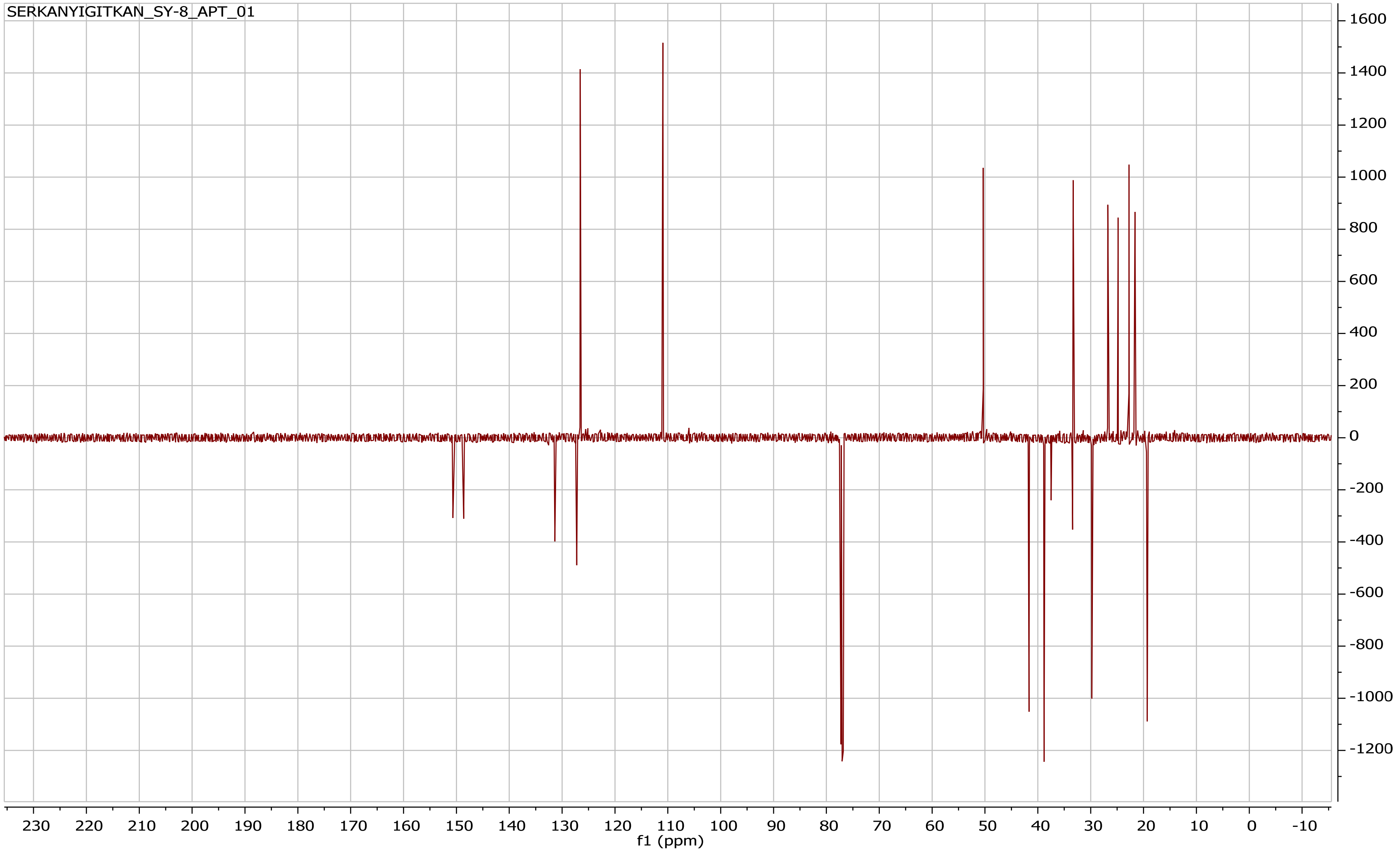

Figure S12. ^{13}C NMR (APT) spectrum of **3** in CD_3OD (600 MHz)

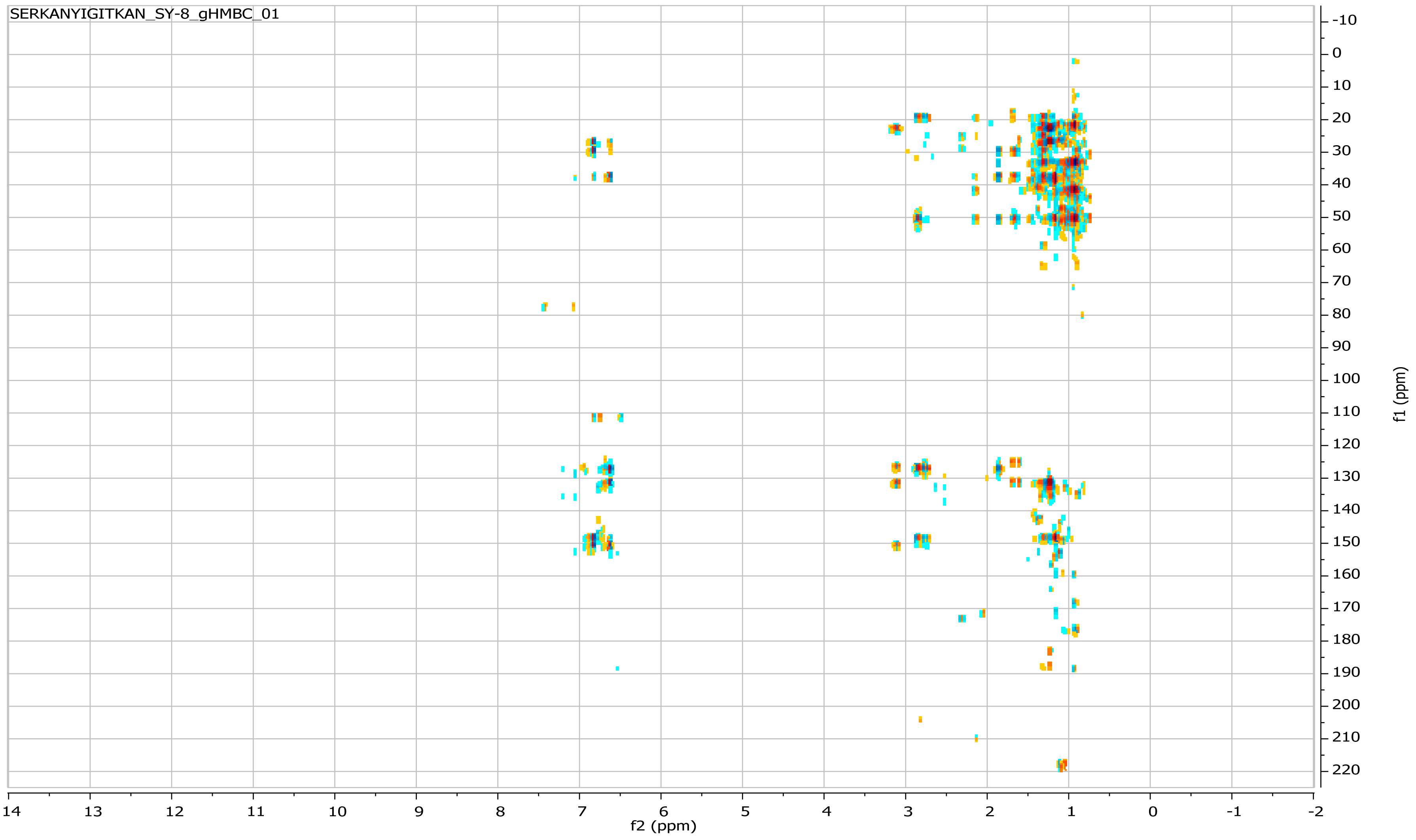

Figure S13. HMBC spectrum of **3** in CD_3OD (600 MHz)

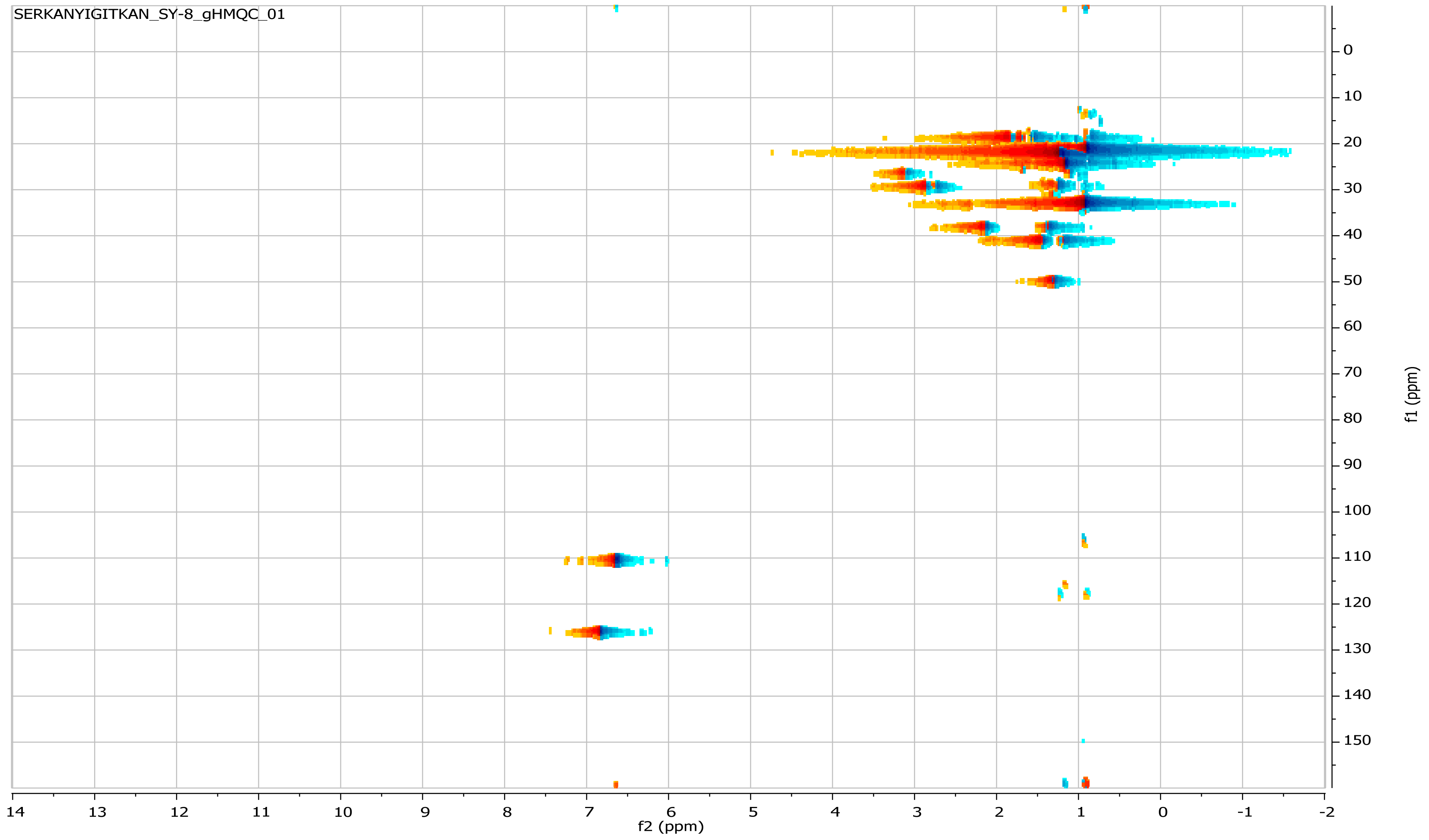

Figure S14. HMQC spectrum of **3** in CD_3OD (600 MHz)

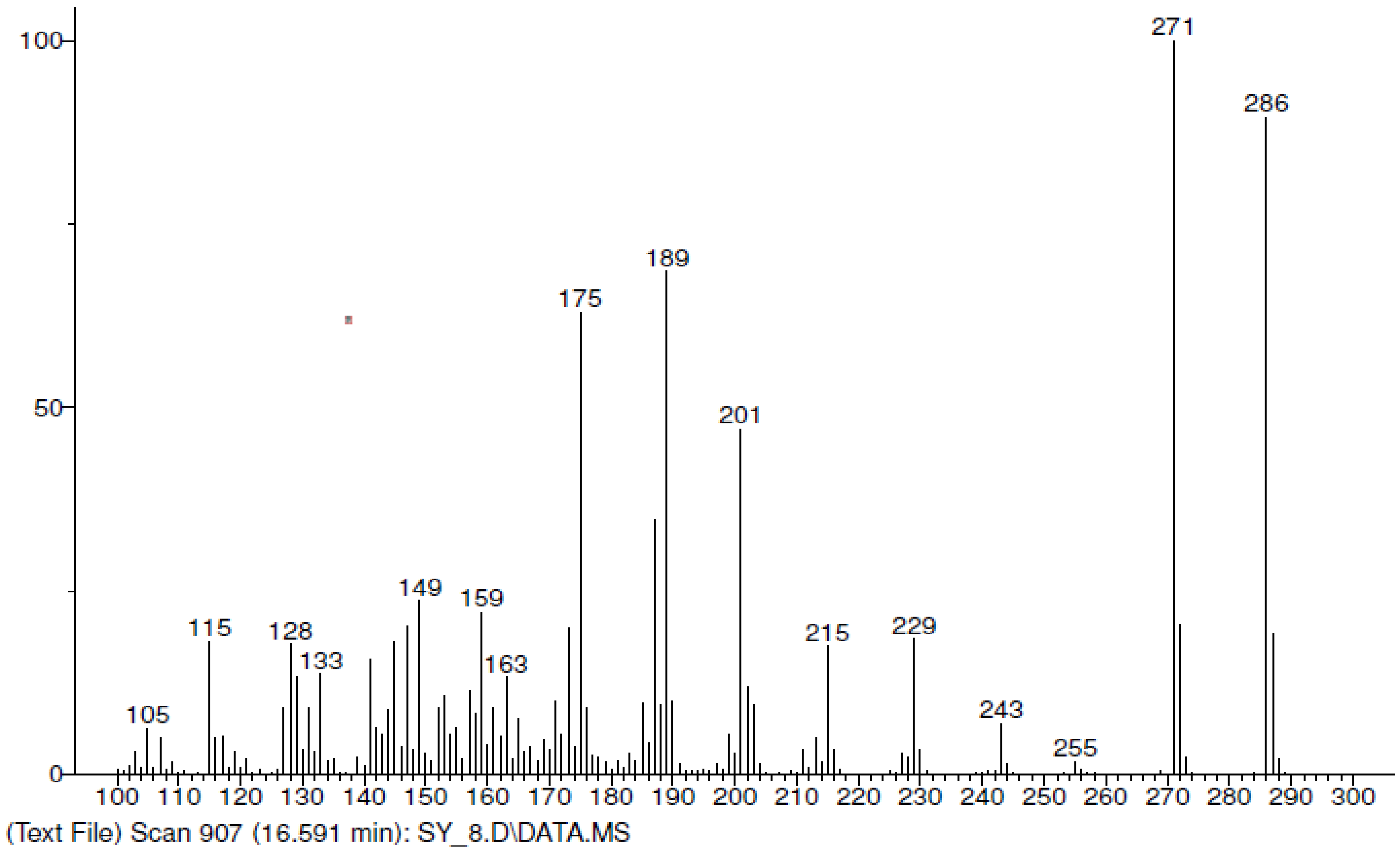

Figure S15. GC-MS spectrum of **3**

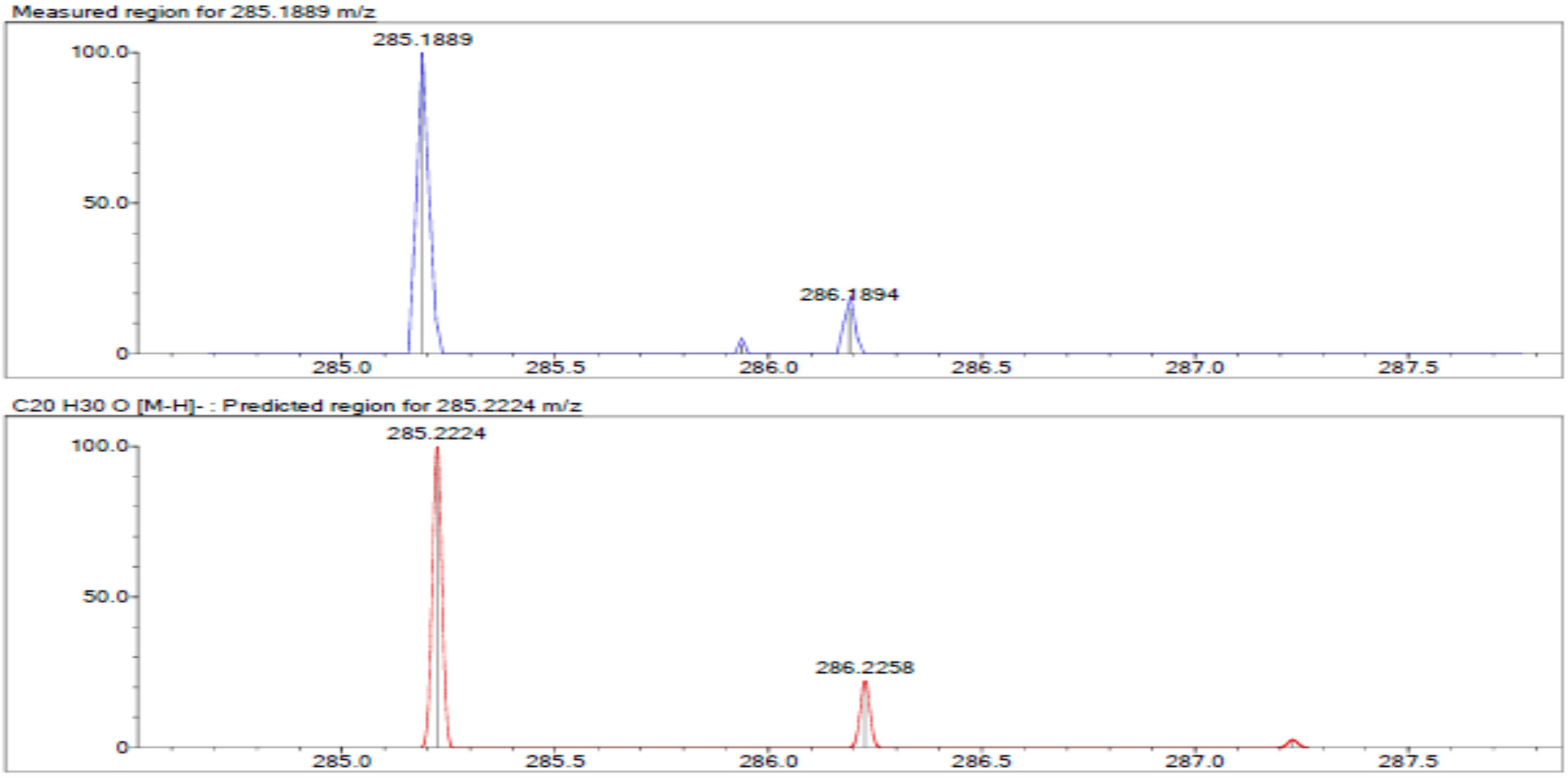

Rank	Score	Formula (M)	Ion	Meas. m/z	Pred. m/z	Df. (mDa)	Df. (ppm)	Iso	DBE
12	0.00	C20 H30 O	[M-H]-	285.1889	285.2224	-33.5	-117.47	0.00	6.0

Figure S16. LC-MS-IT-TOF spectrum of **3**

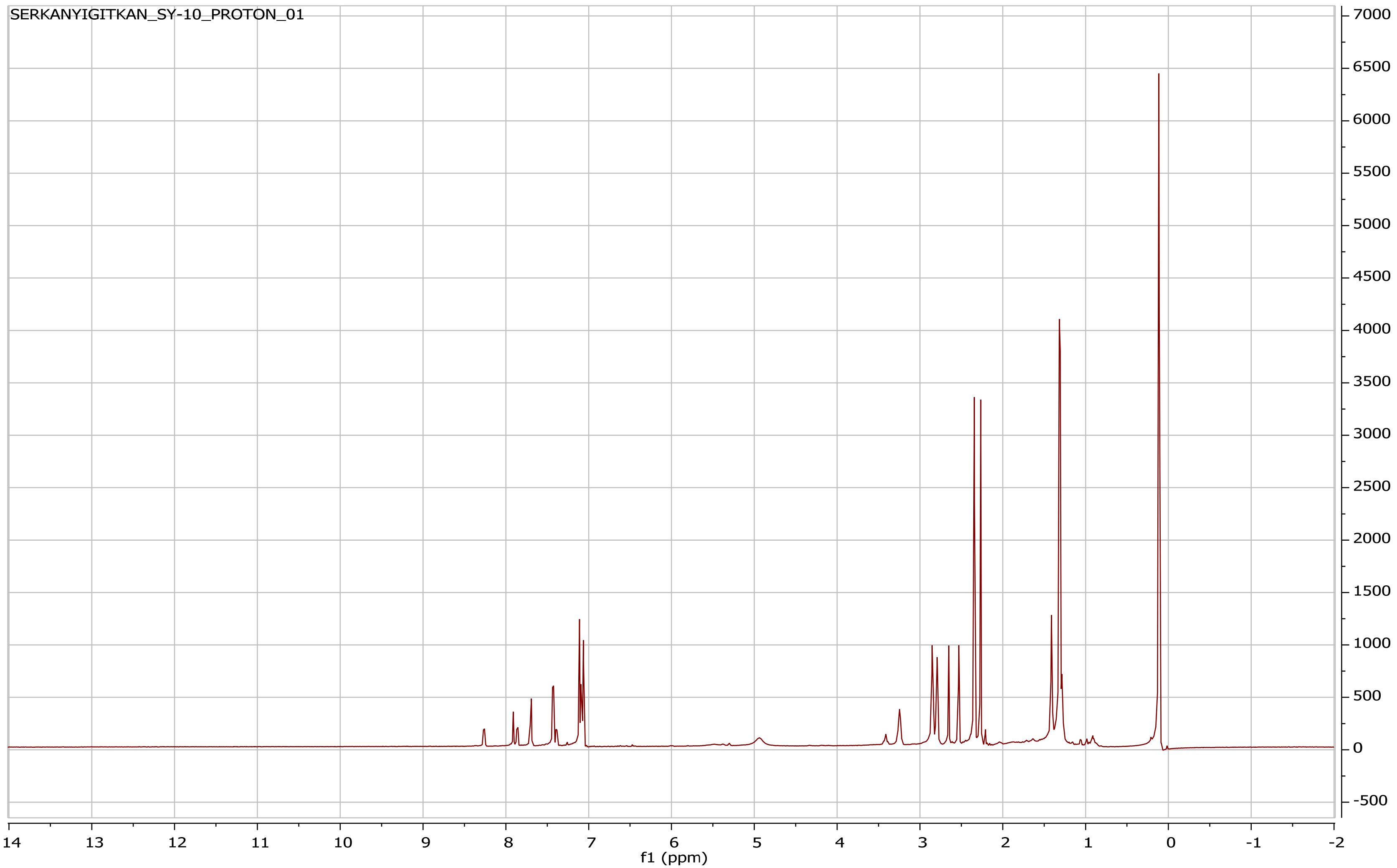

Figure S17. ^{1}H NMR spectrum of **4** in CD3OD (600 MHz)

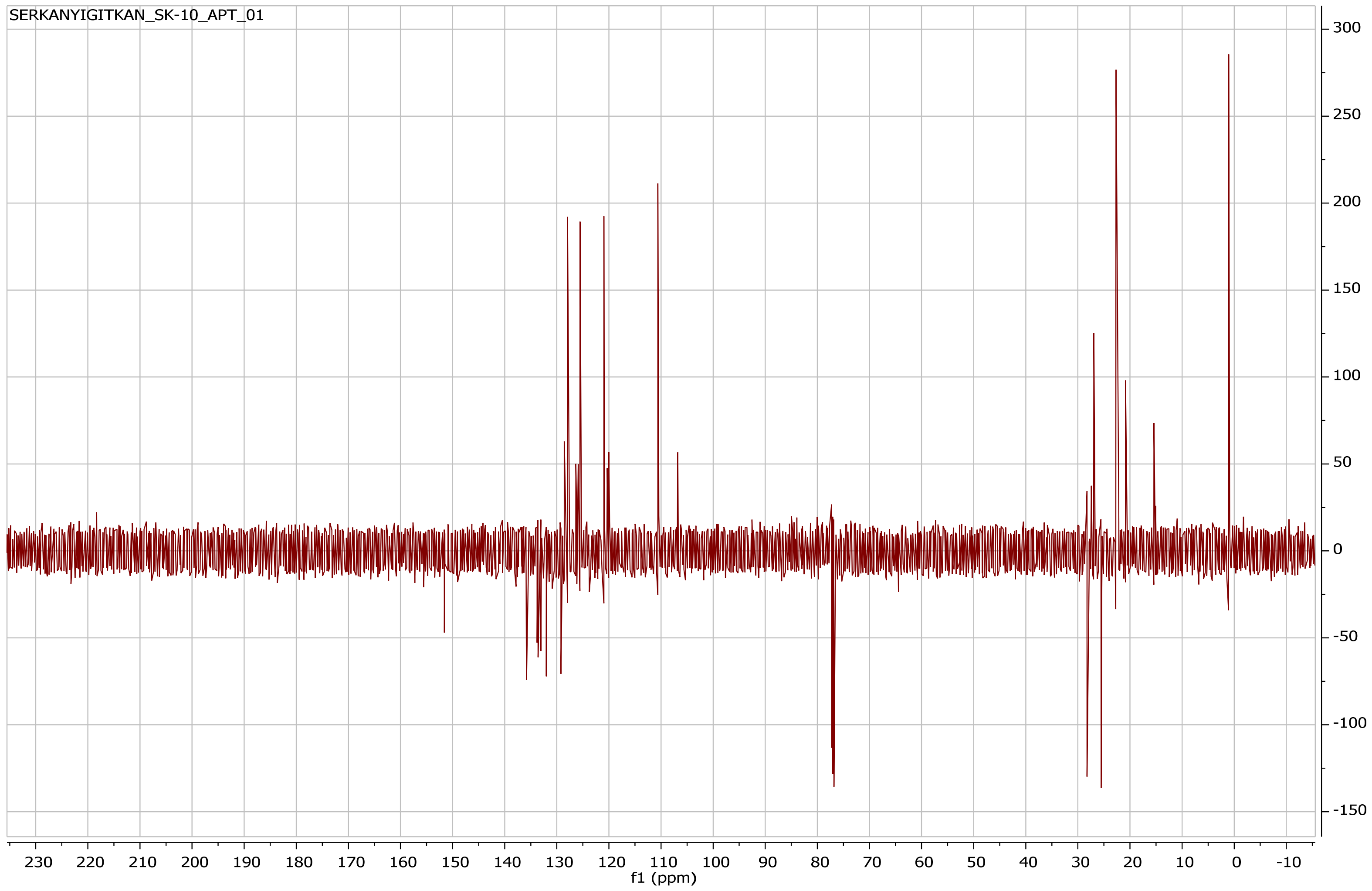

Figure S18. ^{13}C NMR (APT) spectrum of **4** in CD3OD (600 MHz)

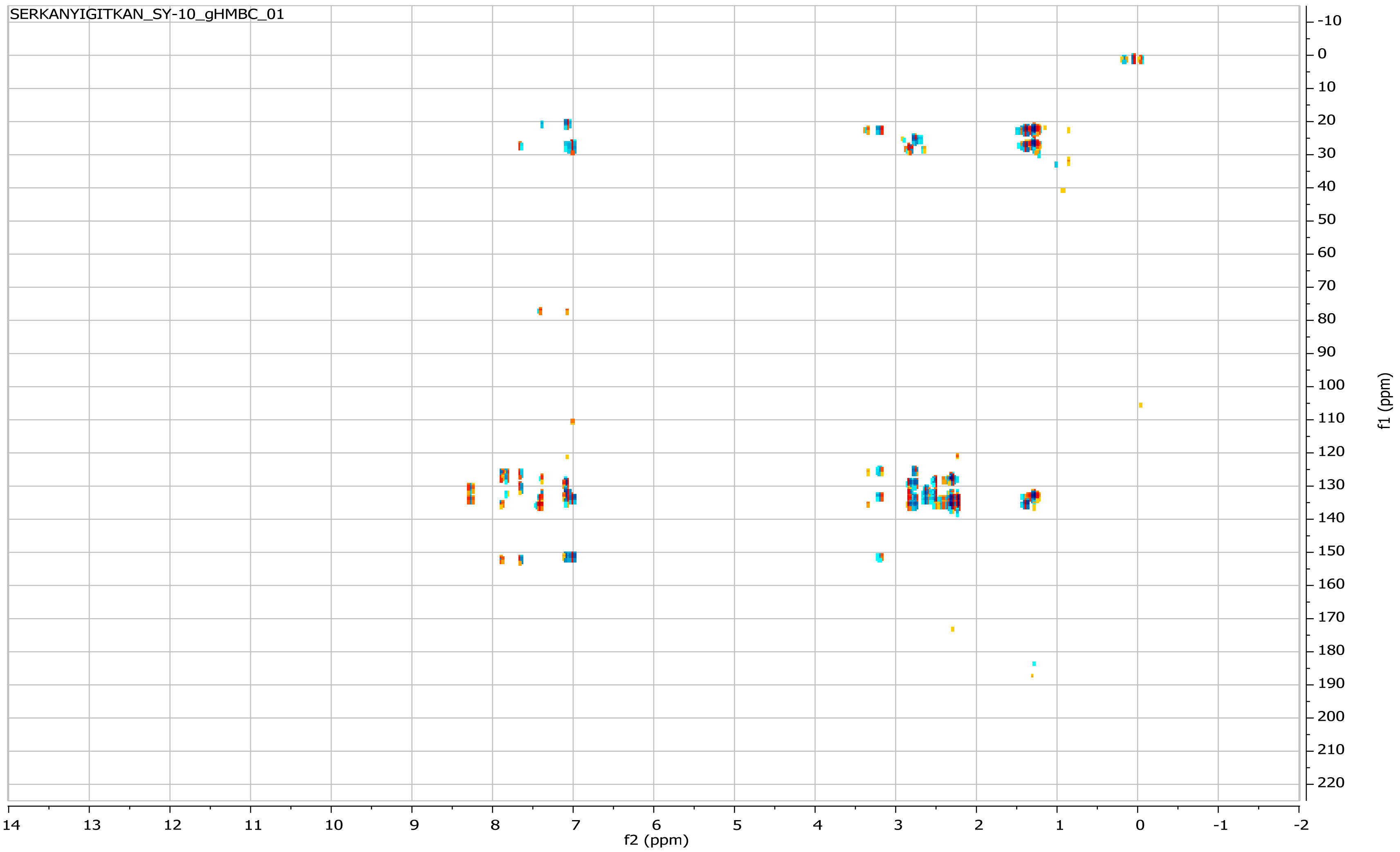

Figure S19. HMBC spectrum of **4** in CD_3OD (600 MHz)

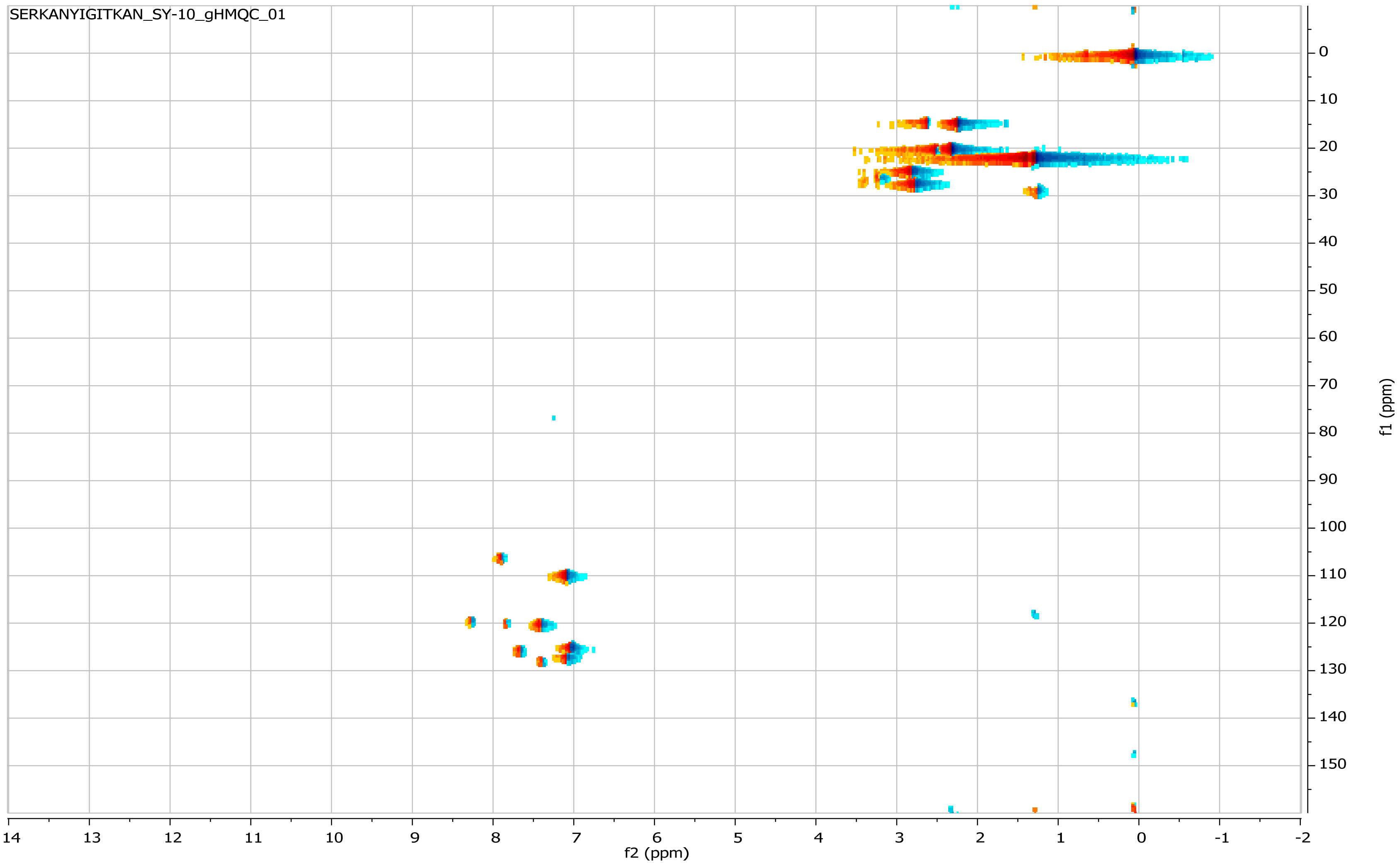

Figure S20. HMQC spectrum of **4** in CD_3OD (600 MHz)

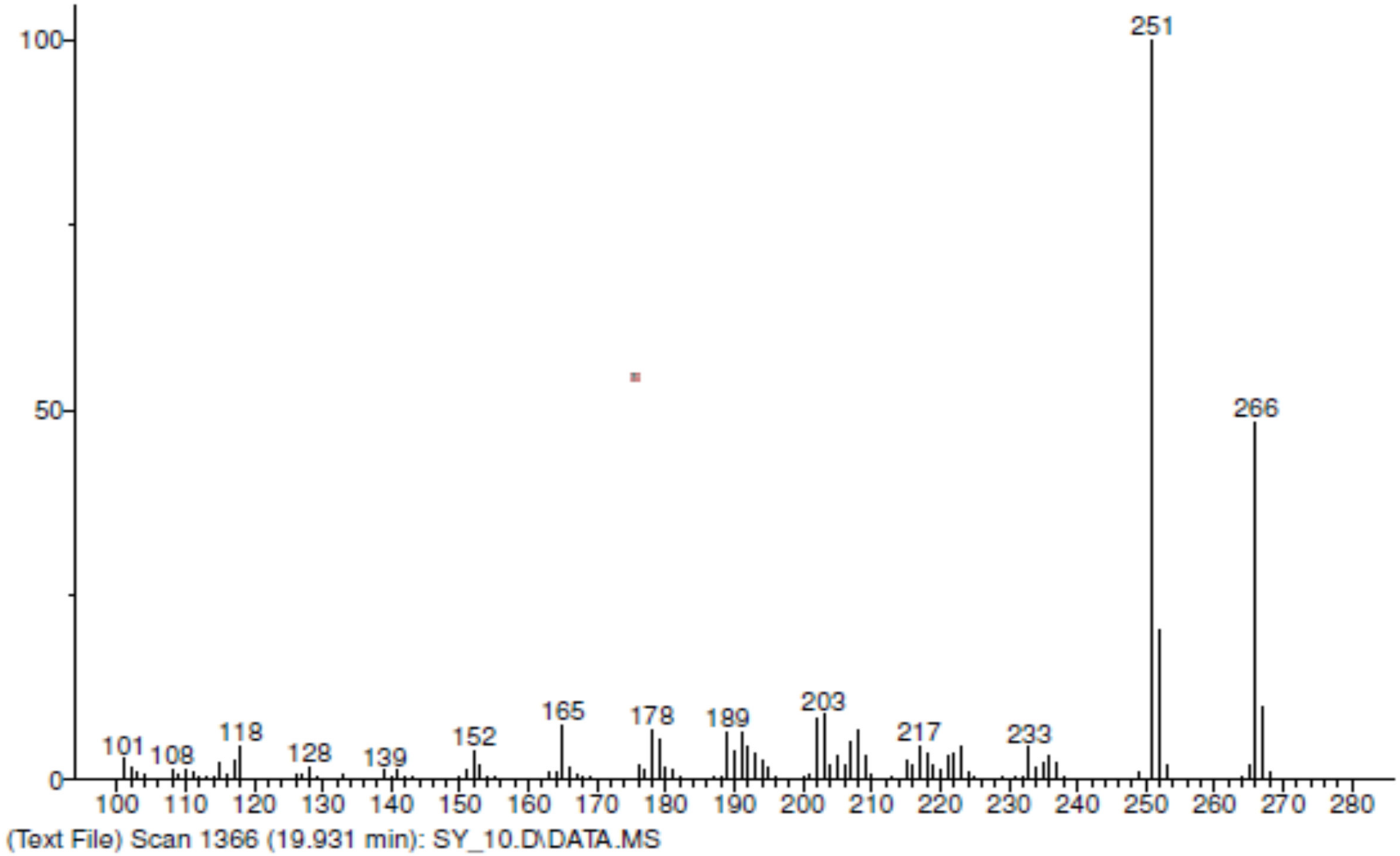

Figure S21. GC-MS spectrum of **4**

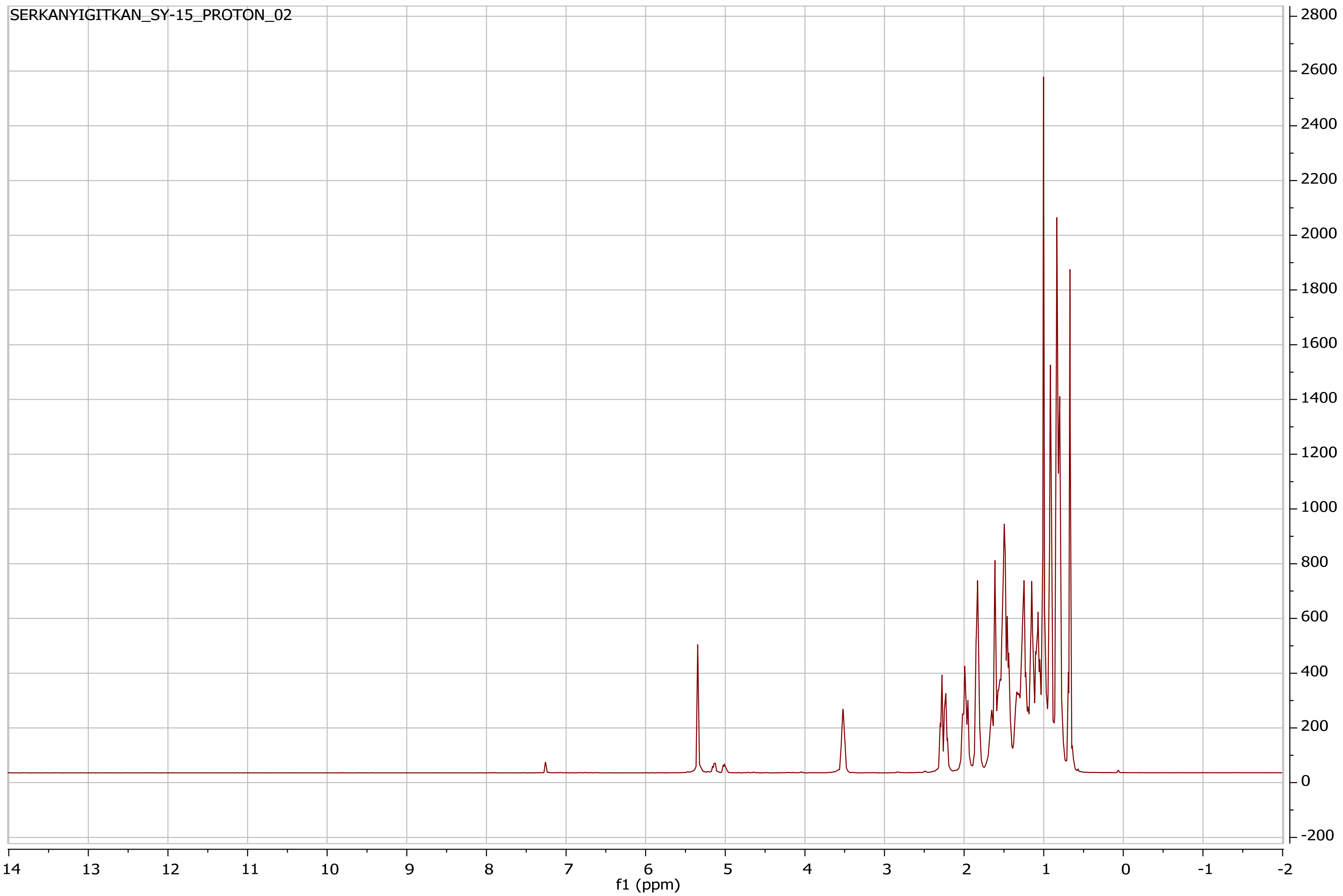

Figure S22. ^{1}H NMR spectrum of **5** in CD_3OD (600 MHz)

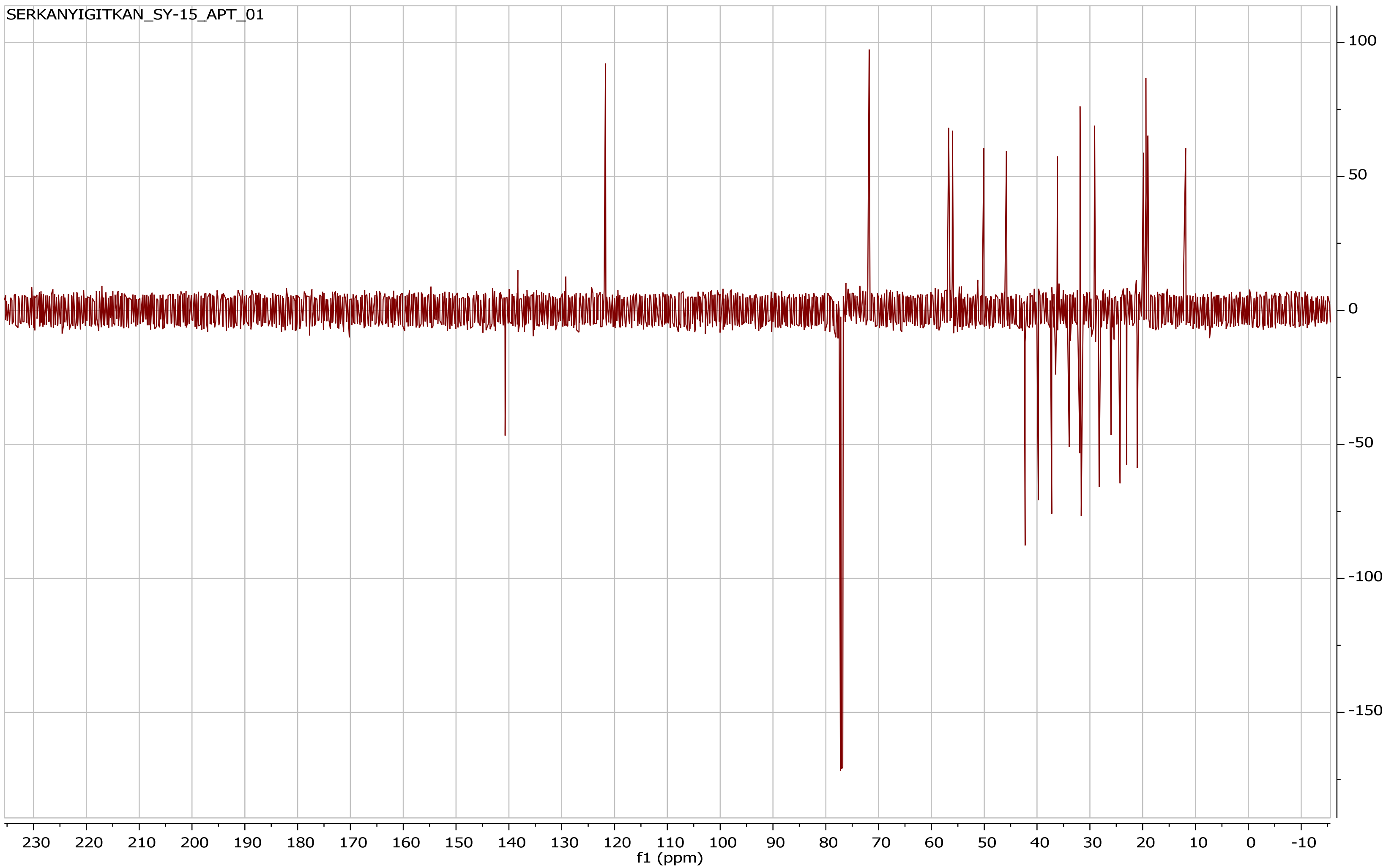

Figure S23. ^{13}C NMR (APT) spectrum of **5** in CD_3OD (600 MHz)

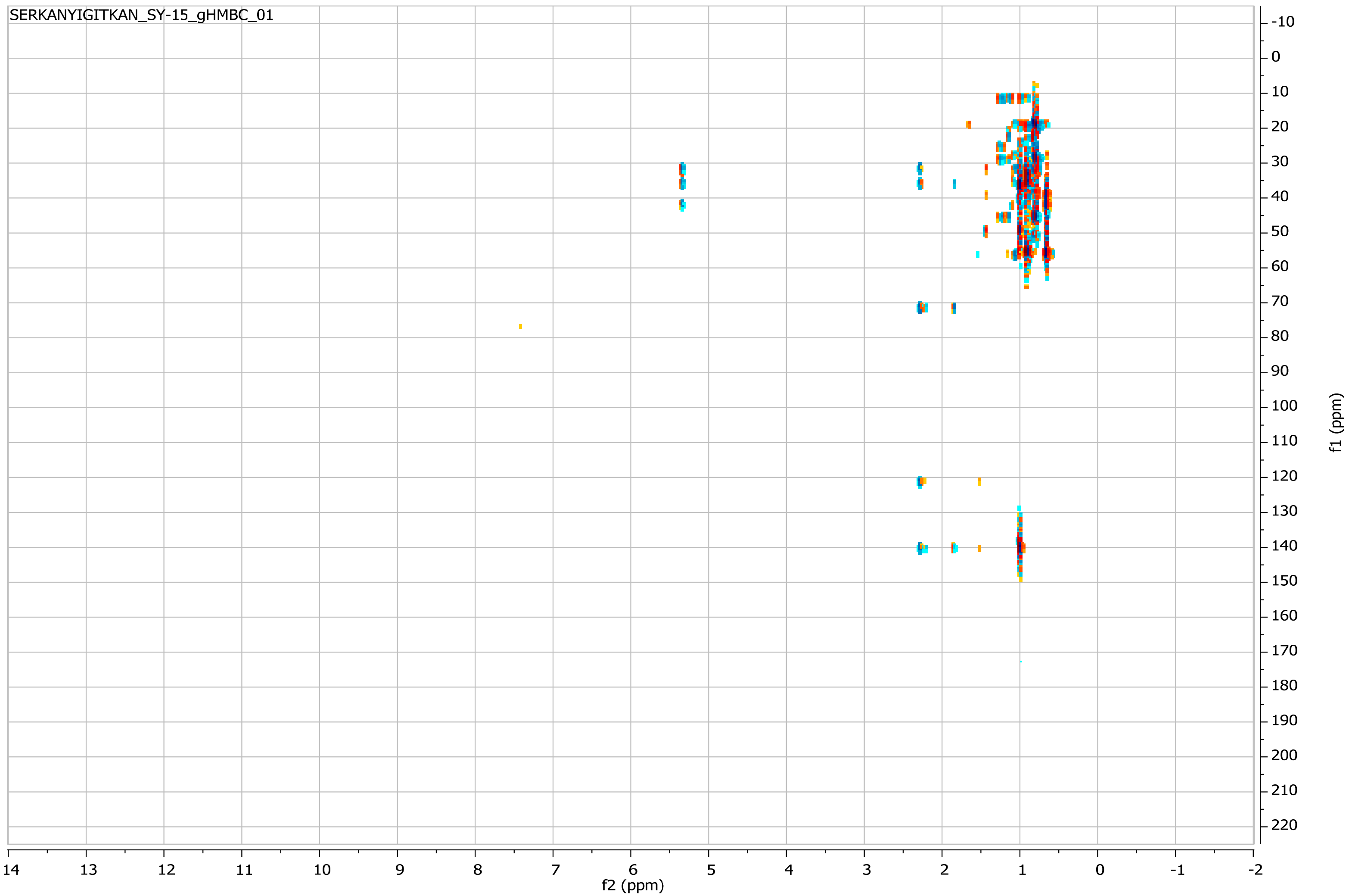

Figure S24. HMBC spectrum of **5** in CD_3OD (600 MHz)

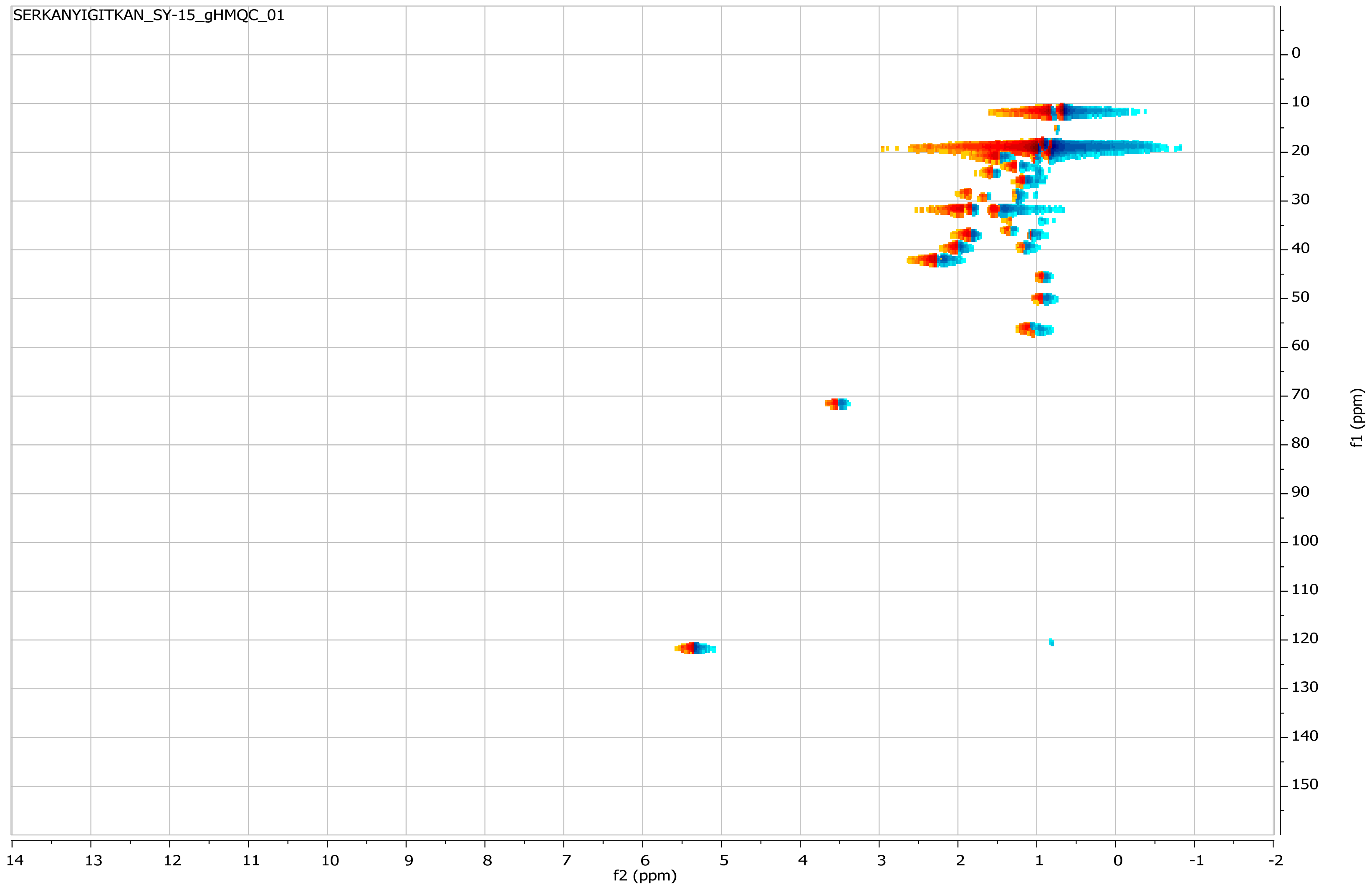

Figure S25. HMQC spectrum of **5** in CD_3OD (600 MHz)

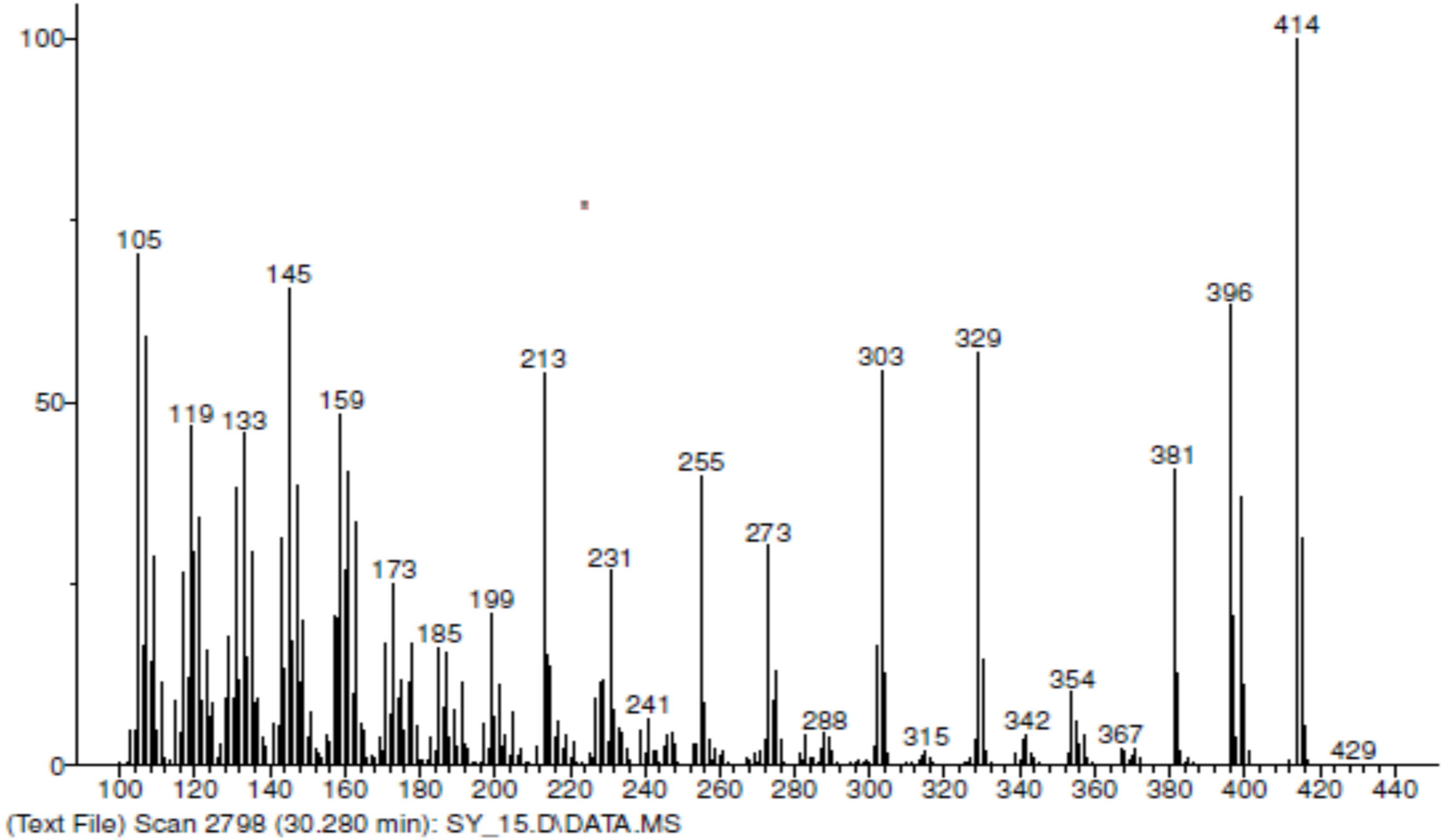

Figure S26. GC-MS spectrum of **5**

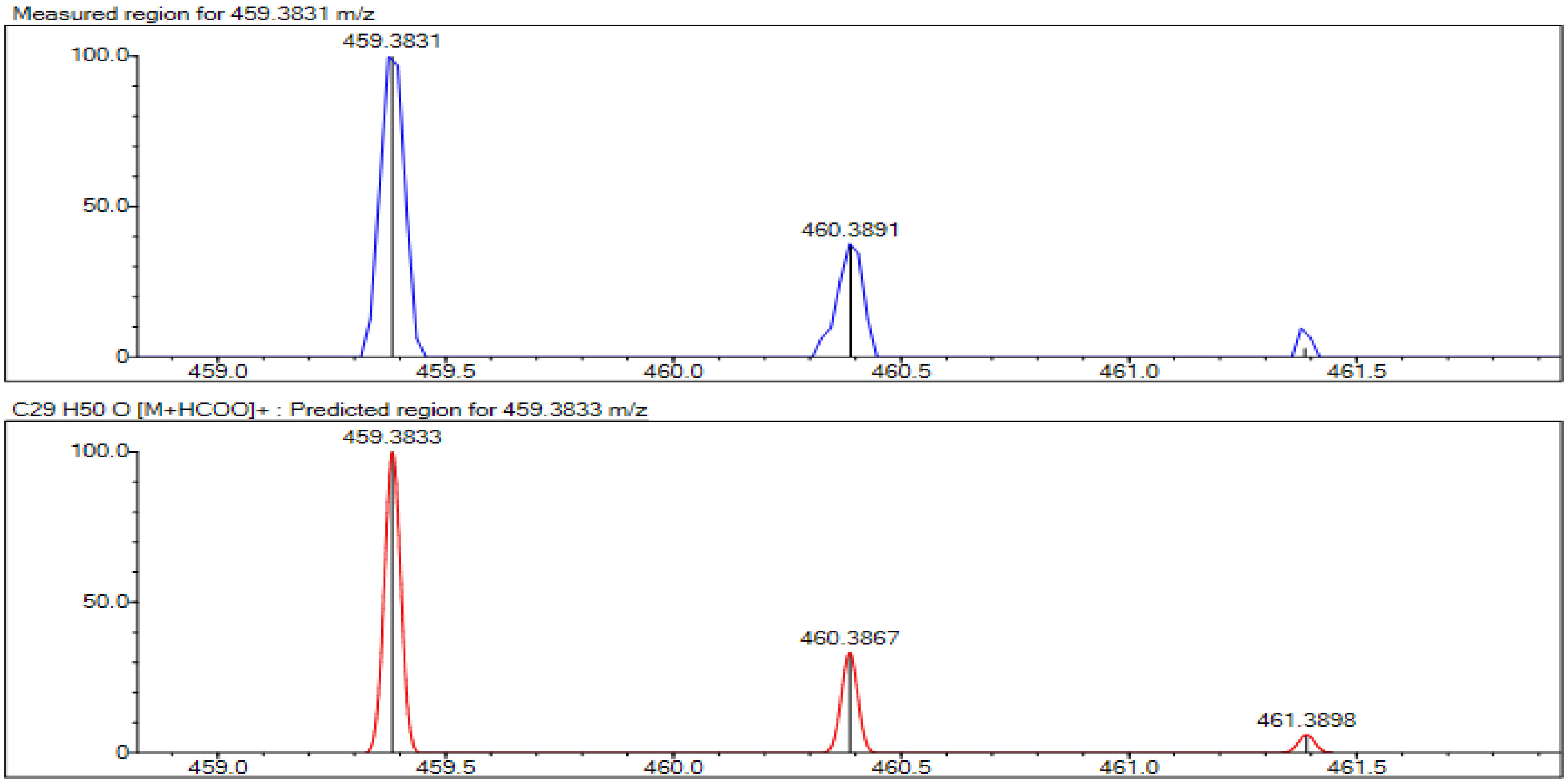

Rank	Score	Formula (M)	Ion	Meas. m/z	Pred. m/z	Df. (mDa)	Df. (ppm)	Iso	DBE
1	76.58	C29 H50 O	[M+HCOO]+	459.3831	459.3833	-0.2	-0.44	76.58	5.0

Figure S27. LC-MS-IT-TOF spectrum of **5**

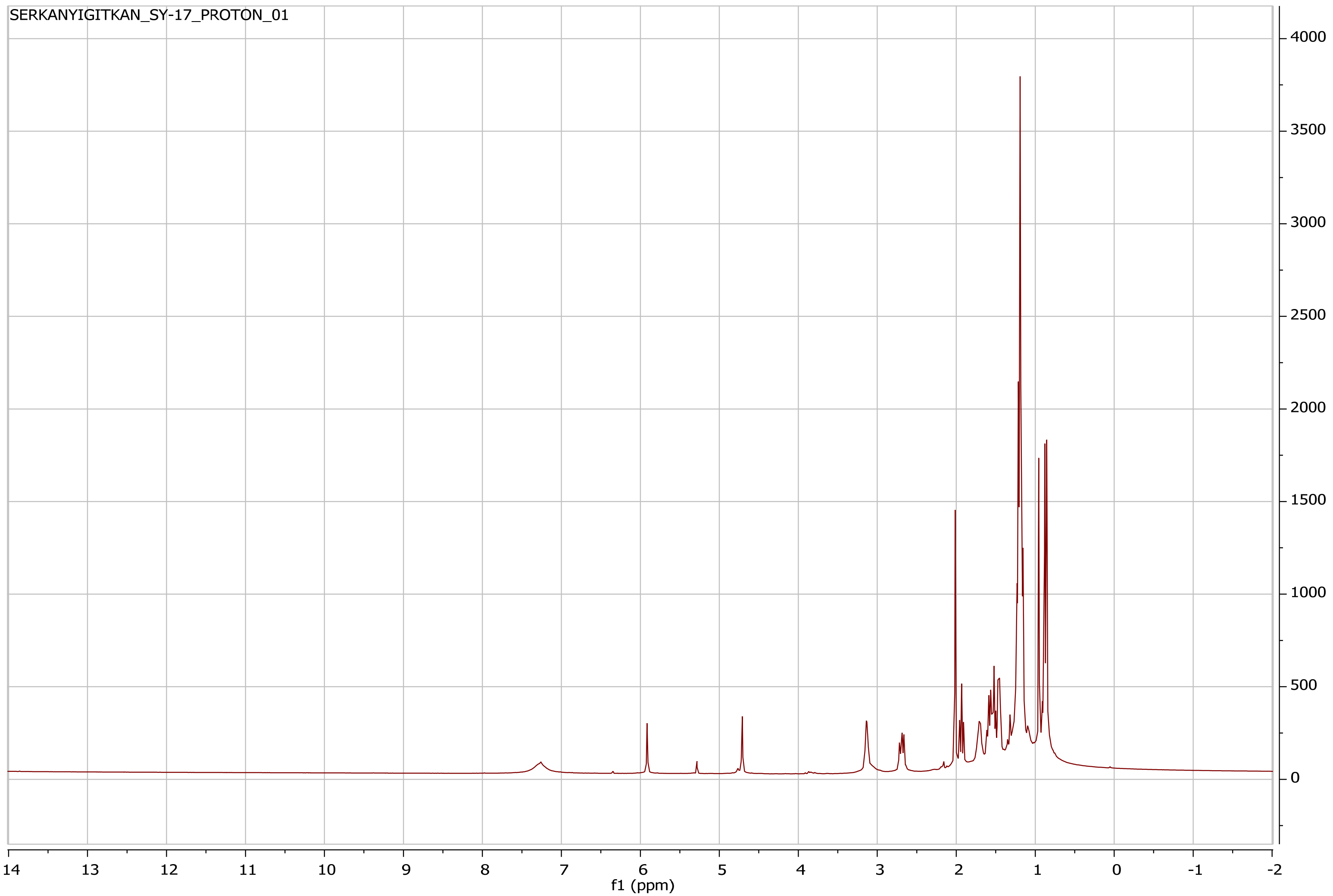

Figure S28. ^{1}H NMR spectrum of **6** in CD3OD (600 MHz)

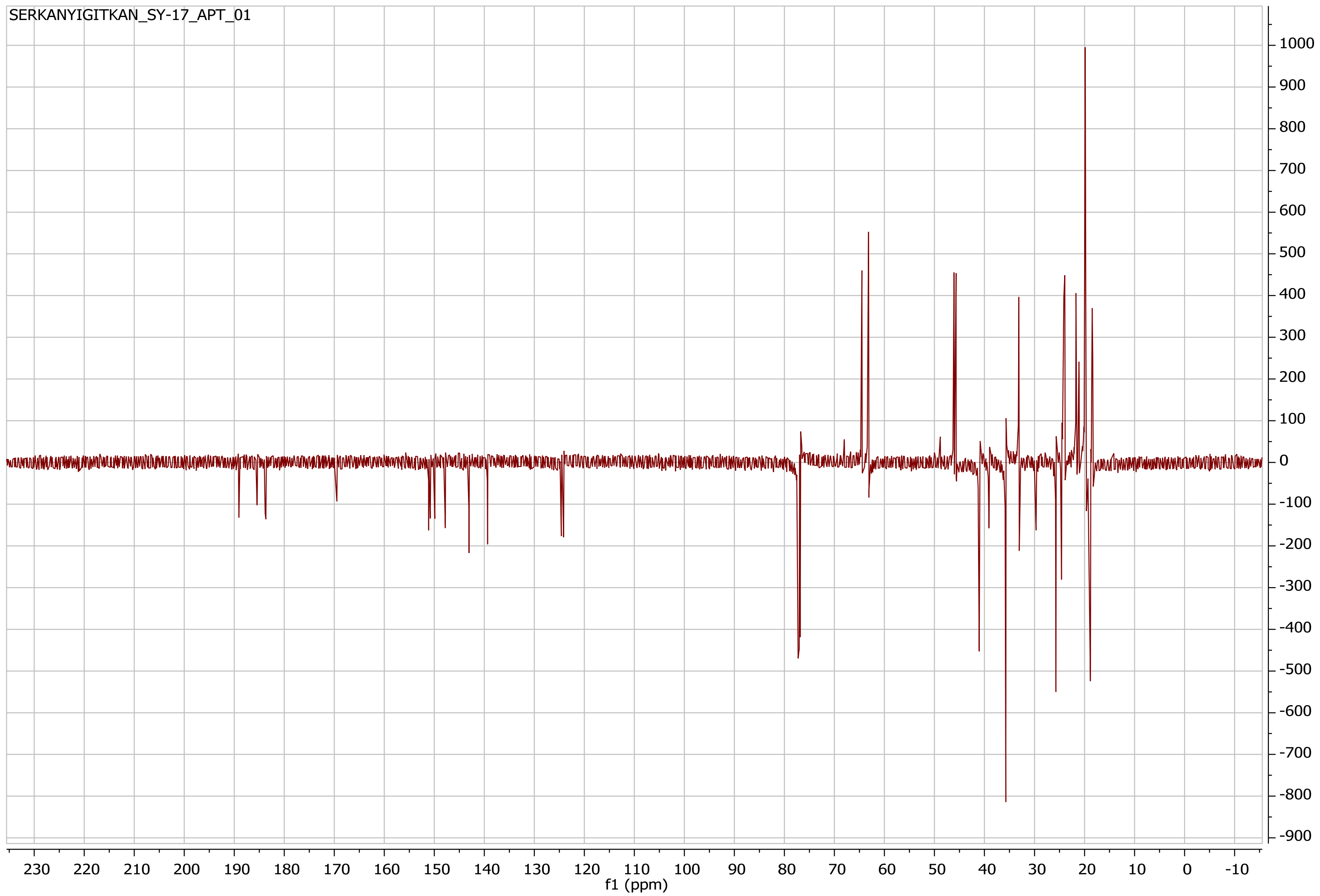

Figure S29. ^{13}C NMR (APT) spectrum of **6** in CD3OD (600 MHz)

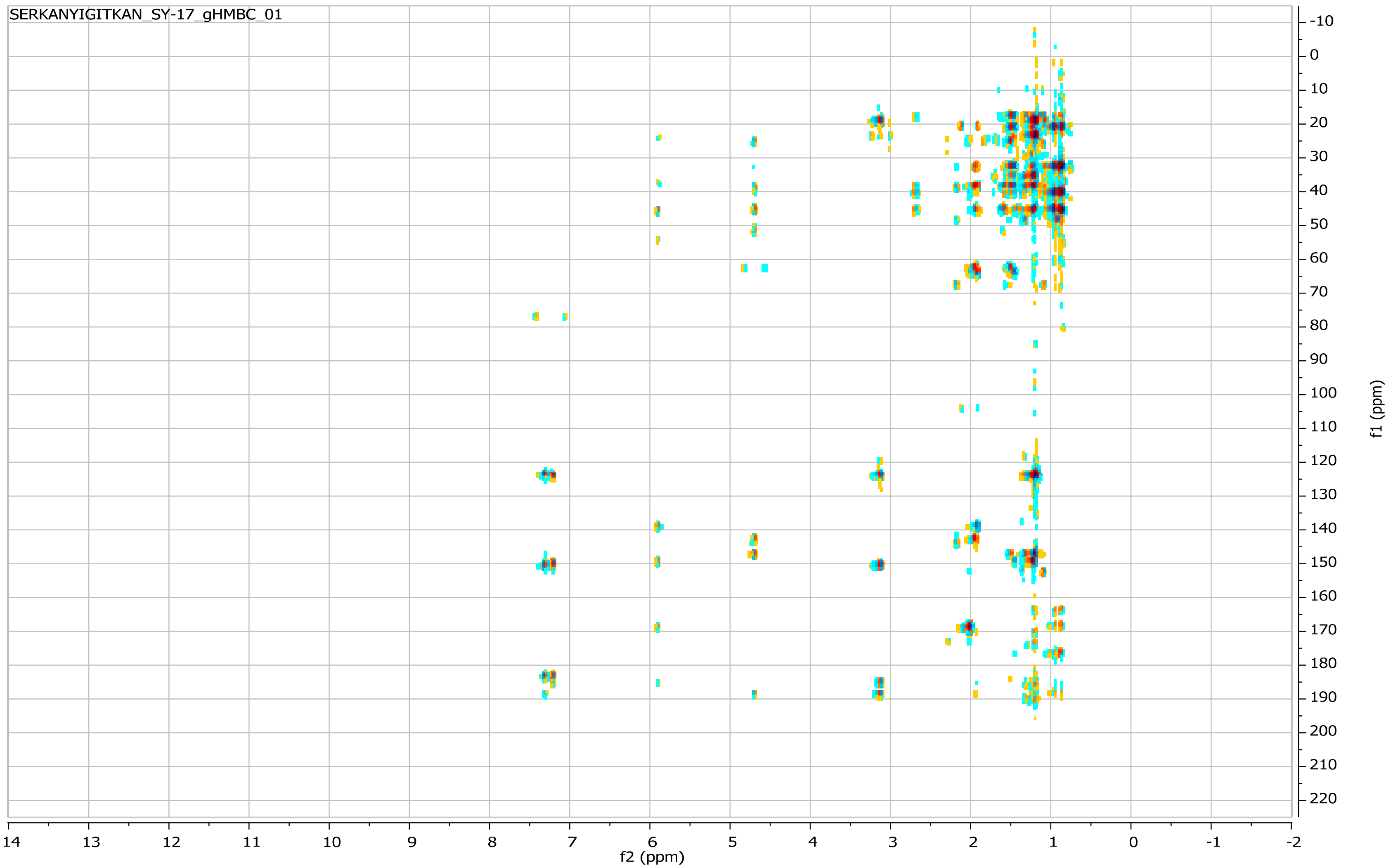

Figure S30. HMBC spectrum of **6** in CD3OD (600 MHz)

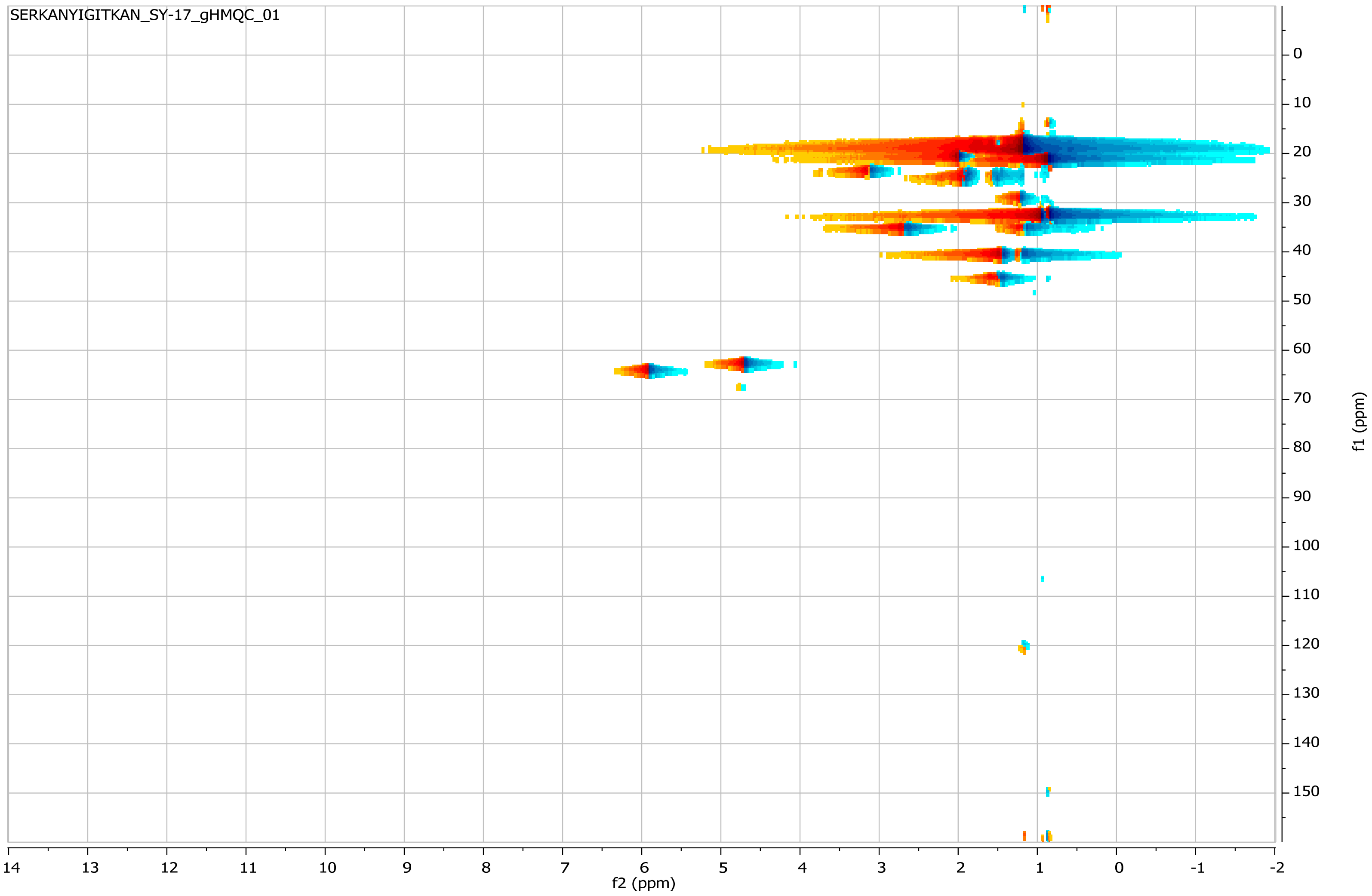

Figure S31. HMQC spectrum of **6** in CD_3OD (600 MHz)

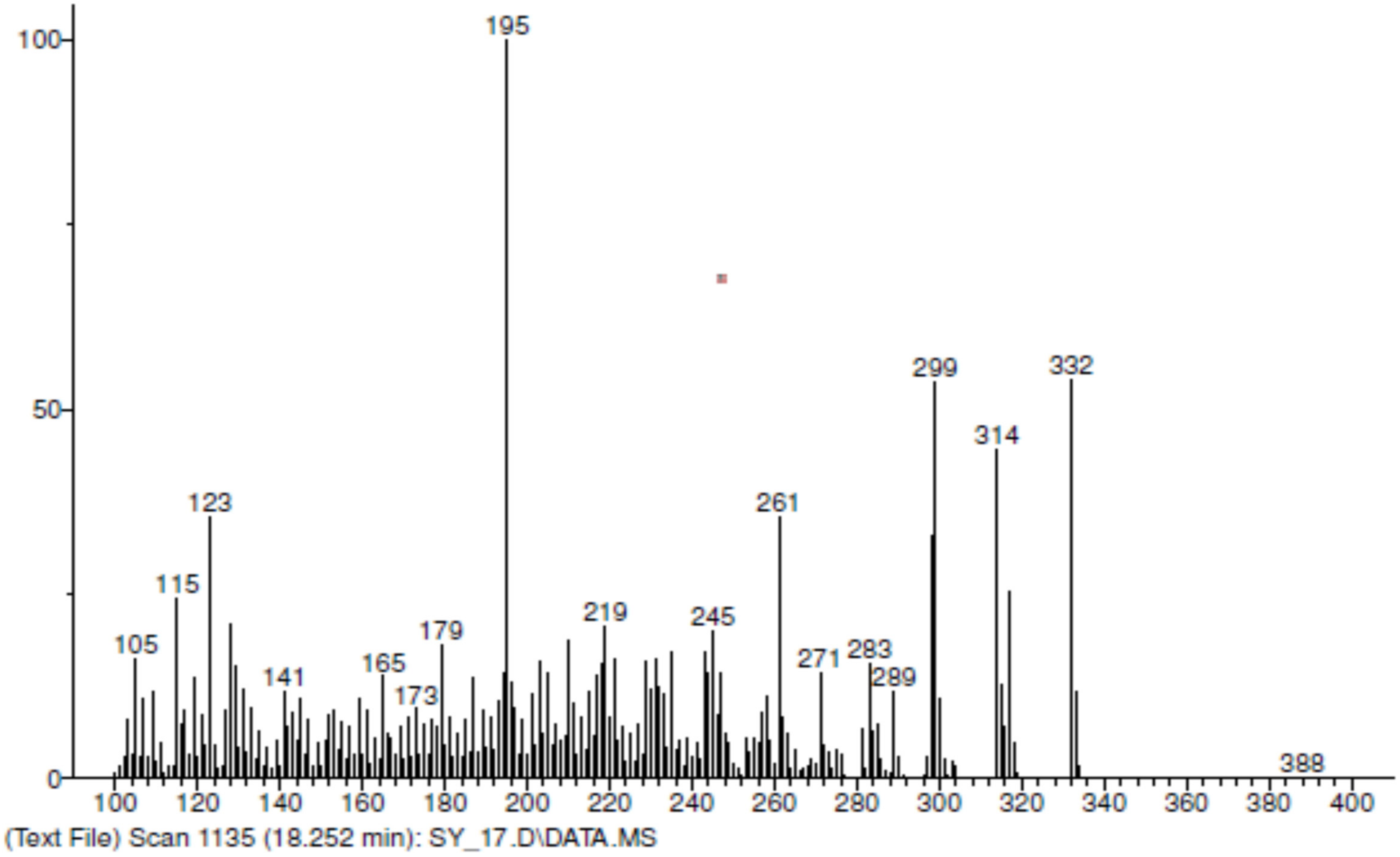

Figure S32. GC-MS spectrum of **6**

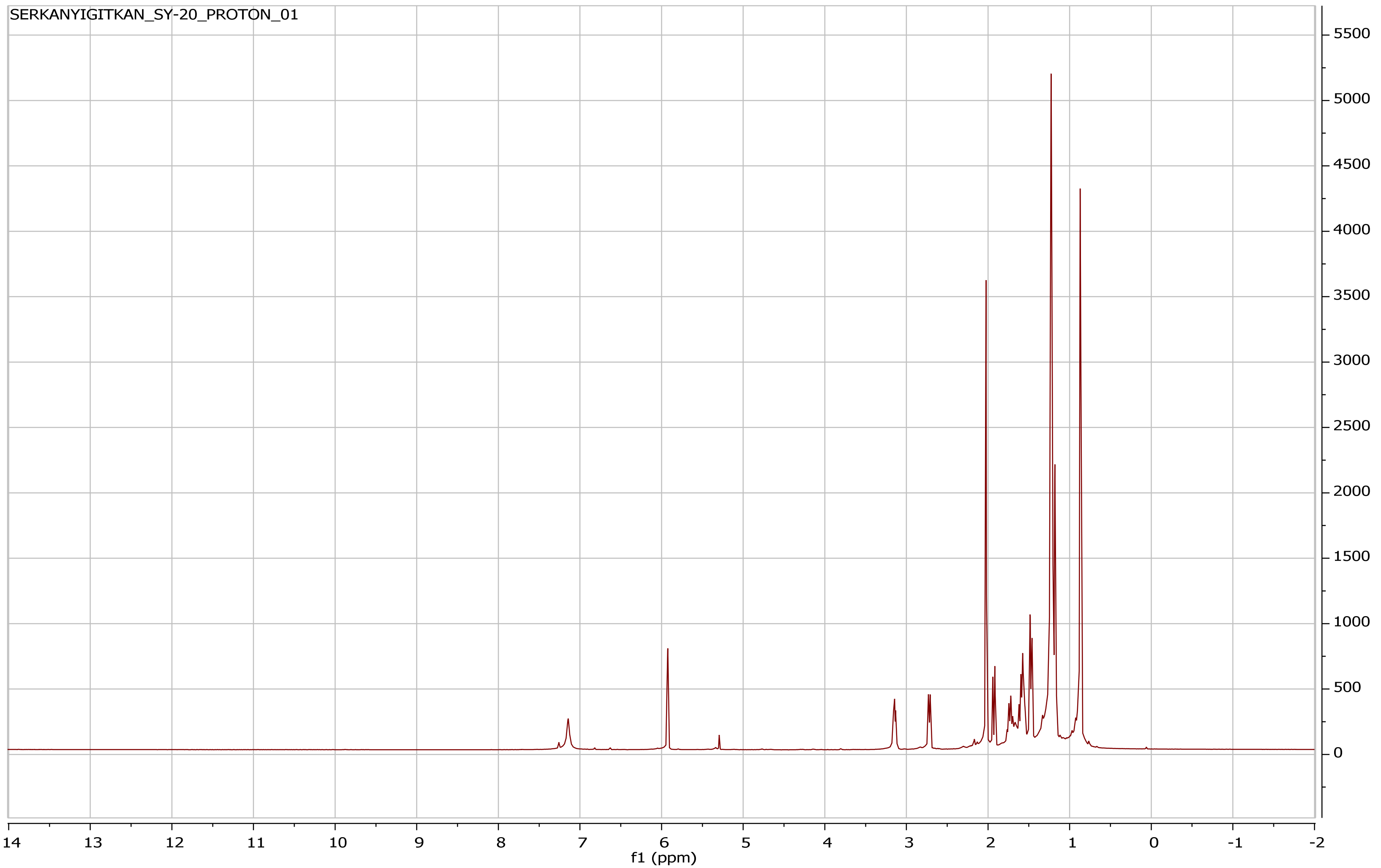

Figure S33. ^{1}H NMR spectrum of **7** in CD3OD (600 MHz)

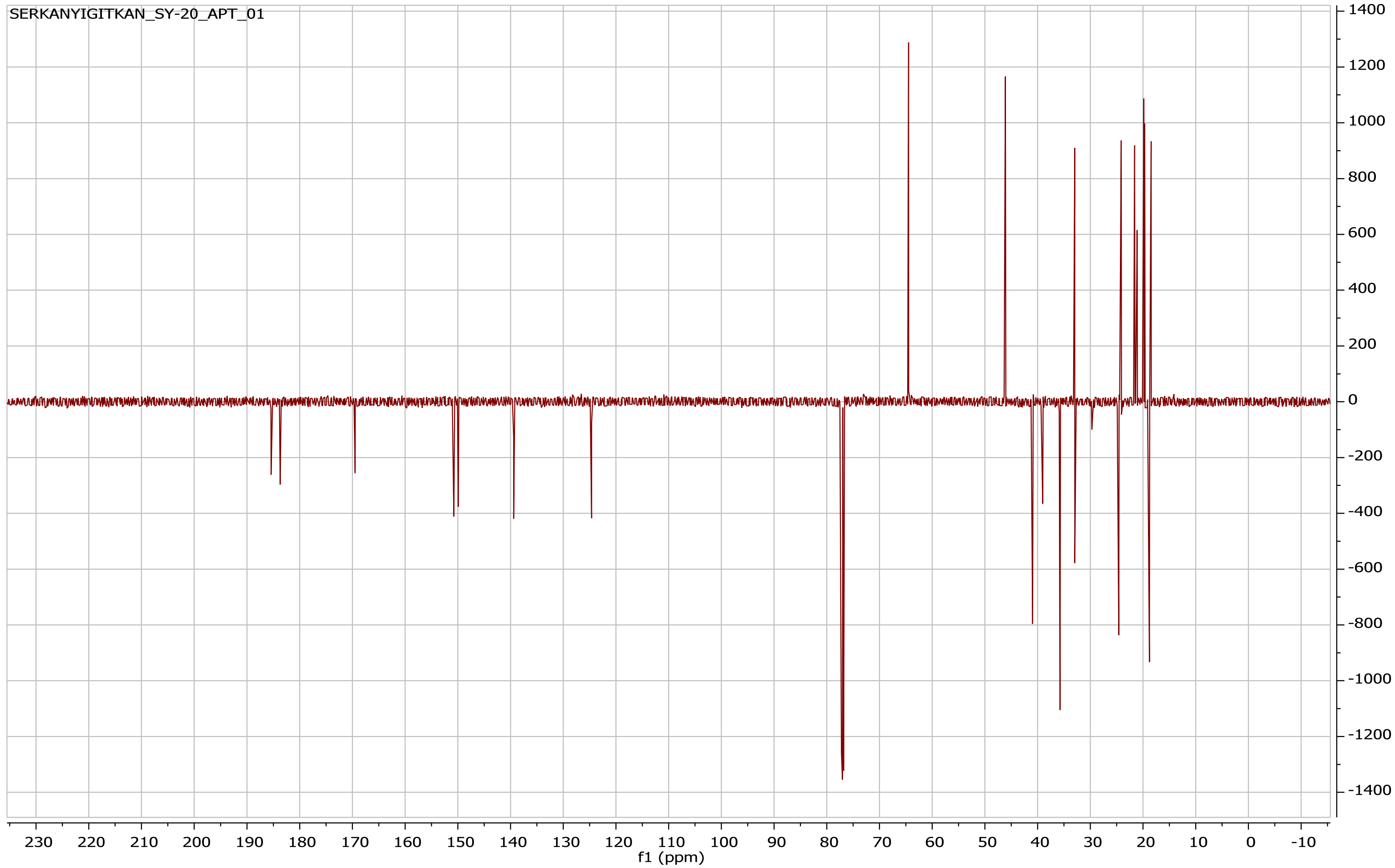

Figure S34. ^{13}C NMR (APT) spectrum of **7** in CD3OD (600 MHz)

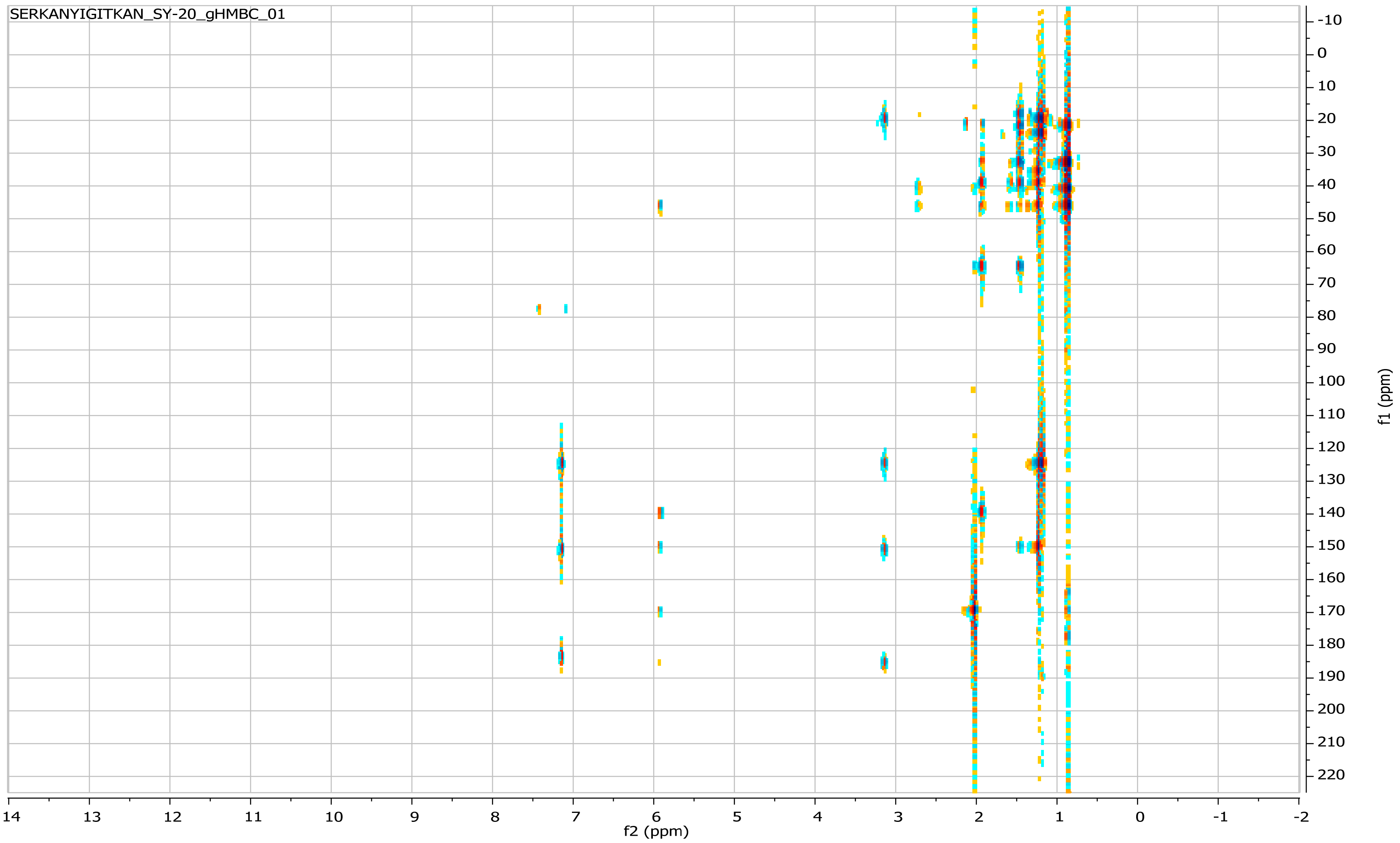

Figure S35. HMBC spectrum of **7** in CD3OD (600 MHz)

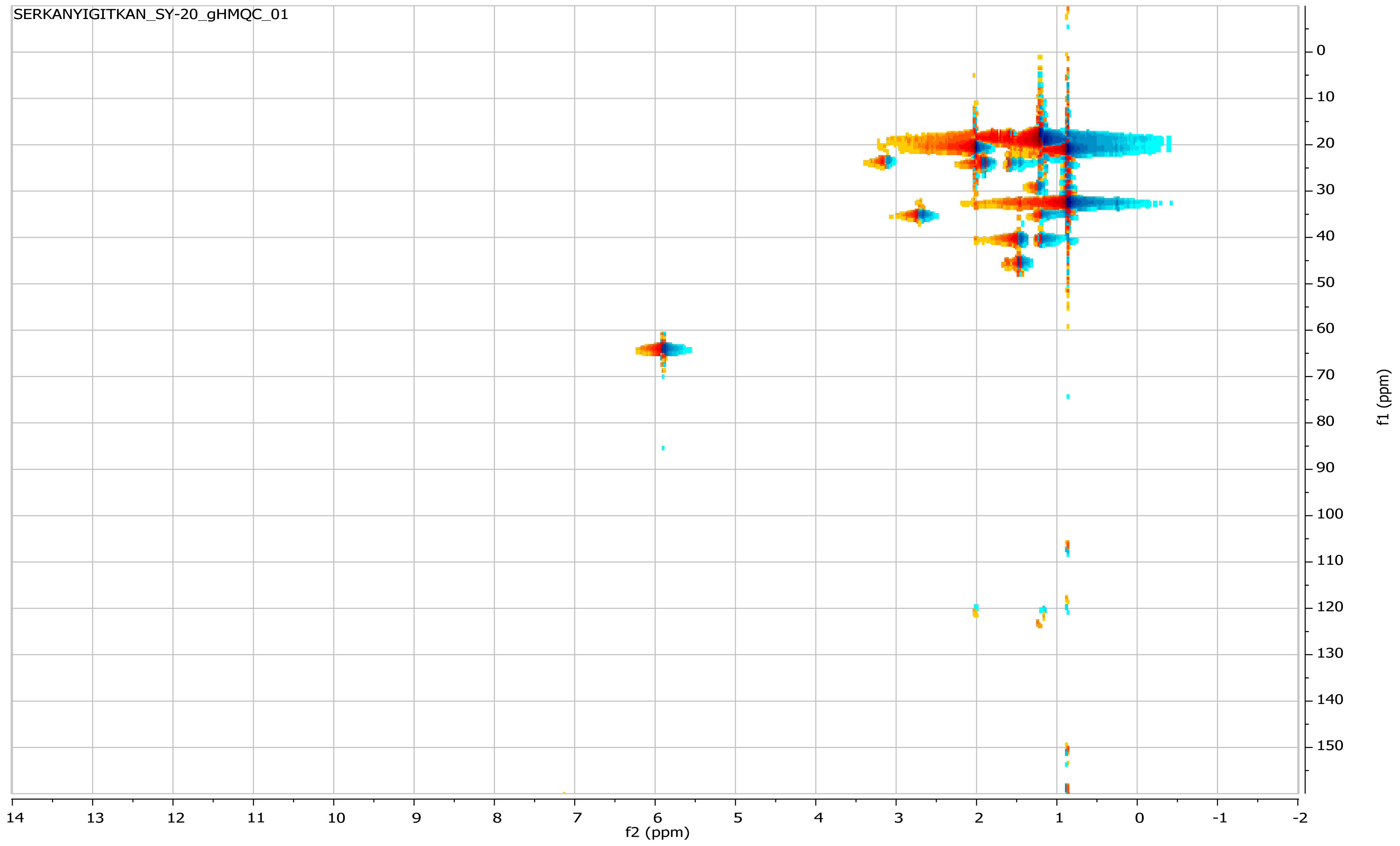

Figure S36. HMQC spectrum of **7** in CD_3OD (600 MHz)

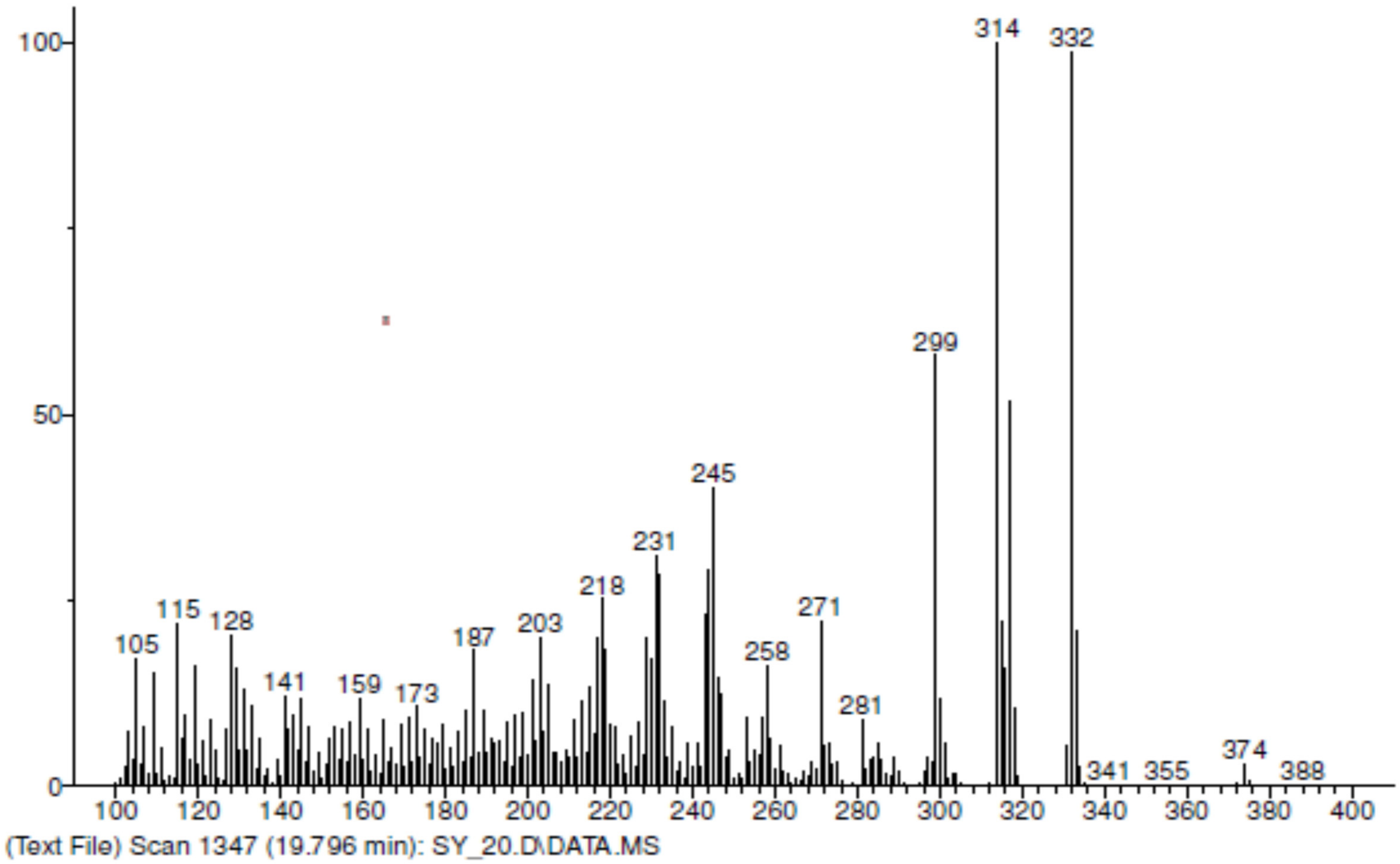

Figure S37. GC-MS spectrum of **7**

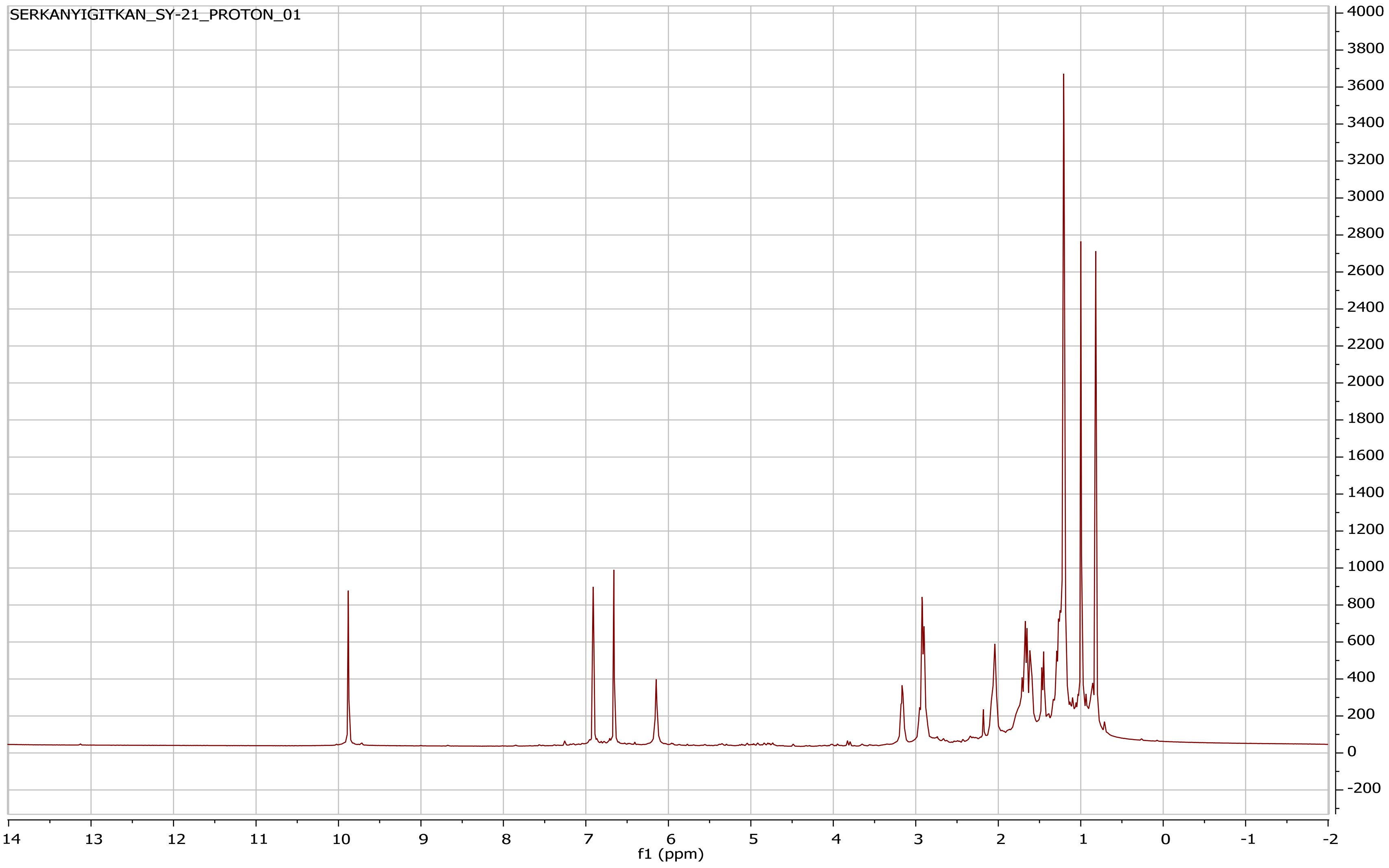

Figure S38. ^{1}H NMR spectrum of **8** in CD_3OD (600 MHz)

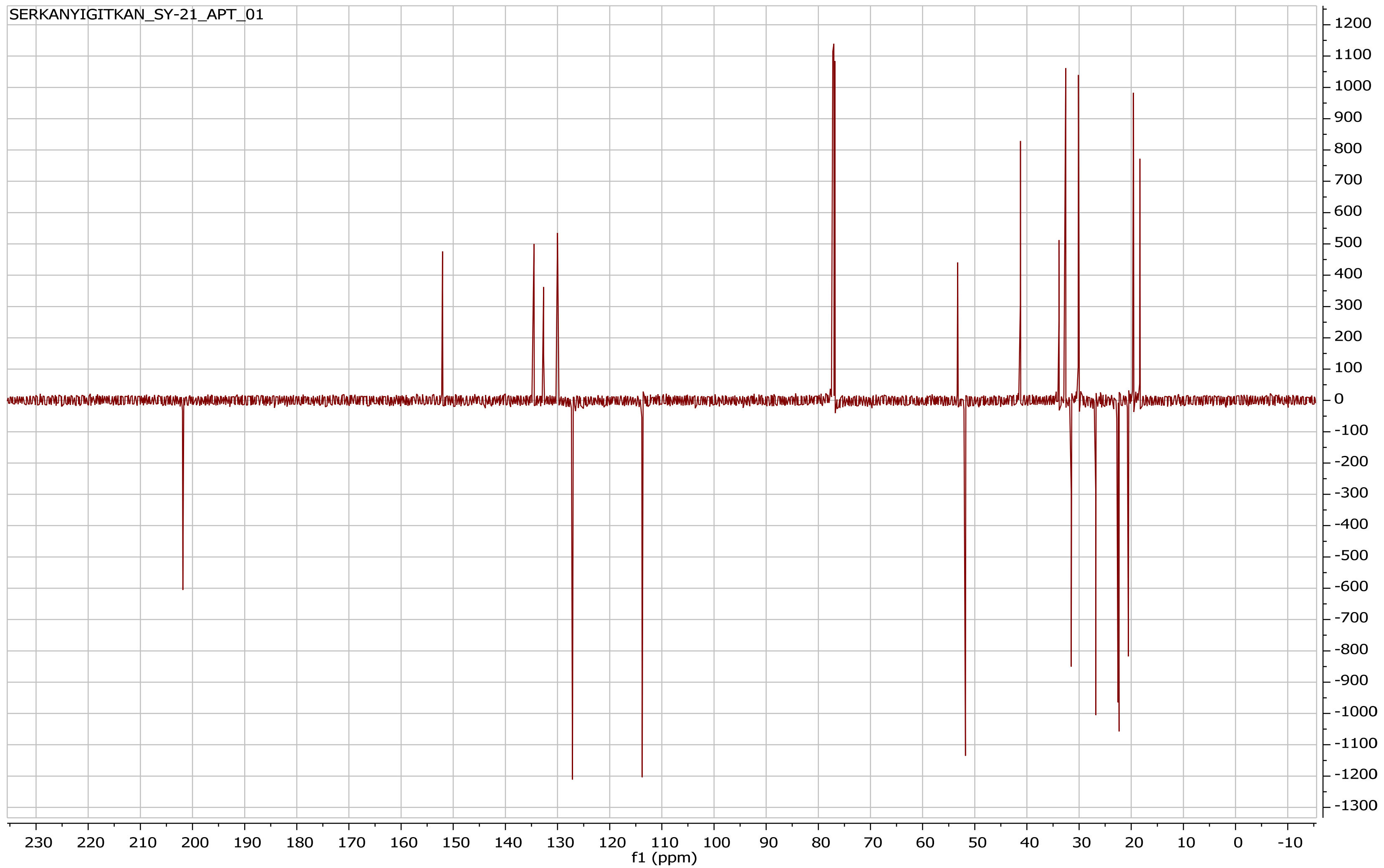

Figure S39. ^{13}C NMR (APT) spectrum of **8** in CD3OD (600 MHz)

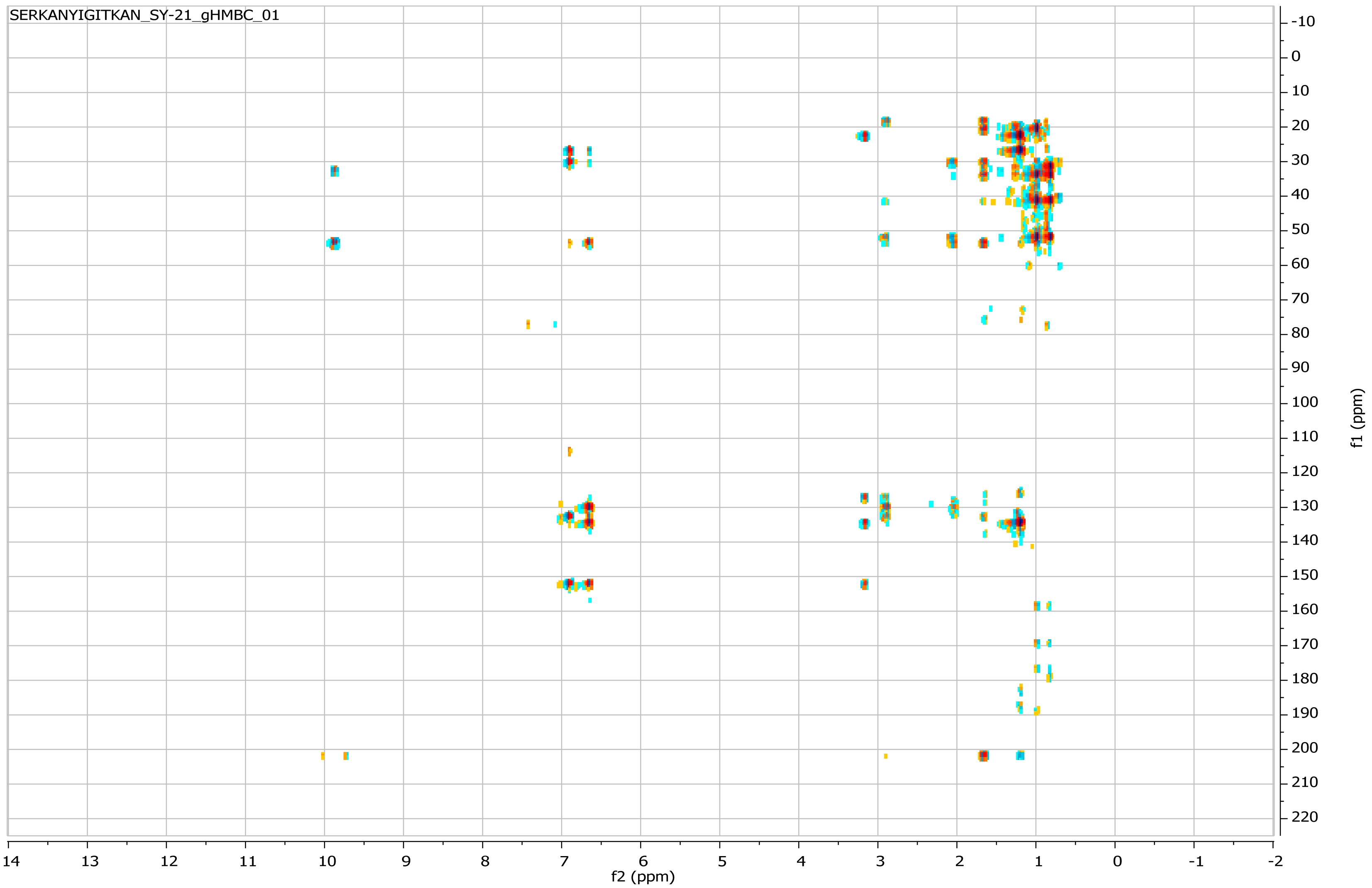

Figure S40. HMBC spectrum of **8** in CD_3OD (600 MHz)

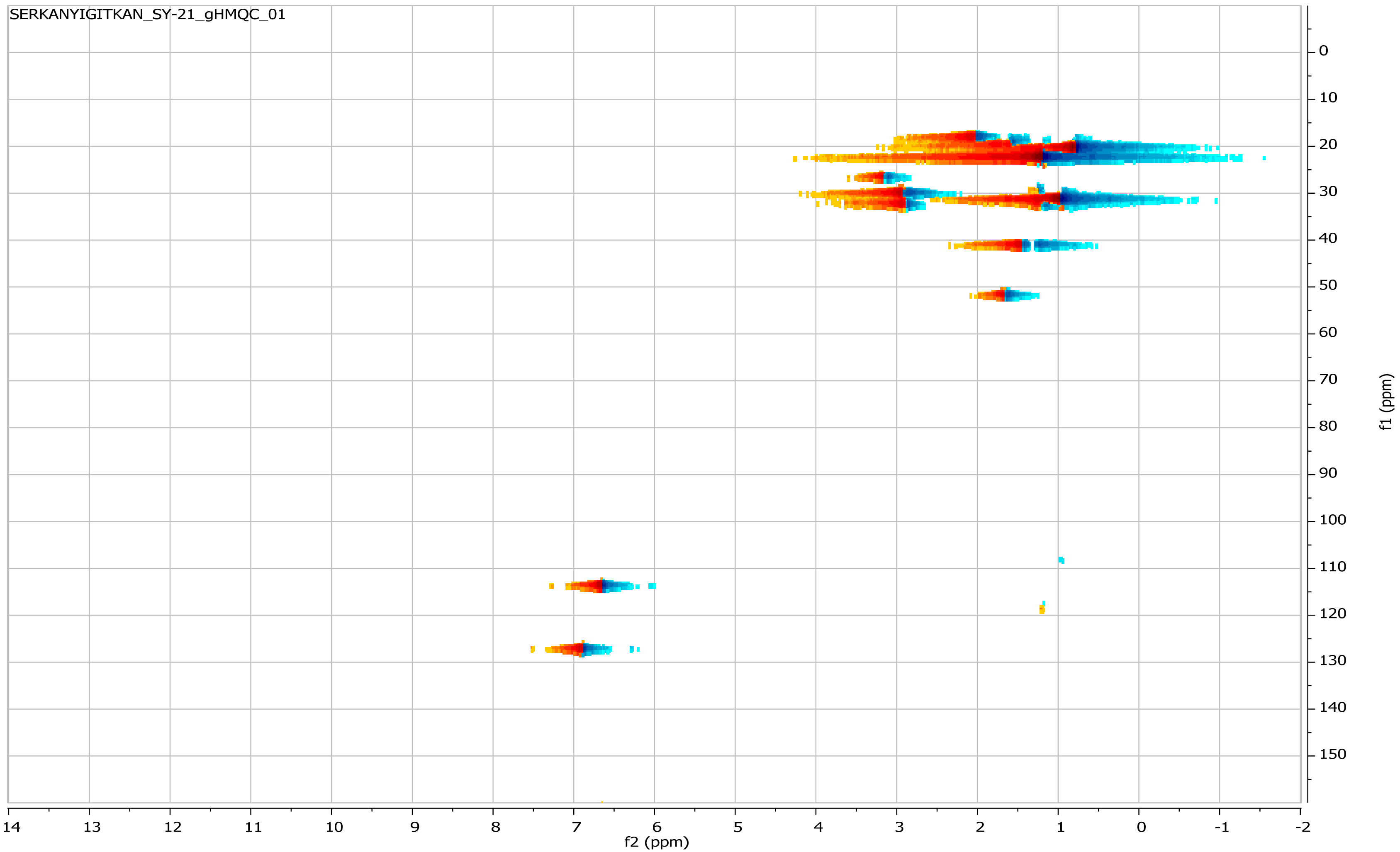

Figure S41. HMQC spectrum of **8** in CD_3OD (600 MHz)

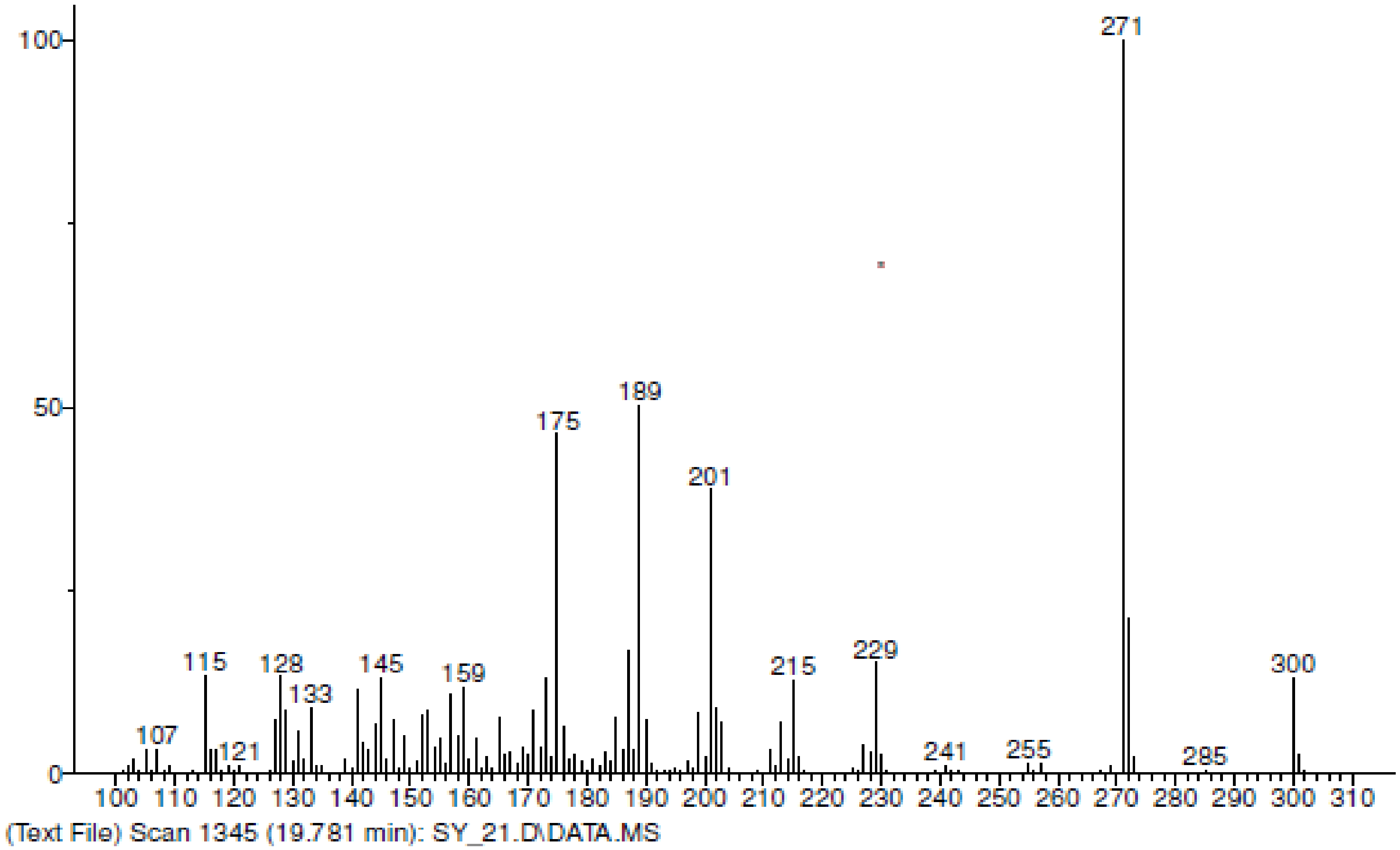

Figure S42. GC-MS spectrum of **8**

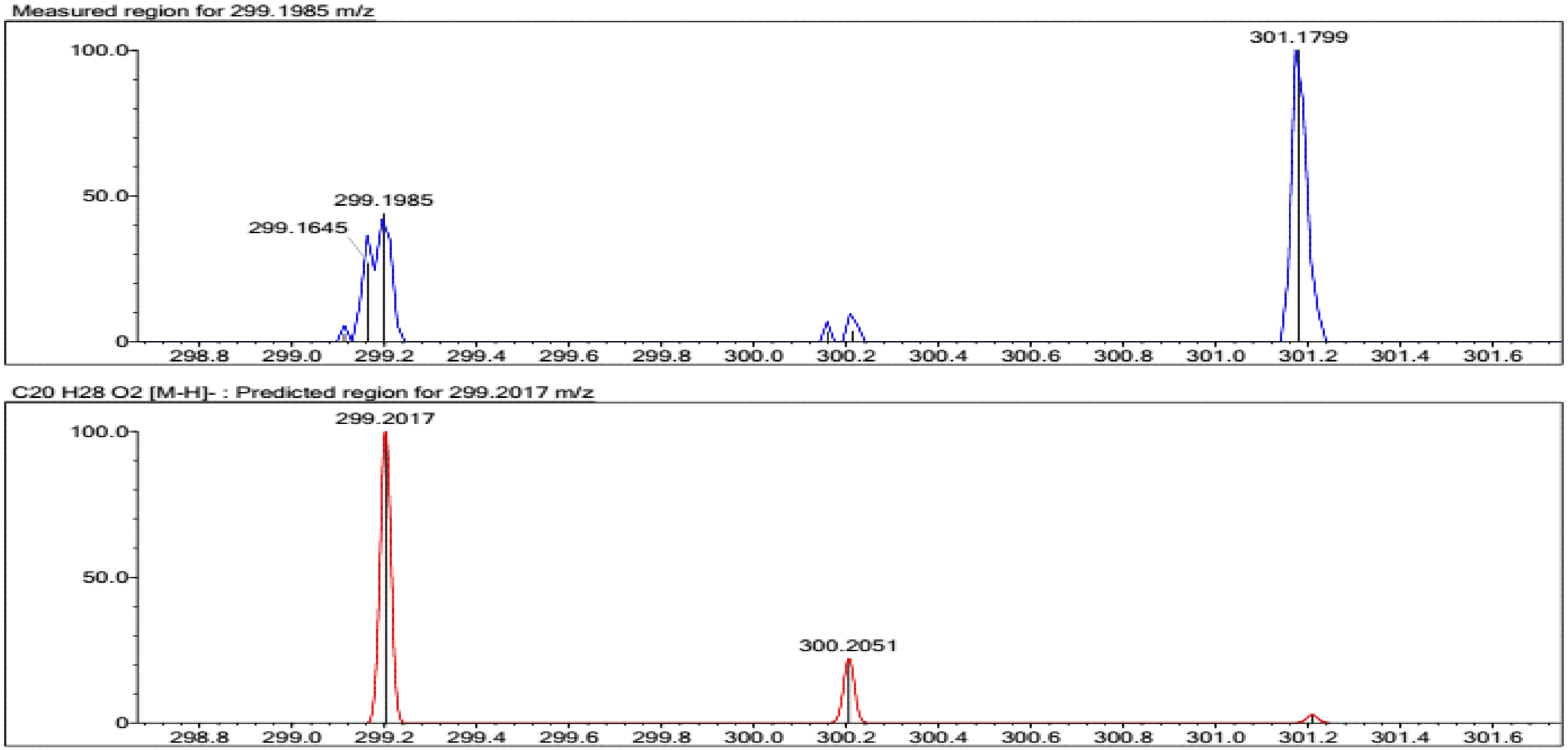

Rank	Score	Formula (M)	Ion	Meas. m/z	Pred. m/z	Df. (mDa)	Df. (ppm)	Iso	DBE
1	2.30	C20 H28 O2	[M-H]-	299.1985	299.2017	-3.2	-10.70	6.03	7.0

Figure S43. LC-MS-IT-TOF spectrum of **8**

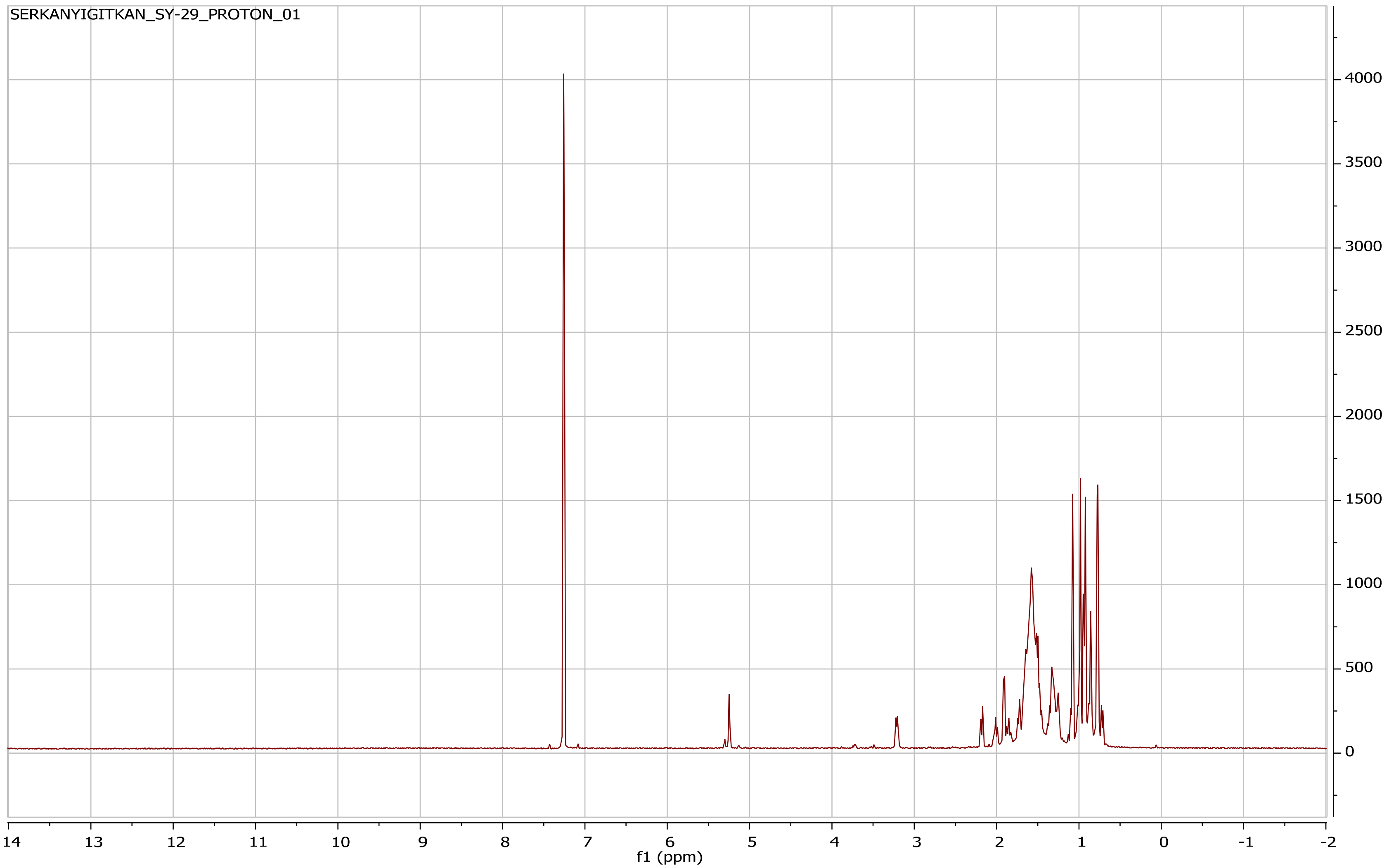

Figure S44. ^{1}H NMR spectrum of **9** in CD_3OD (600 MHz)

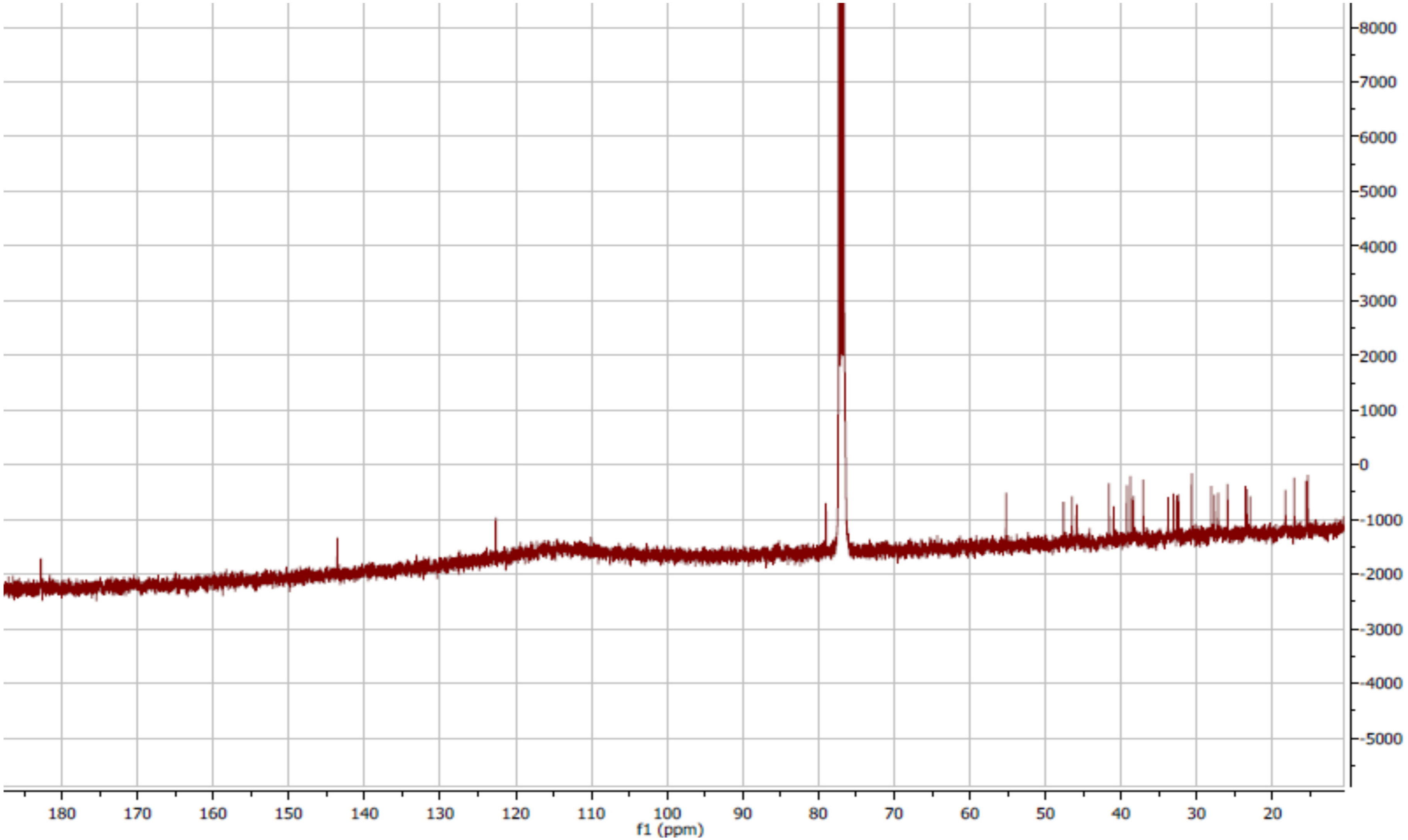

Figure S45. ^{13}C NMR spectrum of **9** in CD3OD (600 MHz)

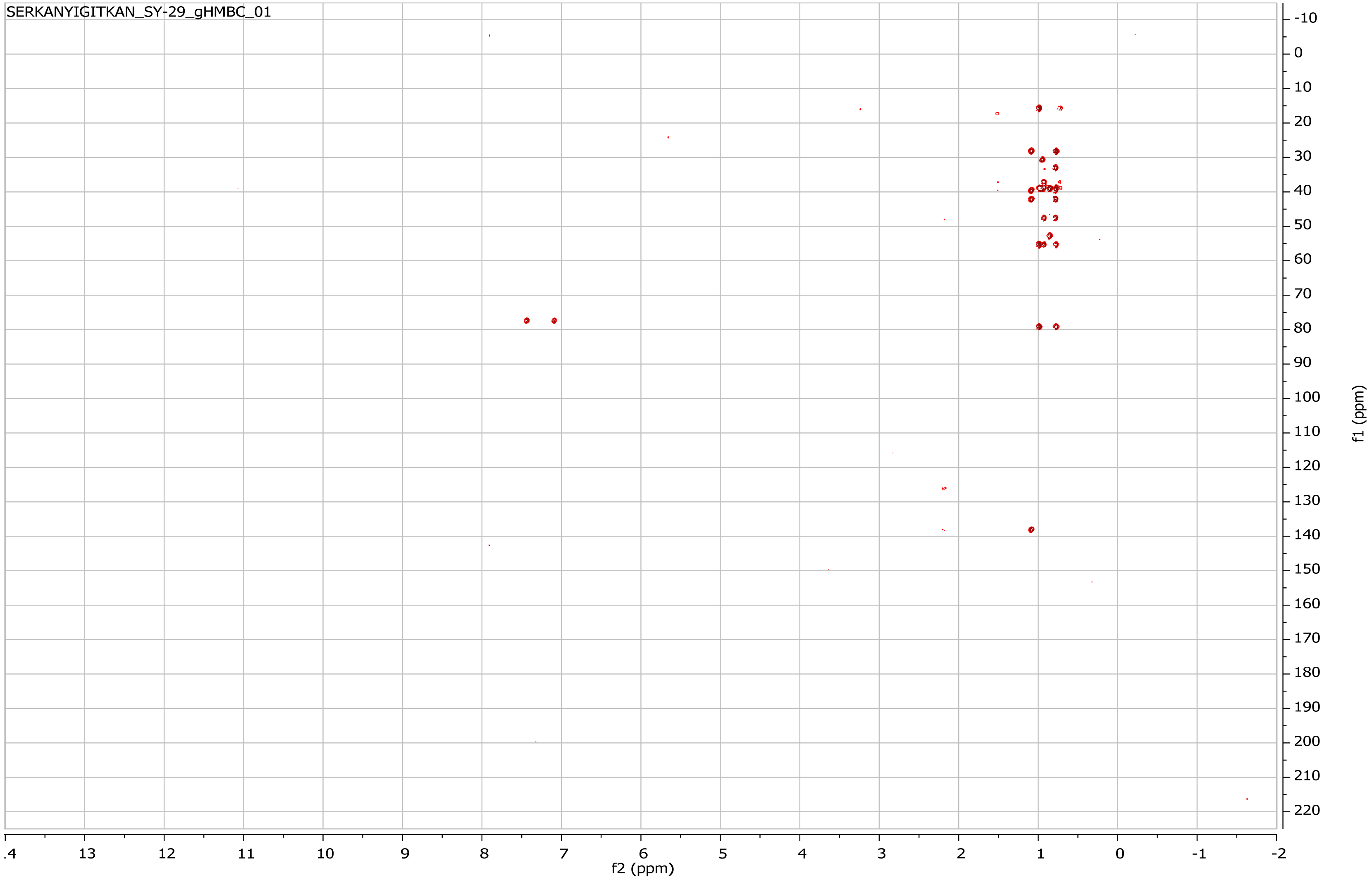

Figure S46. HMBC spectrum of **9** in CD_3OD (600 MHz)

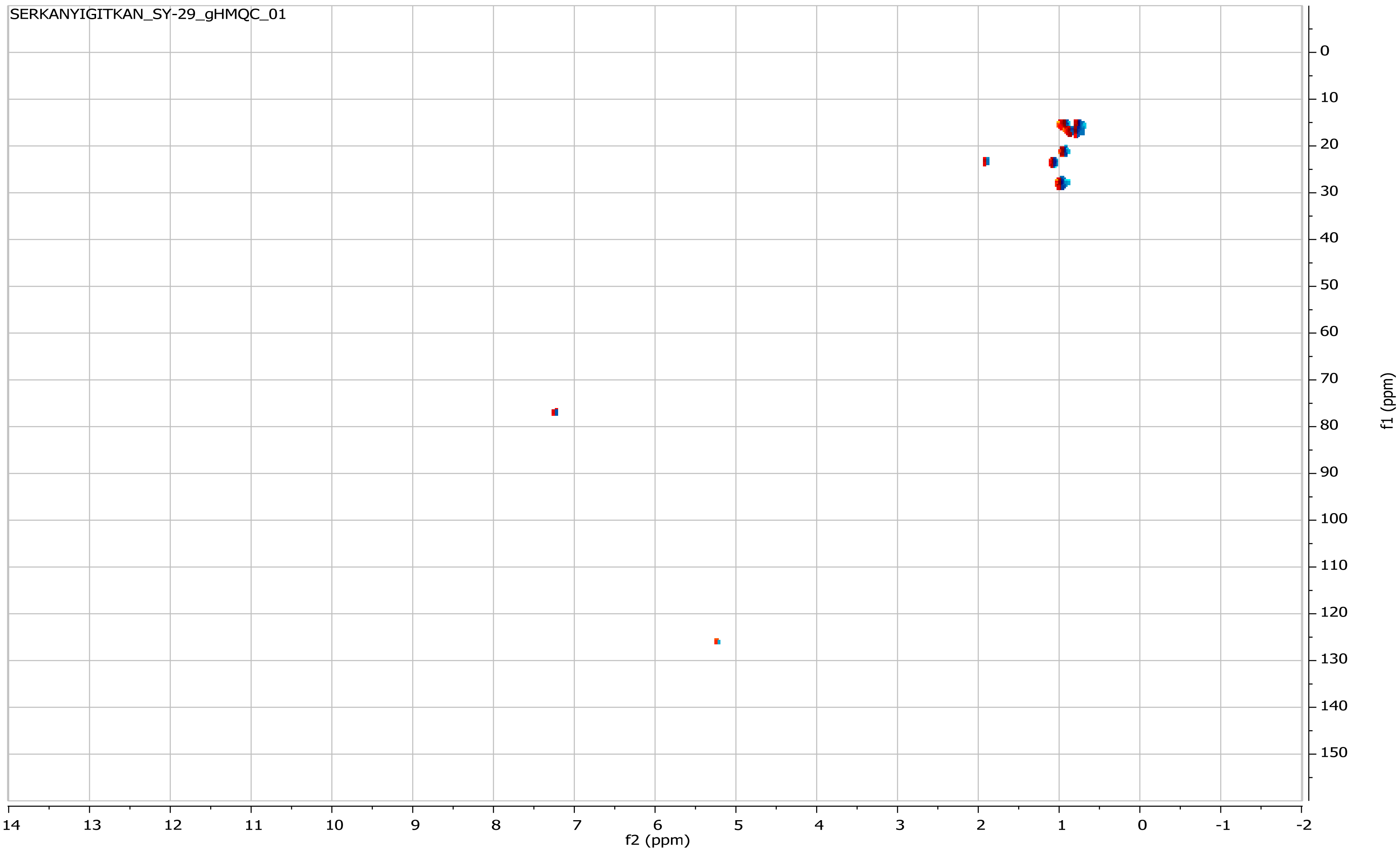

Figure S47. HMQC spectrum of **9** in CD_3OD (600 MHz)

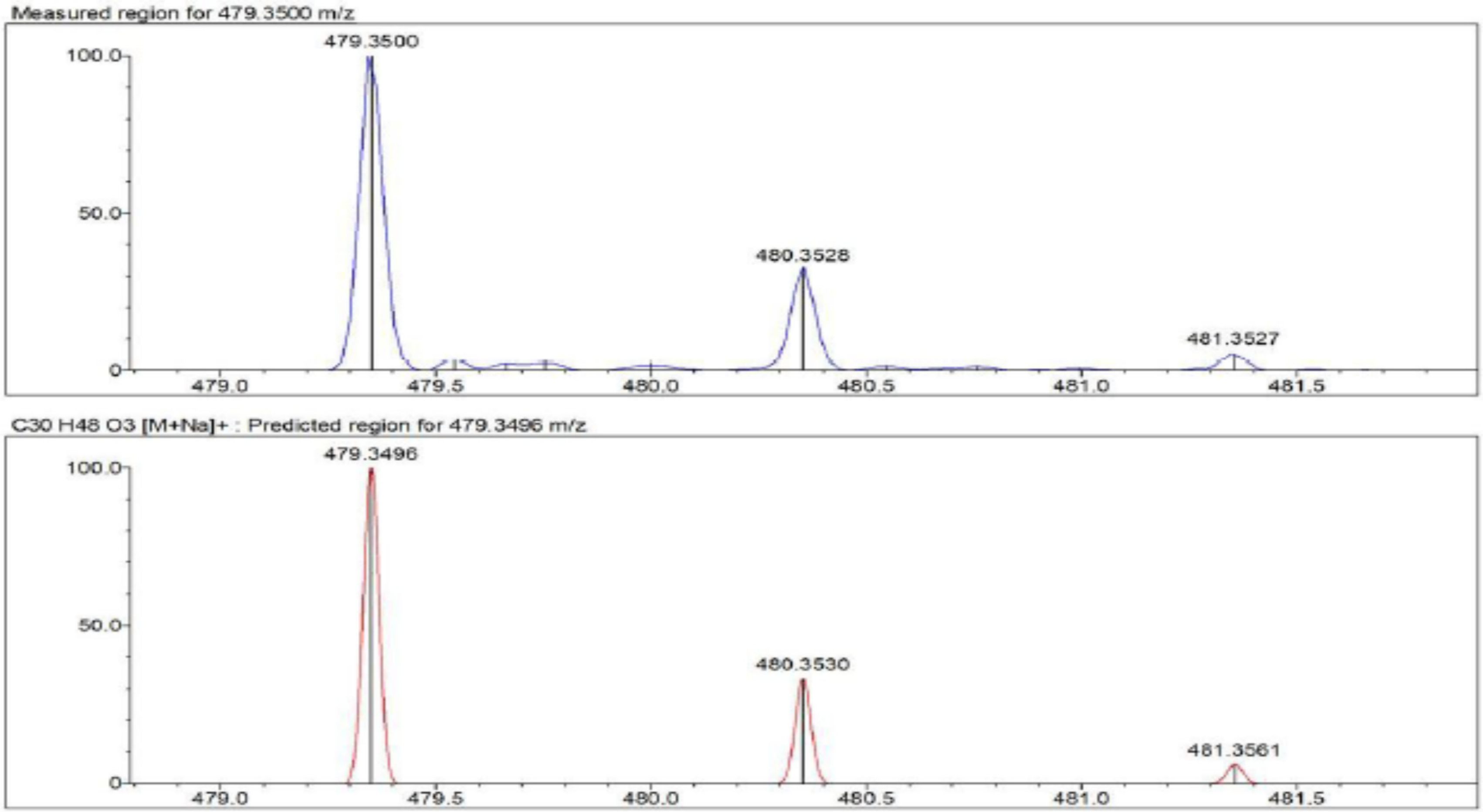

Rank	Score	Formula (M)	Ion	Meas. m/z	Pred. m/z	Df. (mDa)	Df. (ppm)	Iso	DBE
1	96.82	C30 H48 O3	[M+Na]+	479.3500	479.3496	0.4	0.83	96.82	7.0

Figure S48. LC-MS-IT-TOF spectrum of **9**

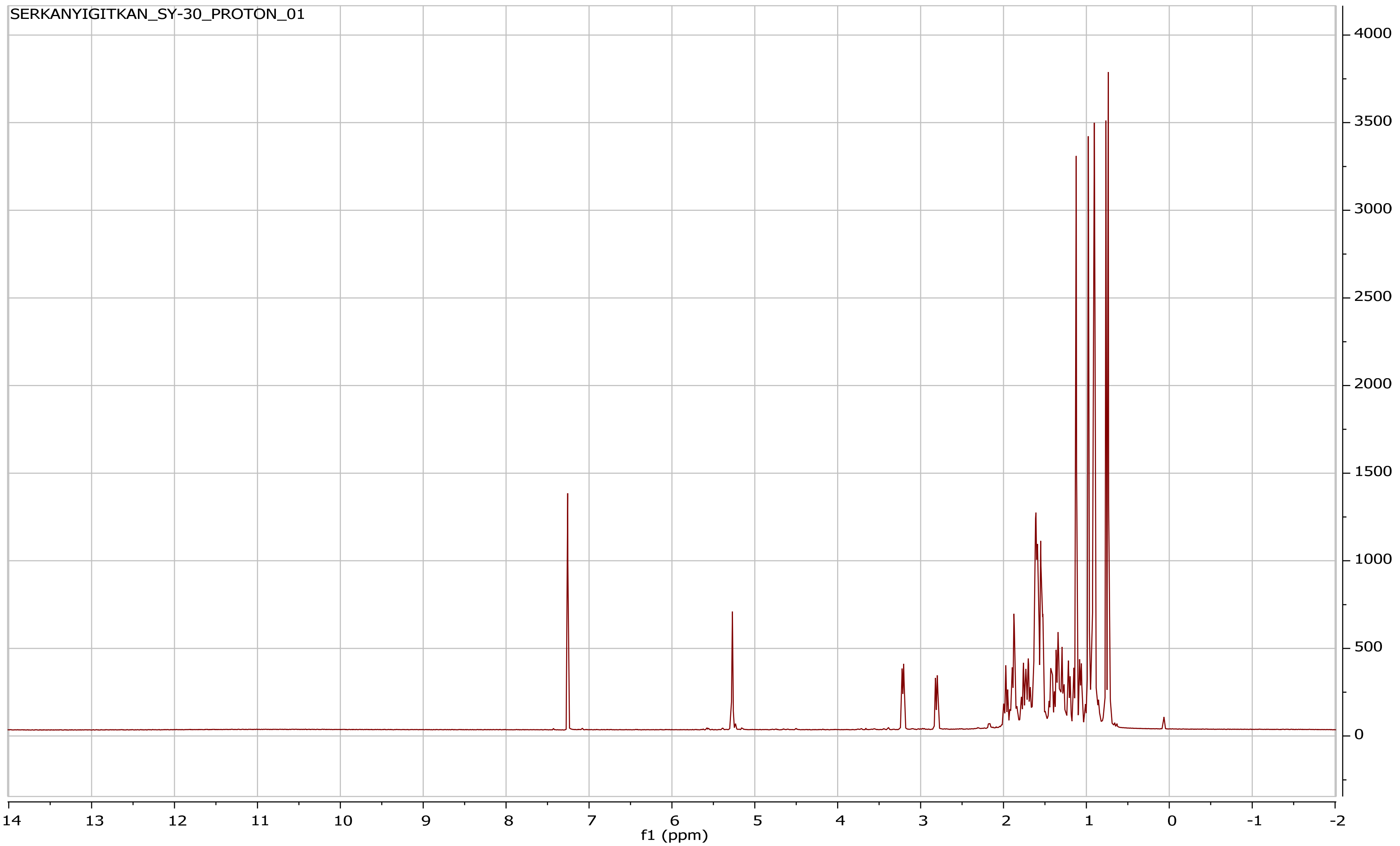

Figure S49. ^{1}H NMR spectrum of **10** in CD_3OD (600 MHz)

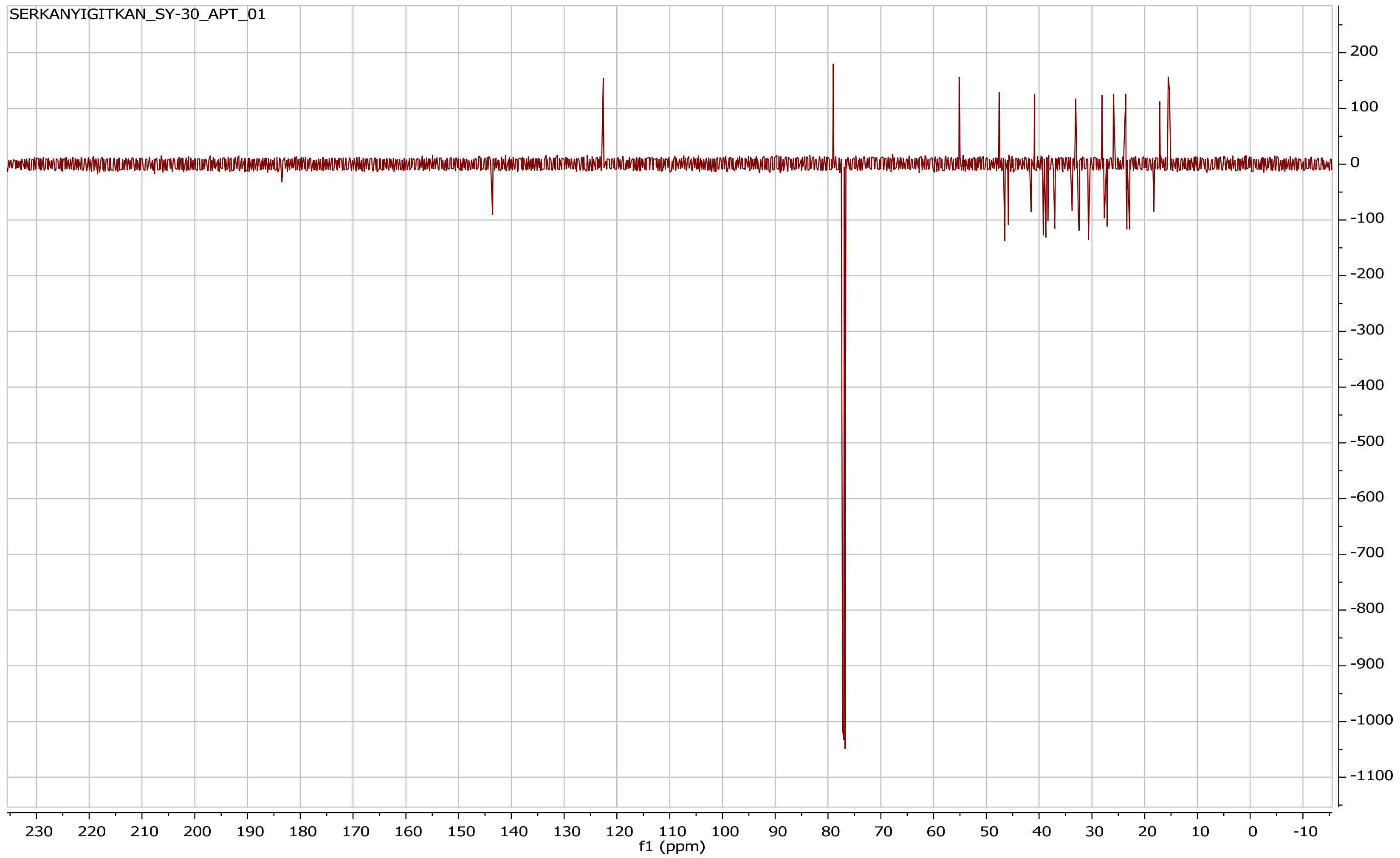

Figure S50. ^{13}C NMR (APT) spectrum of **10** in CD3OD (600 MHz)

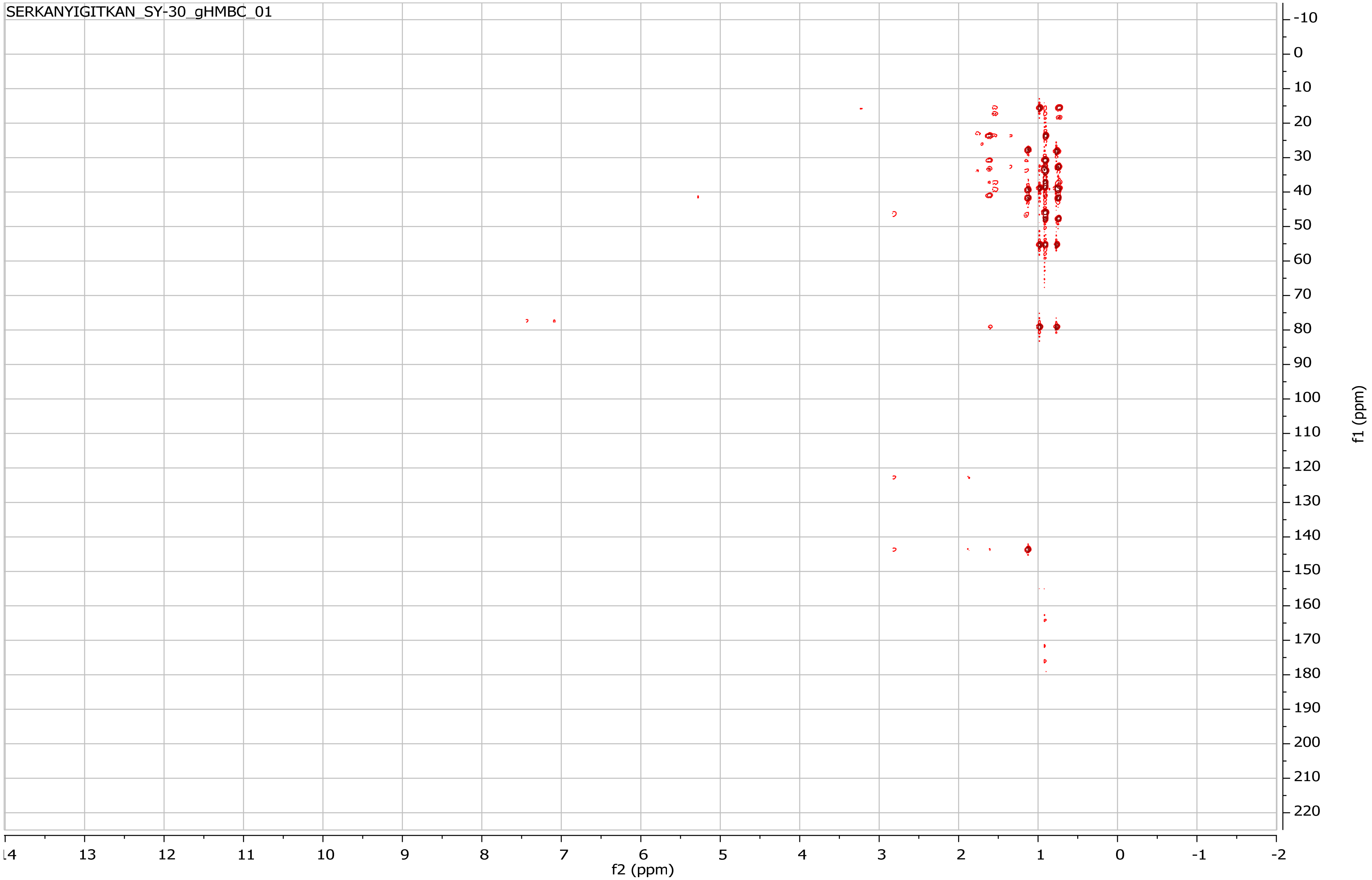

Figure S51. HMBC spectrum of **10** in CD_3OD (600 MHz)

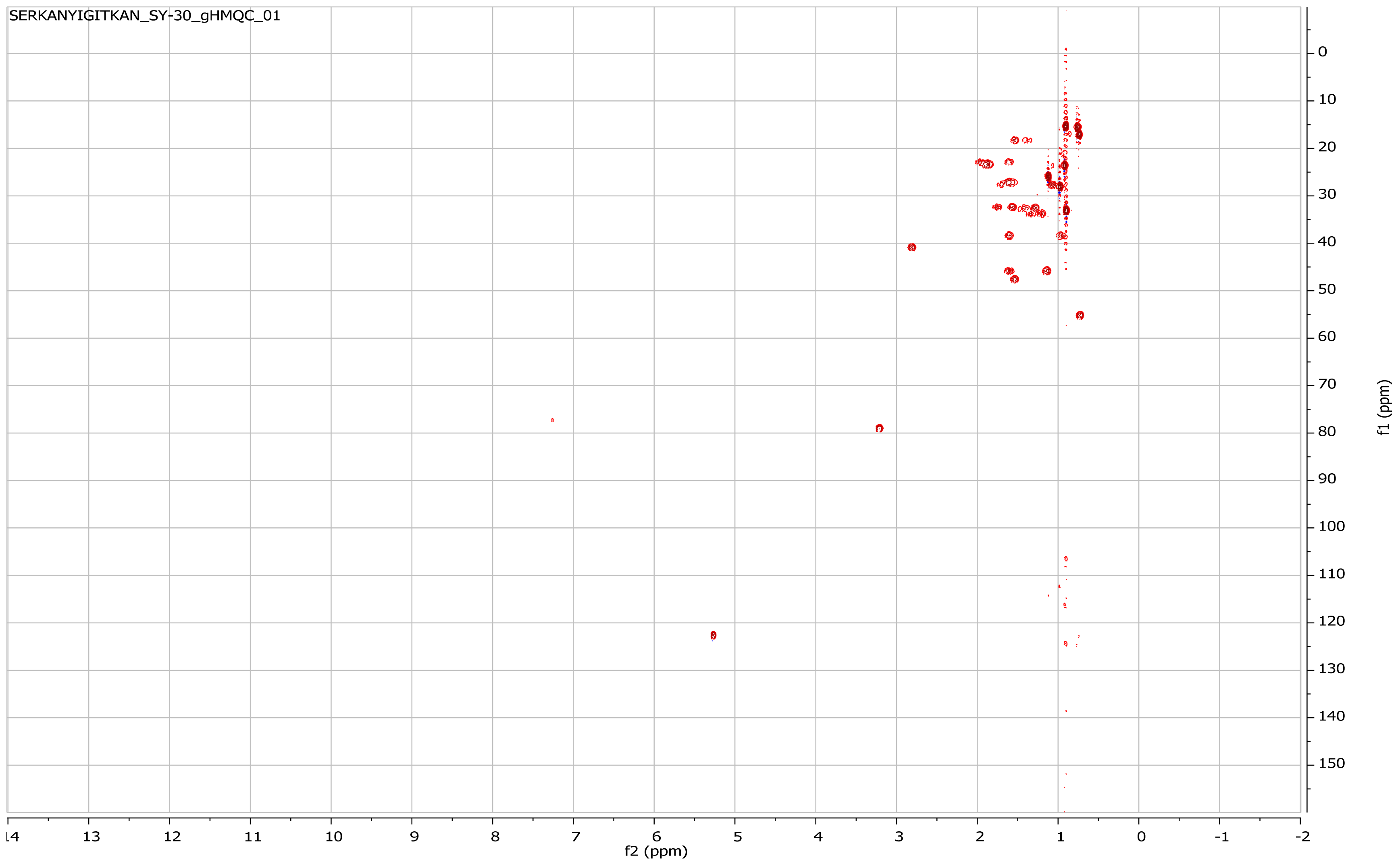

Figure S52. HMQC spectrum of **10** in CD_3OD (600 MHz)

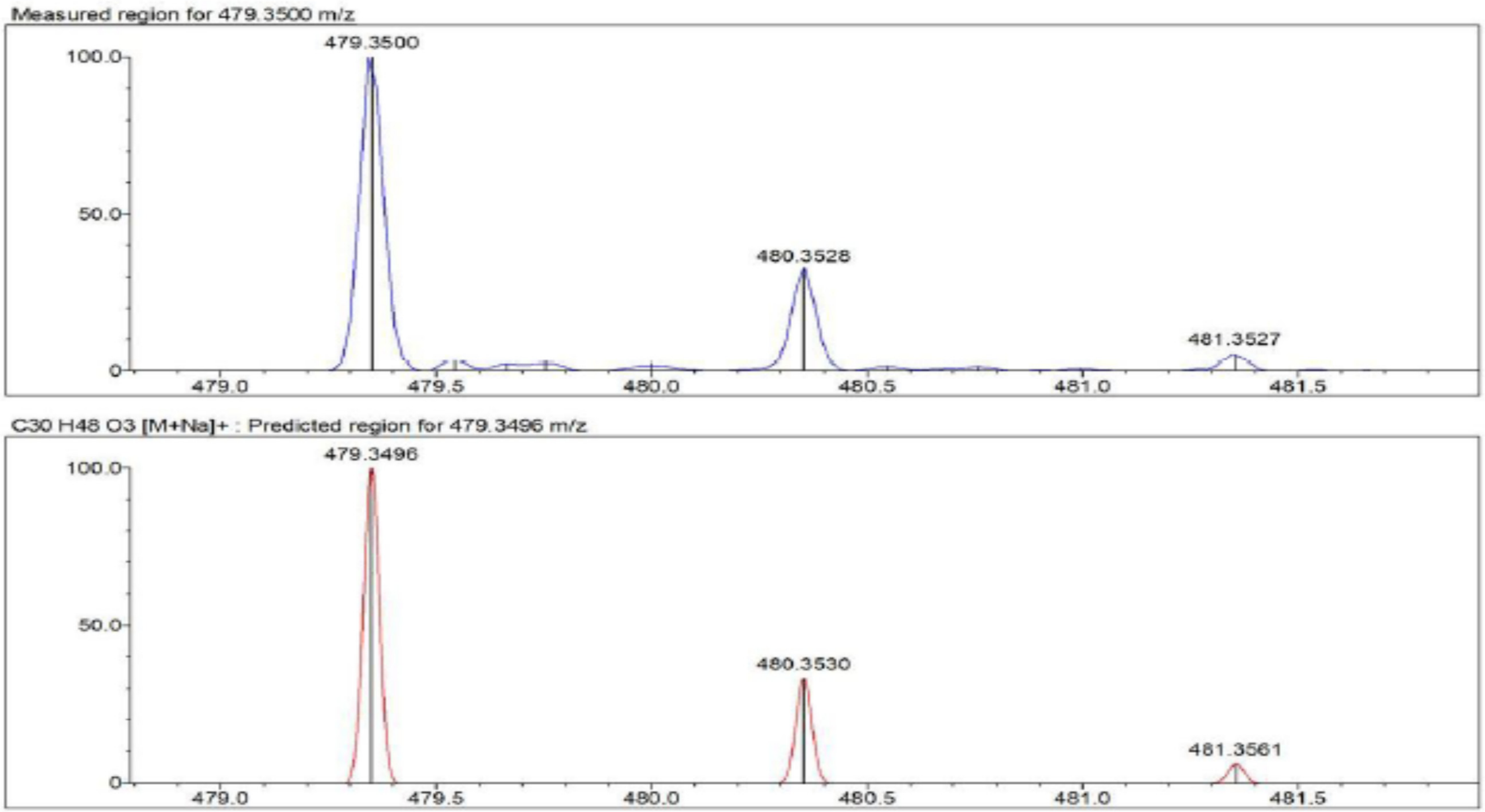

Rank	Score	Formula (M)	Ion	Meas. m/z	Pred. m/z	Df. (mDa)	Df. (ppm)	Iso	DBE
1	96.82	C30 H48 O3	[M+Na]+	479.3500	479.3496	0.4	0.83	96.82	7.0

Figure S53. LC-MS-IT-TOF spectrum of **10.**
